# Bacterial Lactonases ZenA with Noncanonical Structural Features Hydrolyze the Mycotoxin Zearalenone

**DOI:** 10.1021/acscatal.4c00271

**Published:** 2024-02-16

**Authors:** Sebastian Fruhauf, Dominic Pühringer, Michaela Thamhesl, Patricia Fajtl, Elisavet Kunz-Vekiru, Andreas Höbartner-Gussl, Gerd Schatzmayr, Gerhard Adam, Jiri Damborsky, Kristina Djinovic-Carugo, Zbynek Prokop, Wulf-Dieter Moll

**Affiliations:** † dsm-firmenich Animal Nutrition and Health R&D Center Tulln, Technopark 1, Tulln 3430, Austria; ‡ Department for Structural and Computational Biology, Max Perutz Laboratories, University of Vienna, Campus Vienna Biocenter 5, Vienna 1030, Austria; § Institute of Bioanalytics and Agro-Metabolomics, Department of Agrobiotechnology IFA-Tulln, University of Natural Resources and Life Sciences Vienna (BOKU), Konrad-Lorenz-Straße 20, Tulln 3430, Austria; ∥ Institute of Microbial Genetics, Department of Applied Genetics and Cell Biology, University of Natural Resources and Life Sciences Vienna (BOKU), Konrad-Lorenz-Straße 24, Tulln 3430, Austria; ⊥ Loschmidt Laboratories, Department of Experimental Biology and RECETOX, Faculty of Science, Masaryk University, Kamenice 5, Bld. A13, Brno 625 00, Czech Republic; # International Clinical Research Center, St. Anne’s University Hospital Brno, Pekarska 53, Brno 656 91, Czech Republic; ¶ Department of Biochemistry, Faculty of Chemistry and Chemical Technology, University of Ljubljana, Ljubljana 1000, Slovenia; ∇ European Molecular Biology Laboratory (EMBL) Grenoble, Grenoble 38000, France

**Keywords:** zearalenone, mycotoxin, lactonase, carboxylesterase, hydrolase, kinetics, presteady-state, *Rhodococcus erythropolis*

## Abstract

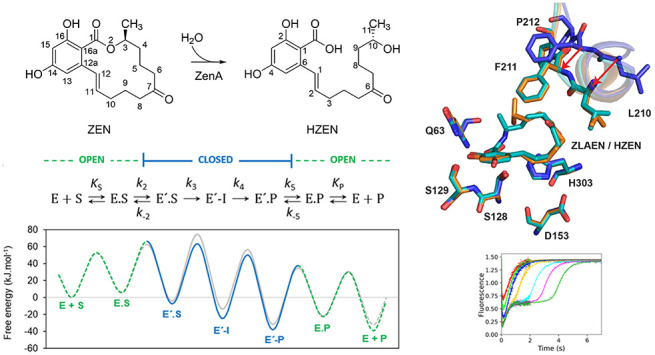

Zearalenone (ZEN) is a mycoestrogenic polyketide produced by *Fusarium graminearum* and other phytopathogenic members of the genus *Fusarium*. Contamination of cereals with ZEN is frequent, and hydrolytic detoxification with fungal lactonases has been explored. Here, we report the isolation of a bacterial strain, *Rhodococcus erythropolis* PFA D8–1, with ZEN hydrolyzing activity, cloning of the gene encoding α/β hydrolase ZenA encoded on the linear megaplasmid pSFRL1, and biochemical characterization of nine homologues. Furthermore, we report site-directed mutagenesis as well as structural analysis of the dimeric ZenA_Re_ of *R. erythropolis* and the more thermostable, tetrameric ZenA_Scfl_ of *Streptomyces coelicoflavus* with and without bound ligands. The X-ray crystal structures not only revealed canonical features of α/β hydrolases with a cap domain including a Ser-His-Asp catalytic triad but also unusual features including an uncommon oxyanion hole motif and a peripheral, short antiparallel β-sheet involved in tetramer interactions. Presteady-state kinetic analyses for ZenA_Re_ and ZenA_Scfl_ identified balanced rate-limiting steps of the reaction cycle, which can change depending on temperature. Some new bacterial ZEN lactonases have lower *K*_M_ and higher *k*_cat_ than the known fungal ZEN lactonases and may lend themselves to enzyme technology development for the degradation of ZEN in feed or food.

## Introduction

**Scheme 1:**
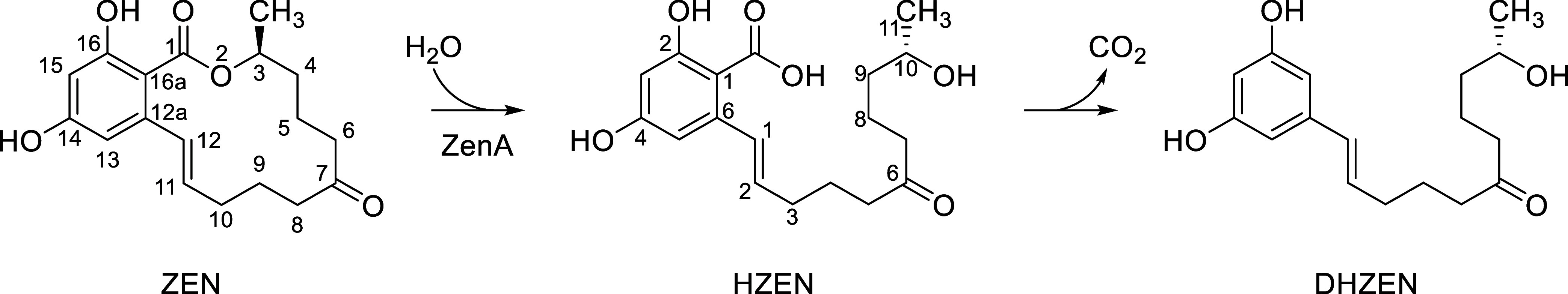
Reaction Catalyzed by ZEN Lactonase ZenA, Followed by Slow, Spontaneous Decarboxylation ZenA cleaves zearalenone [ZEN, (*S*,*E*)-14,16-dihydroxy-3-methyl-3,4,5,6,9,10-hexahydro-1*H*-benzo[c][1]oxacyclotetradecine-1,7(8*H*)-dione] to hydrolyzed zearalenone [HZEN, (*S*,*E*)-2,4-dihydroxy-6-(10-hydroxy-6-oxoundec-1-en-1-yl)benzoic acid], which decarboxylates spontaneously to decarboxylated hydrolyzed zearalenone [DHZEN, (*S*,*E*)-1-(3,5-dihydroxyphenyl)-10-hydroxyundec-1-en-6-one]. Atoms are numbered according to the IUPAC system.

ZEN [(*S*,*E*)-14,16-dihydroxy-3-methyl-3,4,5,6,9,10-hexahydro-1*H*-benzo[c][1]oxacyclotetradecine-1,7(8*H*)-dione, CAS 17924–92–4, Scheme 1]^1^ is a mycoestrogen produced by some fungi of the genus *Fusarium*, especially *Fusarium graminearum* and *Fusarium culmorum*.^2,3^ These *Fusarium* species are plant pathogens and are able to infect several major crop plants, including maize, wheat, barley, and oats. Therefore, ZEN is a frequently found natural contaminant in food and feed around the world.^4^

The estrogenic activity of ZEN is based on its binding interaction with the estrogen receptor ligand-binding domain.^5^ The fungal biosynthesis of ZEN involves two polyketide synthases, which are essential for the biosynthetic condensation of acetate units to form ZEN.^6,7^ A biological function of ZEN for *F. graminearum* is to inhibit mycoparasitism and the growth of competing fungi.^8^ ZEN inhibits fungal and plant heat shock protein 90, like the structurally related radicicol, although at higher concentrations,^9^ but its role as a virulence factor for host plant infection remains uncertain as ZEN production and biosynthesis gene expression are not as high during plant infection as they can be when *Fusarium* grows on certain sterile media.^2,10,11^

Field cases of vulvar hypertrophy displayed by female pigs fed moldy grain motivated the first isolation of ZEN, based on a bioassay of uterine enlargement in mice.^12^ In human and animal nutrition and, especially, pig nutrition, ZEN is considered an undesirable contamination.^13,14^ In the European Union, maximum tolerable levels for ZEN contamination in food were set.^15^ Guidance values for maximum ZEN concentrations in animal feed were also set,^16^ and the European Food Safety Authority (EFSA) published a scientific opinion about the risk of ZEN contamination to animal health.^3^ The FDA has not established guidance values for ZEN. Surveys for several countries showed that ZEN contamination of feed is frequent, and although concentrations are typically low, guidance value concentrations, such as 100 μg ZEN per kg feed for piglets and gilts in the EU, are often reached or exceeded.^4,17^ The concentration of ZEN in cereals is often found to correlate with the concentration of deoxynivalenol (DON), another mycotoxin, which *F. graminearum* produces to facilitate the invasion of plants.

Genuine estrogen signaling is based on a binding interaction between the ligand 17β-estradiol and the nuclear estrogen receptors alpha and beta (ERα and ERβ). The receptors recruit transcriptional coregulators through dedicated binding interfaces, form dimers, and interact with estrogen response elements,^18^ which are conserved sequence elements responsible for the regulation of gene expression. Xenobiotic ligands, including ZEN, induce slightly different structural conformations of ERs and, through various signaling mechanisms, cause diverse endocrine-disrupting activity profiles.^19−21^ The structural difference between the ERα ligand-binding domain with bound estradiol and with bound ZEN has been analyzed in detail.^5^ α-Zearalanol, a derivate of ZEN, is used as a growth promoter for cattle^22^ and has a higher binding affinity to ERs than ZEN.^23,24^ Biological activities of ZEN and its derivatives through mechanisms other than interference with endogenous estrogen signaling were also reported.^25^ Concentrations of certain microRNAs are affected by ZEN and may have the potential for future employment as molecular biomarkers.^26^

ZEN is extensively modified both by plant^27−29^ and by animal detoxification mechanisms. Of particular toxicological relevance is reductive biotransformation to α- or β-zearalenol (ZEL),^30−33^ as α-ZEL has higher estrogenic activity.^34−38^ ZEN undergoes enterohepatic cycling in pigs,^39,40^ chickens,^41^ and rats.^42^ ZEN as well as α- and β-ZEL are glucuronidated and excreted in urine.^32,41,43,44^

ZEN is a stable molecule, and chemical or physical decontamination methods have so far not been practical to implement for the treatment of bulk grain. Feed additives with certain clay minerals or yeast cell wall fragments for the adsorption of ZEN in the gastrointestinal tract of animals are commercially available.^45^ Such binding technology has been pioneered for the mycotoxin aflatoxin, and evidence for the amelioration of aflatoxin exposure is available from feeding trials and analysis of biomarkers.^46,47^

A possible mitigation strategy for the xenoestrogenic activity of ZEN in animals would be the use of an enzyme as a feed additive for the gastrointestinal conversion of ZEN to nonestrogenic reaction products. Such an approach has been successfully implemented with fumonisin esterase for hydrolytic detoxification of fumonisins.^48−50^ ZEN has a lactone ester bond, which is also susceptible to hydrolytic cleavage (Scheme 1). A hydrolase with specificity for ZEN was found to be produced by *Clonostachys rosea*,^51^ and the enzyme, ZHD101, was identified^52^ and characterized,^53^ including its X-ray crystal structure determination.^54,55^ The enzymatic reaction has a preliminary EC number 3.1.1.B12 (https://www.brenda-enzymes.org/). Orthologs are harbored by several other fungi,^56,57^ and activities, kinetic parameters, and crystal structures were determined for some of these sequences.^58−62^ The primary product of enzymatic hydrolysis has long escaped detection because it is too hydrophilic to partition into an organic solvent and was consequently missed when organic extracts were analyzed.^63^ Following the logic of recommended abbreviations for ZEN and derivates,^64^ we use the abbreviation HZEN for the nonestrogenic^65^ hydrolyzed zearalenone and DHZEN for the previously reported, also nonestrogenic secondary reaction product decarboxylated hydrolyzed zearalenone (Scheme 1).^66^ Activities of fungal ZEN lactonases were improved by site-directed mutagenesis, but catalytic efficiency remained too low for practical application as feed additives for enzymatic gastrointestinal degradation of ZEN. Fungal ZEN lactonases were also expressed in probiotic *Lactobacillus reuteri*,^67,68^ but legislation for feed additives does not cover genetically modified microorganisms in many parts of the world. Other fungal ZEN biotransformation mechanisms are also known, namely, sulfation^69^ and monooxygenation.^70^ Mixed bacterial cultures,^71^ as well as strains including a *Pseudomonas* isolate^72,73^ and *Rhodococcus* species,^74−77^ were described for their ability to convert ZEN to nonestrogenic metabolites by unconfirmed reaction mechanisms. *Bacillus* species catalyze the phosphorylation of ZEN,^78,79^ and recently, a bacterial ZEN lactonase was reported.^80^

Here, we report the identification of bacterial ZEN lactonases and their biochemical and structural analysis. The enzymes share less than 20% sequence identity with the previously characterized fungal ZEN lactonases. Kinetic characterization revealed more than 10-fold higher *k*_cat_ and more than 10-fold lower *K*_M_ than for fungal ZEN lactonases, which may make a technological application as animal feed enzymes for gastrointestinal degradation of ZEN feasible.

## Materials and Methods

Specified in the Supporting Information.

## Results

### Cloning of the Gene for ZEN Lactonase ZenA

Several bacterial mixed cultures derived from soil samples and enriched by cultivation with ZEN in Brunner mineral medium with added vitamins (Materials and Methods, Supporting Information) showed ZEN degradation. Such cultures were streaked on agar plates to obtain single colonies, which were cultivated to test their ZEN conversion activity. Isolate PFA D8–1 was obtained by repetition of such streaking and testing and showed fast and robust ZEN conversion activity. Initial 16S rDNA sequencing identified it as *Rhodococcus erythropolis*, and subsequent whole genome sequencing confirmed that PFA D8–1 and the *R. erythropolis* type strain DSM 43066^T^^81^ shared identical genes for 16S rRNA. For stability testing, daughter colonies were generated by plating cell suspension on LB agar plates. All of the 188 cultures derived from such daughter colonies showed hydrolysis of ZEN.

From previous work with ZEN lactonase ZHD101 of *C. rosea*, we already had analytical methods for quantification of HZEN and DHZEN established^45,63^ and found that HZEN was the primary metabolite of ZEN conversion by *R. erythropolis* PFA D8–1 (Figure S1, Supporting Information). *R. erythropolis* DSM 43066^T^ and PR4^82^ showed no hydrolytic activity for ZEN. Cleared lysate of *R. erythropolis* PFA D8–1 also converted ZEN to HZEN, but exposure of the biomass to ZEN before lysis was required, and the activity of lysate from uninduced biomass was marginal (Figure S2, Supporting Information).

Whole-genome sequencing of *R. erythropolis* PFA D8–1 resulted in 9 sequencing scaffolds covering 7.08 Mb (sequence deposited at DDBJ/ENA/GenBank as BioProject PRJNA884193). Seven of these scaffolds aligned well with the *R. erythropolis* PR4 genome,^82^ but scaffolds one and eight, together covering 659 kbp, showed no match. Pulsed-field gradient gel electrophoresis^83^ of *R. erythropolis* PFA D8–1 DNA showed a band at 660 kbp (Figure S3, Supporting Information). Linear megaplasmids are typical of *R. erythropolis* and often harbor genes for the catabolism of xenobiotics.^84^ We named the new megaplasmid pSFRL1 and speculated if genes enabling the degradation of ZEN might be located on pSFRL1. We attempted to isolate pSFRL1 DNA from pulsed-field electrophoresis gels and to clone partially digested pSFRL1 DNA into the *Escherichia coli—Rhodococcus* shuttle vector pMVS301.^85^ However, the cumbersome preparation of pSFRL1 DNA meant that a library of whole genomic DNA was better suited for cloning and functional screening than a library prepared from isolated pSFRL1 DNA.

A genomic library of *R. erythropolis* PFA D8–1 was cloned by isolating genomic DNA, partially digesting it with a restriction enzyme with a 4 basepair recognition site (Hin1II), and ligating fragments into the plasmid vector pMVS301. Screening of this library for ZEN hydrolysis in *E. coli* was not attempted, as complementation of auxotrophic markers in *E. coli* KC8 (*his*, *trp*, *ura*, and *leu*) by transformation of the library could not be shown unequivocally, indicating that even promoters of housekeeping genes of the Gram-positive bacterium were not recognized, and the genes were not sufficiently expressed in *E. coli*. Instead, screening of the library for ZEN hydrolyzing activity in *R. erythropolis* was pursued. The ligation was transformed into *E. coli* DH10B, colonies were washed off plates, and the plasmid library was prepared from such biomass. When the library was transformed into *R. erythropolis* DSM 43066^T^ or PR4 by electroporation,^86^ a bias toward plasmids without inserts or small inserts was noticeable, whereas the library could be transformed back into *E. coli* DH10B without loss of average insert size. A PEG-mediated protoplast transformation protocol^87,88^ was adapted and allowed the transformation of the library into *R. erythropolis* PR4 with an average insert size of 11.5 kbp of *R. erythropolis* PFA D8–1 genomic DNA per clone. Screening of clones for ZEN-hydrolyzing activity resulted in the identification of clone P1G2 with a 7781 bp insert in pMVS301. The insert mapped to pSFRL1 and included a 987 bp ORF (DDBJ/ENA/GenBank accession OAX51_31155) predicted to encode an α/β hydrolase. This ORF was amplified, inserted in pET-28a(+), transformed into *E. coli* BL21(DE3) and HMS174(DE3), and verified to confer ZEN hydrolyzing activity. Following conventions of bacterial gene nomenclature,^89^ we named the gene *zenA*_*Re*_, where the subscript indicates the source organism, *R. erythropolis*.

### Enzymatic and Biophysical Characterization of ZenA_Re_

**Figure 1:**
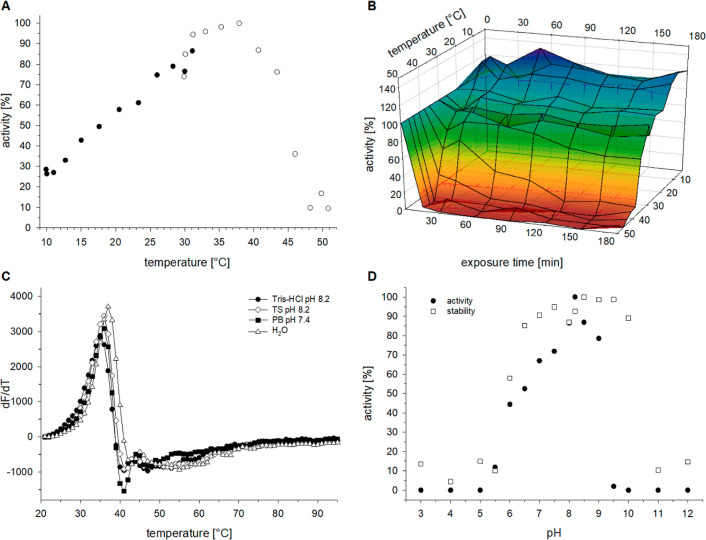
Biochemical characterization of ZenA_Re_. (**A**) Temperature profile of ZenA_Re_ activity. Activity was measured as μM HZEN formed per μM ZenA_Re_ per minute and is shown relative (%) to the highest measured activity. Displayed data is from two sets of measurements covering overlapping temperature ranges. (**B**) Temperature-dependent stability of ZenA_Re_. ZenA_Re_ was incubated in Tris-Cl buffer pH 8.2 with 0.1 mg/mL BSA at various temperatures, time course samples were taken, the residual ZEN hydrolyzing activity was measured, and plotted as % of activity before incubation. (**C**) Thermal denaturation of ZenA_Re_ in different buffers determined with a thermofluor assay. Change of fluorescence intensity over a change of temperature dF/dT is plotted versus temperature. Buffer abbreviations: TS: Teorell-Stenhagen and PB: 20 mM phosphate, 0.5 M NaCl. (**D**) Effect of pH on activity and stability of ZenA_Re_. For pH-stability-correlation, TS buffer was set to the shown pH values, ZenA_Re_ was incubated for 1 h at 25 °C in this set of buffers, and enzyme activity was measured at pH 8.2. For pH-activity-correlation, ZEN hydrolysis at 30 °C was measured directly in the same set of TS buffers. Relative activity indicates μM HZEN generated per minute in the linear part of the HZEN generation time course, displayed relative (%) to the highest measured activity.

A *C*-terminal 6xHis-tag on ZenA_Re_ had no effect on enzyme activity or production yield in *E. coli* (data not shown) and was used to make preparations of pure enzyme. ZenA_Re_ showed the highest activity at 38 °C (Figure 1A), a temperature at which activity declined over the course of incubation (Figure 1B), and in the range of the melting temperature determined with a thermofluor assay (Figure 1C). Activity at 30 °C was highest at pH 8.2, and ZenA_Re_ remained active after incubation in the range from pH 6.5 to pH 10 at 25 °C (Figure 1D). Steady-state kinetic analysis (Figure S4, Supporting Information) determined the kinetic parameters *K*_M_ = 0.34 ± 0.05 μM and *k*_cat_ = 2.9 ± 0.1 s^–1^ for 33 °C and pH 8.2. We followed the Standards for Reporting Enzymology Data (STRENDA, https://www.beilstein-institut.de/en/projects/strenda/) and deposited kinetic data in the database under SRN FZ8UUR.

Sequence alignments allowed prediction of the catalytic serine, present in the conserved signature motif of the nucleophilic elbow, GXSXG^90^ (Ser-128 in ZenA_Re_), and of the catalytic histidine (His-303 in ZenA_Re_). To identify the acidic residue of the catalytic triad, variants of ZenA_Re_ were made by site-directed mutagenesis. Asp-264 of ZenA_Re_, in the sequence range where the acidic residue of the triad is canonically located,^90^ was targeted. Variants D264A, D264L, and D264N were active. Another conserved aspartic acid was also targeted, and none of the enzyme variants with amino acid exchanges D153A, D153L, or D153N showed hydrolysis of ZEN. The catalytic triad of ZenA_Re_ was therefore defined as Ser-128 – His-303 – Asp-153. In α/β hydrolases, oxyanion residue I typically follows the catalytic nucleophile, and oxyanion residue II is located in the loop after β-3.^91^ However, oxyanion residue II is typically preceded by one (G**X** type) or two (GG**G**X type) glycine residues,^92^ and there are no such sequence motifs in the predicted sequence region of ZenA_Re_. There is no tyrosine residue to suggest the presence of a Y-type oxyanion hole.

### Biochemical Analysis of ZenA_Re_ Homologues

**Figure 2:**
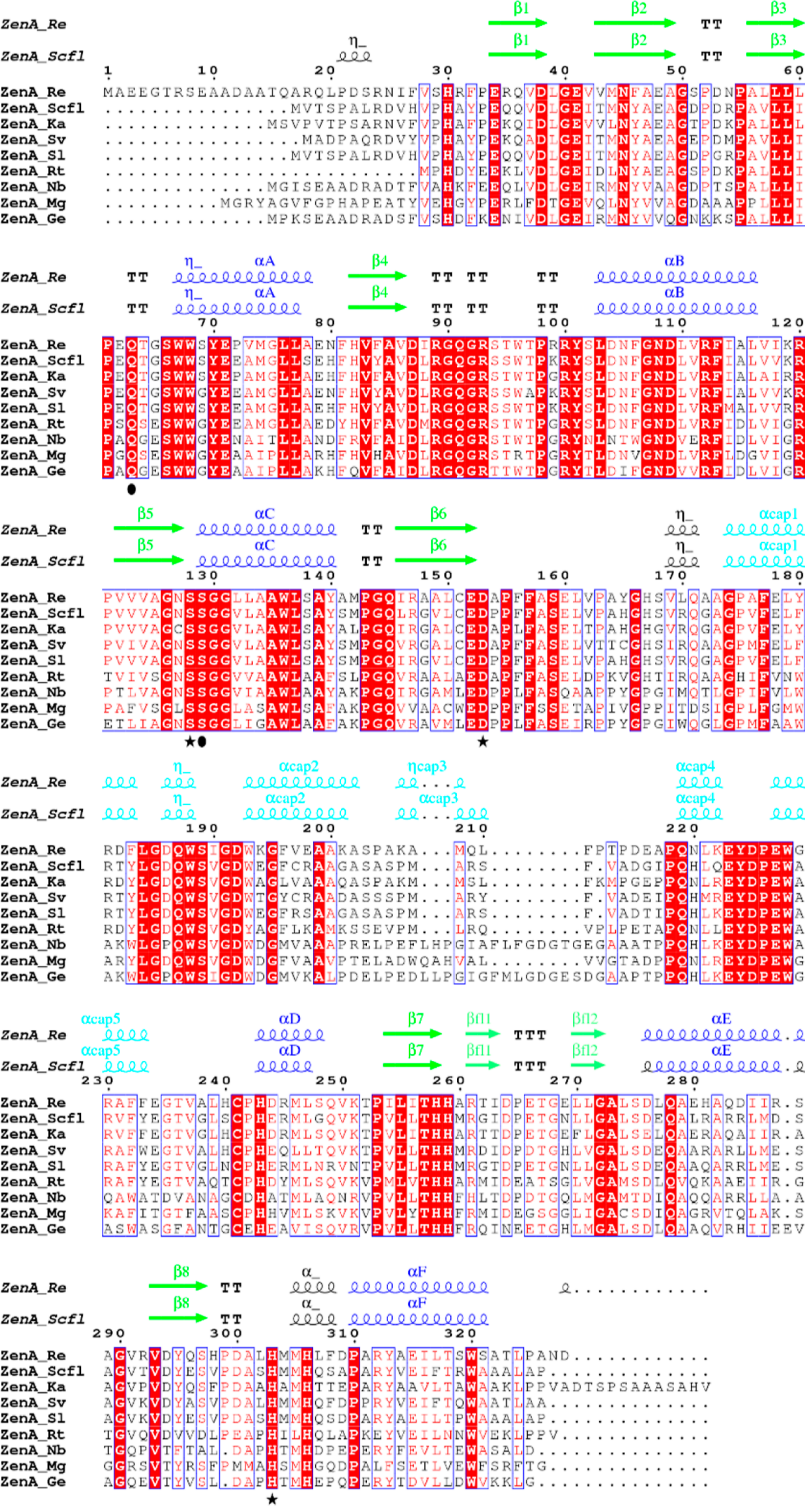
Alignment of ZenA sequences with experimentally verified ZEN hydrolyzing activity. Secondary structure elements shown for ZenA_Re_ (PDB ID: 8CLT) and ZenA_Scfl_ (PDB ID: 8CLO) are from chains A of the PDB files and labeled according to Ollis et al.^90^ with core domain α-helices shown in blue, β-strands in green, and helices of the cap in cyan. β-Strands of the peripheral antiparallel β-sheet (dubbed “the flake”) are labeled fl1 and fl2 and shown in light green. The positions of residues of the catalytic triads are marked with stars, and the positions of residues forming the oxyanion hole are marked with circles. Identical amino acids are shown on a red background. Red letters in blue frames indicate amino acids with more than 70% physicochemical similarity across the set of sequences. TT indicates a β-turn, TTT indicates an α-turn, and η indicates a 3_10_-helix. Figure prepared with ESPript 3.0.^93^

The 14 homologues of ZenA_Re_ listed in Table 1 were chosen from BLAST search results and produced with *C*-terminal 6xHis-tag in *E. coli*. Eight of these enzymes could be produced in soluble form and purified. They all catalyzed the hydrolysis of ZEN, but the measured kinetic parameters varied over a wide range (STRENDA MID 29004; Table 1). A multiple alignment of these sequences is shown in Figure 2. Optimum temperatures ranged from 20 to 50 °C and optimum pH ranged from 7.0 to 8.5. Unfolding temperatures, determined as inflection points of thermofluor fluorescence intensity measured over a temperature ramp, ranged from 38 to 61 °C. Catalytic triad residues of ZenA_Scfl_ were targeted by site-directed mutagenesis. Mutants S112A, D137A, H286A, and H286Y (PDB ID: 8CLP) were well expressed in *E. coli*, and ZEN hydrolyzing activity of the *E. coli* lysates was marginal yet detectable.

**Table 1:**
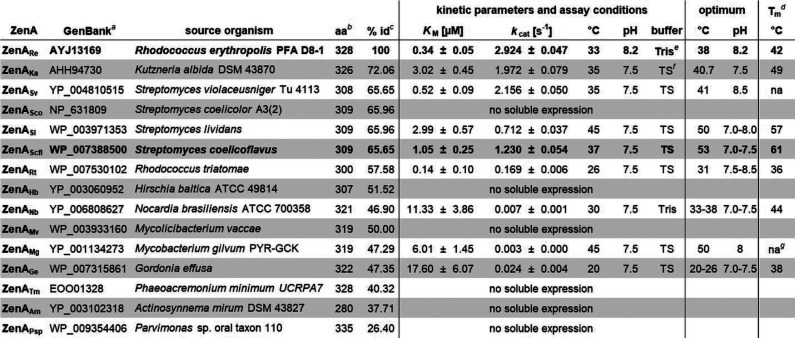
Kinetic Parameters and Enzyme Characteristics of ZenA Homologues

a Accession number in GenBank (https://www.ncbi.nlm.nih.gov/genbank/).

b Amino acids.

c % sequence identity with ZenA_Re_.

d Midpoint of thermal denaturation, determined by thermofluor assay.

e 100 mM Tris-Cl buffer with 0.1 mg/ml BSA.

f Teorell−Stenhagen buffer with 0.1 mg/ml BSA.

g Not available.

### Presteady-State Kinetics of ZenA_Re_ and ZenA_Scfl_

**Figure 3:**
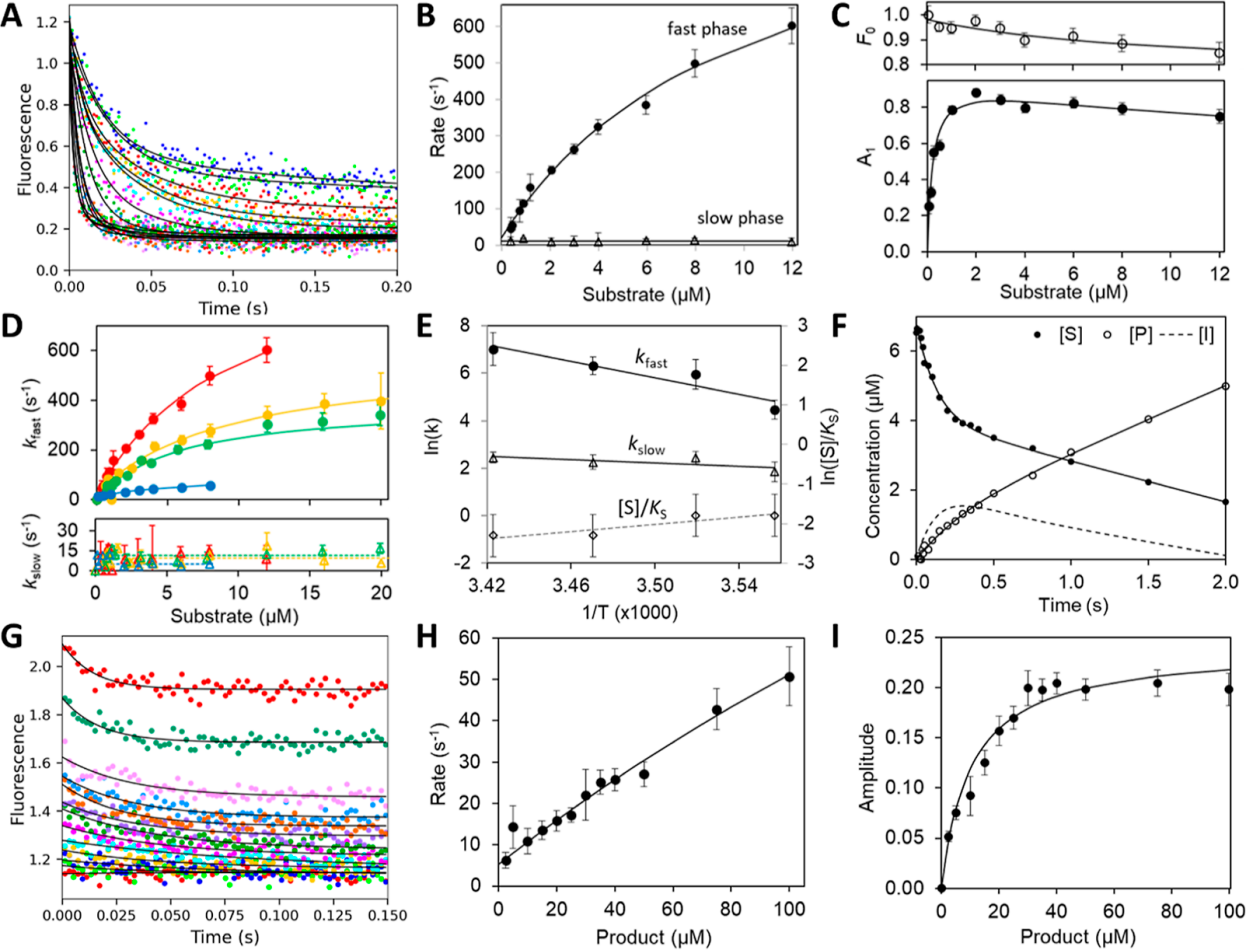
Conventional fitting of ZenA_Re_ kinetic data. The initial phase of the reaction was examined by stopped-flow fluorescence (excitation 275 nm, emission >320 nm) upon mixing 1–20 μM ZEN with 1 μM ZenA_Re_ (final concentrations) at 19 °C. (**A**) Solid lines represent a fit to a double exponential function (eq 1). (**B**) Concentration dependence of the rates of the fast and slow phases derived from fitting data in **A**, with error bars showing the standard errors. (**C**) Concentration dependence of the initial fluorescence (*F*_0_) and the amplitude of the fast phase (A_1_). Solid lines represent the best fit to the hyperbola, and the error bars show the standard errors. (**D**) The concentration dependence of the rates of the initial fast and slow phases of the reaction was analyzed at different temperatures: 8 °C (blue), 11 °C (green), 15 °C (yellow), and 19 °C (red). (**E**) Arrhenius plot of the temperature dependence of *k*_2_ (closed circle) and *k*_3_ (open triangles), providing the initial values of the activation enthalpy terms (*E*_a_) of 150 ± 30 and 30 ± 20 kJ mol^–1^ for *k*_2_ and *k*_3_, respectively. The Van’t Hoff plot of temperature dependence of the equilibrium constant for substrate binding (open diamonds; [S]/*K*_S_), where [S] = 1 μM (physiological standard condition) does not show statistically significant temperature dependence. (**F**) The reaction burst was analyzed using the rapid-quench-flow method upon mixing substrate 6.6 μM ZEN with 2.9 μM ZenA_Re_ (final concentrations) at 11 °C. The solid lines represent the fit of the time course of ZEN and HZEN concentration to eq 4 and eq 5, respectively. (**G**) The stopped-flow fluorescence traces were recorded upon binding of 2.5 to 100 μM HZEN with 1 μM ZenA_Re_ at 8 °C. Solid lines represent a fit to a single exponential function. Concentration dependence of the rate (**H**) and amplitude (**I**) derived from the single exponential fitting data in **G**. Solid lines represent the fit to hyperbola, and error bars show the standard errors.

**Figure 4:**
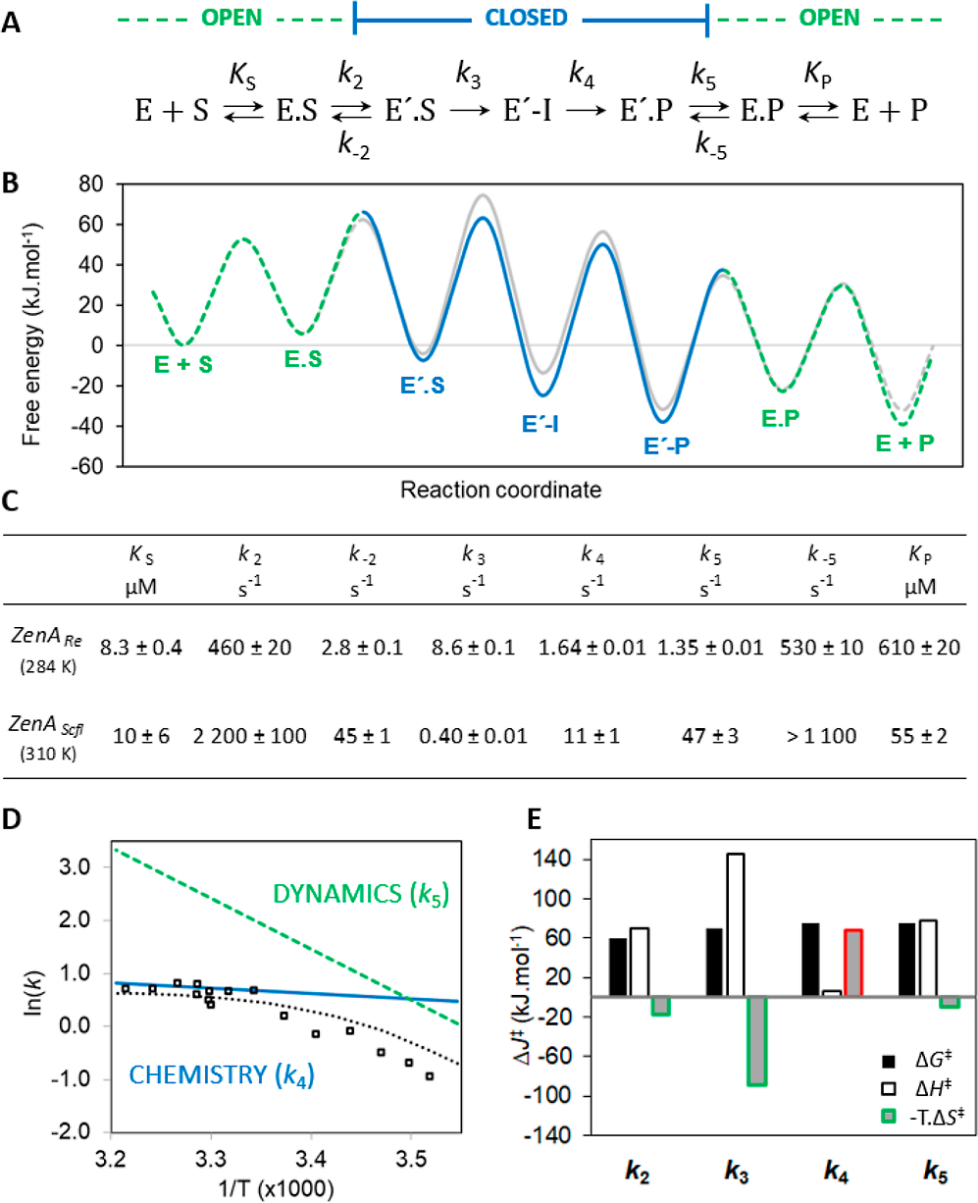
Kinetic mechanism of ZenA. (**A**) The model of the ZenA catalytic cycle where E and E′ are the enzyme in open and closed from, respectively, S is the substrate (ZEN), E.S and E′.S are the enzyme–substrate complex in open and closed from, respectively, E′-I is the covalently bound intermediate, E.P and E′.P are the enzyme–product complex in open and closed from, respectively, and P is the product (HZEN). (**B**) Free energy profiles of the conversion of ZEN calculated using the Eyring equation, Δ*G*‡ = –R.T.ln(k/(kB.T/h)) at reference temperature 310 K, [ZEN] = 1 μM and [HZEN] = 1 μM. Free energy profiles for ZenA_Re_ are shown in color, dashed green and solid blue lines represent open and closed states, respectively, those for ZenA_Scfl_ are presented in gray. (**C**) Kinetic parameters of the catalytic cycle of ZenA_Re_ and ZenA_Scfl_ during ZEN-hydrolysis were obtained by nonlinear regression based on numerical integration of the rate equations derived from the input kinetic model shown in **A**. Standard error (±SE) was calculated from the covariance matrix during nonlinear regression. Reference temperature is 284 K (11 °C) for ZenA_Re_ and 310 K (37 °C) for ZenA_Scfl_. (**D**) Arrhenius plot of ZenA_Re_ experimental activity data (open squares), the temperature dependence of the second chemical step (*k*_4_, blue line)), and the enzyme–product opening step (*k*_5_, dashed green line) simulated using a global numerical model of ZenA_Re_ kinetics; the dotted line is the simulation of ZenA_Re_ activity using the global kinetic model. (**E**) Thermodynamic parameters, the Gibbs free energy of activation at reference temperature 310 K, with entropy and enthalpy contributions, for individual catalytic steps for the reaction of ZenA_Re_.

**Figure 5:**
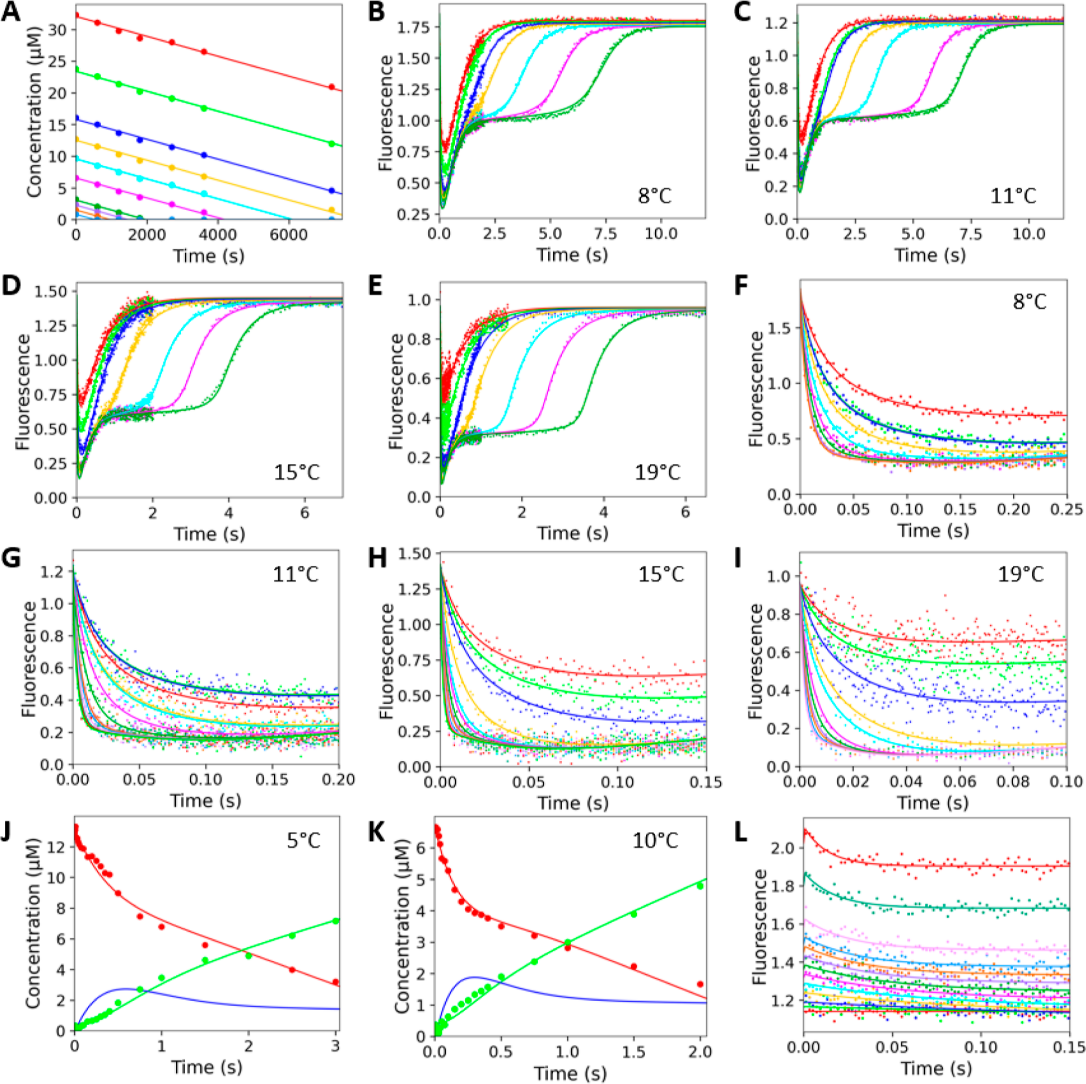
Global numerical analysis of ZenA_Re_ kinetic data. Experiments were conducted using TS buffer at pH 8.2, with data points representing the average of triplicate measurements. All concentrations are the final concentrations after mixing. (**A**) Steady-state data and full–progress curves were recorded during the conversion of 0.8 to 32 μM ZEN by 0.9 nM ZenA_Re_ at 32 °C. Stopped-flow fluorescence (excitation 275 nm, emission >320 nm) full conversion data were recorded upon mixing 0.8 to 4 μM ZEN with 1 μM ZenA_Re_ (final concentrations) at 8 (**B**), 11 (**C**), 15 (**D**), and 19 °C (**E**). The stopped-flow fluorescence traces of the initial substrate binding were recorded upon mixing (final concentrations) 1 to 7 μM ZEN with 1 μM ZenA_Re_ at 8 °C (**F**), 1 to 18 μM ZEN with 1 μM ZenA_Re_ at 11 °C (**G**), 0.5 to 27 μM ZEN with 1 μM ZenA_Re_ at 15 °C (**H**), and 0.5 to 8 μM ZEN with 1 μM ZenA_Re_ at 19 °C (**I**). (**J**) Rapid-quench flow reaction burst analyzed upon mixing 13 μM ZEN with 6 μM ZenA_Re_ at 5 °C and (**K**) mixing 6.5 μM ZEN with 3 μM ZenA_Re_ at 10 °C (final concentrations). Red and green lines represent the global fitting of the substrate and product concentration data. Blue line represents the simulation of the concentration of the reaction intermediate. (**L**) Stopped-flow fluorescence analysis of product binding was recorded upon mixing 2.5 to 100 μM HZEN with 1 μM ZenA_Re_ (final concentrations) at 8 °C. Solid lines represent global fitting to the kinetic data.

ZenA_Re_, as our primordial ZEN lactonase with high activity, and ZenA_Scfl_ with high temperature stability, were chosen for presteady-state kinetic analysis using stopped-flow and quench-flow methods to unveil the kinetic mechanism of ZEN-hydrolysis and to estimate the rate and equilibrium constants related to individual catalytic steps. As the reaction was too rapid at a physiological temperature of 37 °C, we performed kinetic experiments with ZenA_Re_ at progressively lower temperatures, enabling us to precisely quantify each step and get a deeper understanding of the reaction thermodynamics. We started the kinetic analysis with conventional analytical curve fitting to develop a kinetic model and obtain initial estimates of some of the rate and equilibrium constants (Figure 3 and Table S1, Supporting Information). In a second step, the results from the conventional analytical fit were then used as initial parameters for a complex global numerical model (Figures 4 and 5). Overall, the kinetic analysis employed a complex dataset, including time-resolved information about the absolute concentration of molecular species from quench-flow burst and steady-state data and time-resolved spectroscopic data recorded by using stopped-flow fluorescence methodology. Natural tryptophan fluorescence provided a wealth of information on all individual catalytic steps without the need for labeling. Control injections with enzyme only and substrate only were performed to verify that observed signals can be attributed to enzyme–substrate interactions rather than artifacts, and recordings are shown in Figure S5 (Supporting Information). The contributions of individual reactive species to the fluorescence signal were first identified and quantified within the analytical data fit (Figure 3), and then consistently used to construct an appropriate scaling function for numerical fitting. We described step-by-step how individual fluorescence contributions were identified and quantified, and how the final scaling function used to simulate the fluorescence kinetic data was built in the Supporting Information text, “*Kinetic Data Analysis and Statistics*”.

The following text describes in detail the initial conventional analytical fitting. During the first 200 ms of the reaction of ZenA_Re_ at 19 °C monitored by the stopped-flow fluorescence method (Figure 3A), the fluorescence intensity was strongly quenched, exhibiting two kinetic phases that fit a double exponential function (eq 1).
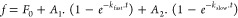
1The concentration dependence of the rate of the fast phase (*k*_fast_) indicates that the substrate binding undergoes a two-step process, a rapid equilibrium formation of the initial enzyme–substrate complex followed by an induced conformational change (Figure 3B). Fitting a hyperbola (eq 2) to the concentration dependence of *k*_fast_ provided first estimates of the dissociation constant for the initial enzyme–substrate complex (*K*_S_ = 10 ± 2 μM), the rate of the conformational step (*k*_2_ = 1000 ± 100 s^–1^), and the sum of the rates of the steps breaking down the final closed enzyme–substrate complex (*k*_sum_ = *k*_–2_ + *k*_3_ = 21 ± 12 s^–1^). The second slow phase (*k*_slow_) provided an initial estimate of the rate of the following chemical step (*k*_3_ = 12 ± 1 s^–1^). The *k*_sum_ and *k*_3_ values indicate that *k*_–2_ ≪ *k*_2_, and the fast-induced conformational change strongly contributes to the overall affinity to the substrate. The net dissociation constant for the two-step binding (*K*_S,net_ = 0.2 μM, eq 3) is 50-times lower (means 50-times tighter binding) compared to the true dissociation constant of the initial enzyme–substrate collision complex (*K*_S_ = 10 ± 2 μM). The value of *K*_S,net_ calculated from the concentration dependence of *k*_fast_ rate is nearly identical to the value of *K*_S,net_ = 0.21 ± 0.04 μM obtained by fitting the concentration dependence of the amplitude (A_1_) of the fast phase (Figure 3C), providing an additional indication for the consistency of the analytical fitting.
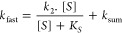
2
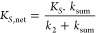
3

This analysis was repeated at 15, 11, and 8 °C (Figure 3D), providing additional information on the temperature dependence of the kinetic parameter estimates. The temperature dependence of the estimated rates and substrate dissociation constant (Figure 3E) do not indicate notable deviation from linearity (ln scale versus 1/T), thus a change in heat capacity does not need to be considered in further analysis.

To verify the correct interpretation of stopped-flow fluorescence signals and to gain deeper insights into the following chemical step(s), we conducted reaction burst experiments under substrate-saturating conditions with quench-flow instrumentation. A strong burst in substrate consumption was observed, indicating the presence of a reaction intermediate (Figure 3F). Additionally, a slight burst in product formation was also indicated, which suggested that a second chemical step is followed by a slow, rate-determining step associated with product release. By fitting the rapid-quench bursts data to eqs 4 and 5, respectively, we obtained rates of the substrate and product burst phase, *k*_S,burst_ = 7.6 ± 0.7 s^–1^ and *k*_P,burst_ = 3.9 ± 0.9 s^–1^, which are mainly determined by the rates of the two chemical steps *k*_3_ and *k*_4_, respectively. The rate of the steady-state phase *k*_SS_ = 1.7 ± 1.0 s^–1^ provides a rough estimate of the rate-limiting step associated with product release (*k*_5_).
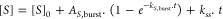
4
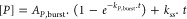
5To explore the last step of the reaction, we performed a stopped-flow analysis of the product binding. The intrinsic tryptophan fluorescence traces, recorded at 8 °C, showed a single exponential decay (Figure 3G). Analogously, as for the substrate, we performed analytical fitting of the rate and amplitude (Figure 3H and I) to obtain initial estimates for the dissociation constants of the enzyme–product complex (*K*_P_ = 480 ± 350 μM) and rates of the induced conformational change *k*_–5_ = 270 ± 160 s^–1^ and *k*_5_ = 5 ± 1 s^–1^. The value of *K*_P,net_ = 9 μM calculated similarly as for substrate binding from the concentration dependence of the rate (eq 3) corresponds well with the value *K*_P,net_ = 10 ± 1 μM obtained by fitting hyperbola to the concentration dependence of the amplitude (Figure 3I). We also tested the possibility of slow binding of the product, but the *K*_P_ value obtained from the linear model of the dependence of the rate on the concentration did not correspond to the value obtained from fitting the amplitude. Thus, the analysis of the concentration dependence of rate and amplitude supported the two-step mechanism for the product binding and the significant effect of ligand-induced conformational changes, as is the case for substrate binding.

The step-by-step analysis described above made it possible to define a model of the ZenA catalytic cycle (Figure 4A), which consists of (1) substrate binding, (2) an induced conformational step (enzyme “closure”), (3) the first chemical step (intermediate formation), (4) the second chemical step (intermediate to product conversion), (5) the conformational step of the enzyme–product complex (enzyme “opening”) preceding (6), the last product release step.

Although the analytical fitting provides valuable information on the mechanism, particularly through the analysis of the concentration dependence of the rates and amplitudes, it is limited in its ability to provide precise parameter estimates due to approximations and error accumulation during multistep fitting.^94^ To overcome these limitations, we modeled the steady-state and presteady-state kinetic data globally using numerical integration of the rate equations derived from the kinetic model of ZenA (Figure 4A). The parameters from the analytical fit (Table S1, Supporting Information) were used as starting values for the global fit. In the global analysis, the full dataset was fit simultaneously (Figure 5) to derive a single set of rate and equilibrium constants and activation enthalpy terms for individual catalytic steps (Table S2 andFigure 4C, Supporting Information). In addition to conventional regression analysis and estimation of standard errors, global analysis of the kinetic data allows a rigorous analysis of the variance, reported herein as confidence contour analysis.^95^ Such an analysis confirms the high quality of the global fit, with all obtained kinetic and thermodynamic parameters being well constrained by the experimental data (Figure S6, Supporting Information). It is also important to mention that the parameters obtained by the two different approaches, conventional analytical fitting and global numerical integration, show a high degree of agreement and thus provide an important indication of the overall validity of the presented kinetic model.

**Figure 6:**
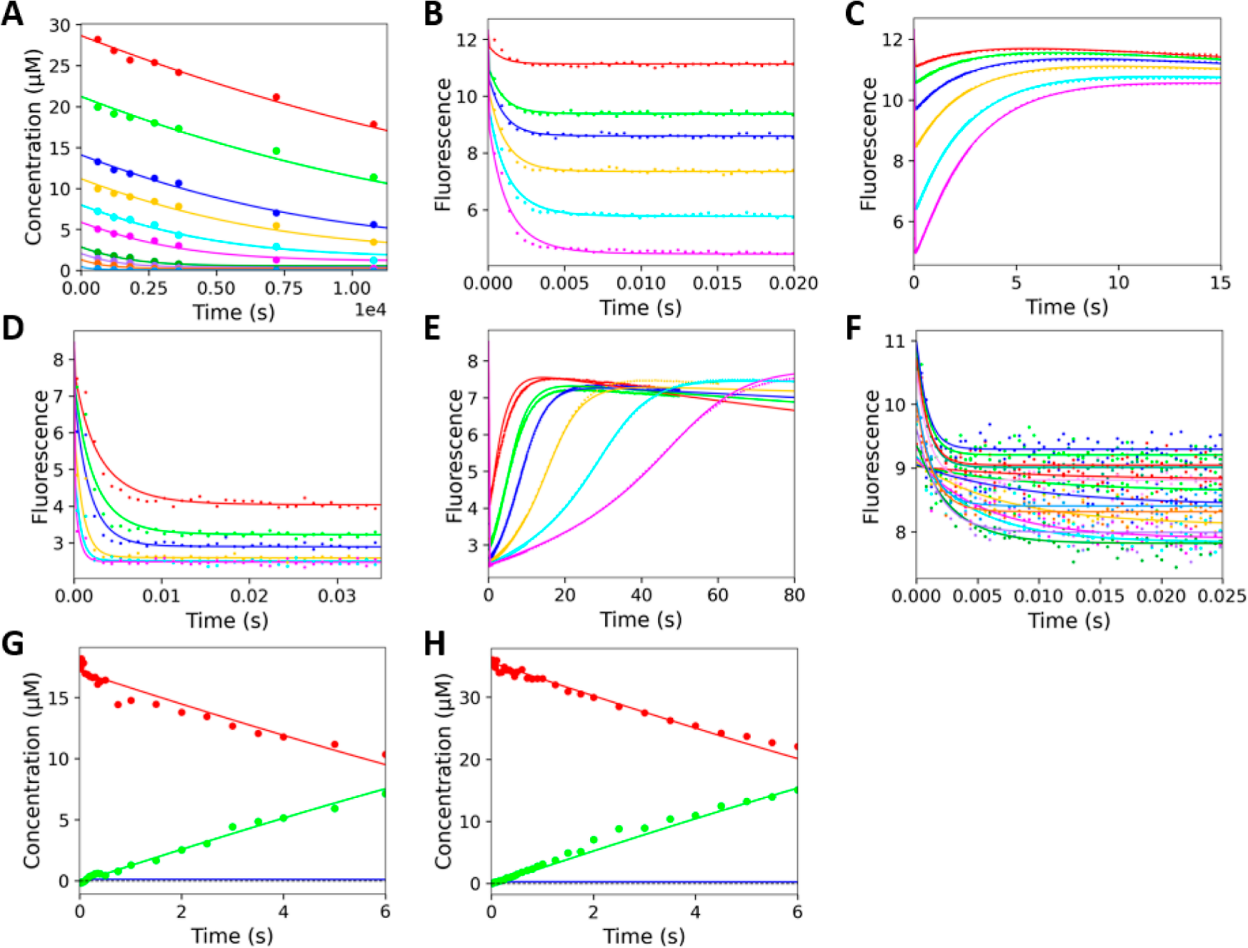
Global numerical analysis of ZenA_Scfl_ kinetic data. Experiments were conducted using TS buffer pH 7.5 at 37 °C, with data points representing the average of triplicate measurements. All concentrations are the final concentrations after mixing. (**A**) Steady-state data and full–progress curves were recorded during the conversion of 0.5 to 28 μM ZEN by 3 nM ZenA_Scfl_. (**B** and **C**) Single turnover stopped-flow fluorescence (excitation 275 nm, emission >320 nm) data were recorded upon mixing 1.7 to 12 μM ZEN with 12 μM ZenA_Scfl_. (**D** and **E**) Multiple turnover stopped-flow fluorescence traces were recorded upon mixing 2 to 35 μM ZEN with 2 μM ZenA_Scfl_. (**F**) Stopped-flow fluorescence analysis of product binding was analyzed upon mixing 0.5 to 75 μM HZEN with 2 μM ZenA_Scfl_. Solid lines represent global fitting to the kinetic data. The reaction burst was analyzed using the rapid-quench-flow method upon mixing substrate (concentrations resulting from the best fit of the global model and nominal concentrations in brackets) 17.1 μM ZEN (16.7 μM) with 3.6 μM ZenA_Scfl_ (3.5 μM) at 37 °C (**G**) and 35.6 μM ZEN (35 μM) with 7.3 μM ZenA_Scfl_ (7.5 μM) at 37 °C (**H**). Red and green lines represent the global fitting of the substrate and product concentration data. Blue line represents the simulation of the concentration of the reaction intermediate.

We performed a similar kinetic analysis for ZenA_Scfl_. The conventional analytical fit of substrate and product titration data (Figure S7, Supporting Information) provided important information on the mechanism and made it possible to obtain the initial parameters of individual kinetic steps (Table S1, Supporting Information). These were further used as starting values for more rigorous global fitting, where stopped-flow and quench-flow data were again combined for intrinsic verification, robustness, and interpretation of measured kinetic events as steps of the reaction mechanism. The existence of a reaction intermediate was also confirmed for the reaction of ZenA_Scfl_. Although the two chemical steps for the formation and decay of the intermediate were kinetically merged at 37 °C (Figure S7J, Supporting Information), analysis at low temperatures allowed these two steps to be separated and unambiguously identified (Figure S7K, Supporting Information). The following global numerical analysis (Figure 6) provided a single set of rate and equilibrium constants for individual catalytic steps of the proposed kinetic model (Table S2 and Figure 4C, Supporting Information). The robustness of the model and the accuracy of the obtained kinetic parameters were verified by confidence contour analysis (Figure S8, Supporting Information).

Both ZenA_Re_ and ZenA_Scfl_ exhibit similar kinetic behavior and support a common kinetic mechanism (Figure 4A,B). The dynamic behavior of the enzymes induced by the presence of ligands plays a critical role in their catalytic cycle. Upon binding of the substrate, the induced fit increases the enzyme’s affinity by approximately 50 times. The reopening of the enzyme–substrate complex occurs very slowly, which helps to securely hold the substrate in a closed, reactive position. Notably, the mechanism of product release and its reaction coordinate mirror those of substrate binding (Figure 4B). In other words, the enzyme–ligand complex must overcome a significant barrier to open, but once it does, the dissociation of the product occurs rapidly. Similarly to substrate binding, the net dissociation constant of the enzyme–product complex (*K*_P,net_) is significantly enhanced by the induced conformational change. On the one hand, the conformational dynamics of the enzyme allow for high affinity to the substrate; on the other hand, they complicate the release of the product. In the case of the ZenA_Re_ reaction at low temperature, the opening of the enzyme associated with the release of the product is even the rate-limiting step. However, as the temperature increases, the enzyme opening becomes easier, which leads to another step, hydrolysis of the intermediate, becoming rate-limiting. This shift causes the nonlinearity of the temperature dependence of catalytic activity [ln(k) vs 1/T], which can be accurately predicted by a global numerical model taking into account the temperature dependencies of the two limiting processes: the chemical step, which is less affected by change of temperature, and the conformational change associated with product release, which is more affected by change of temperature (Figure 4D). The change in the rate-limiting step is also observable from the data obtained with ZenA_Scfl_. Rapid-quench analysis performed at 37 °C indicated no initial burst phase for either substrate consumption or final product formation (Figure S7J, Supporting Information), suggesting that the rate-limiting step is in the first chemical step. However, when the temperature was reduced to 10 °C, a clear burst phase appeared for substrate consumption, but no burst phase was still observed for product formation (Figure S7K, Supporting Information). This indicates a rate limitation in the second chemical step. Hence, the operating temperature can significantly influence the catalytic properties of these enzymes.

The ZenA_Re_ dataset, which includes measurements performed at multiple temperatures, allowed us to additionally derive the Gibbs free energy of activation, along with estimates of the entropy and enthalpy contributions. No statistically significant trend was observed for the temperature dependence of the weak dissociation constant *K*_S_ (Figure 3E). Likewise, the global numerical model did not indicate any statistically significant temperature dependence for either equilibrium constants *K*_S_ or *K*_P_. A detailed thermodynamic analysis was thus limited to the conformational and chemical steps. For both chemical steps, the Gibbs free energy of activation includes a significant entropic effect. The high enthalpy barrier of the first chemical step is considerably compensated for by the positive entropic contribution. In contrast, the barrier of the second chemical step is dominantly formed by an unfavorable entropic contribution (Figure 4E).

### Determination of the Oligomeric State

The oligomeric states of ZenA_Re_ and ZenA_Scfl_ were determined by size exclusion chromatography and multiangle light scattering (SEC-MALS). For ZenA_Re_, the calculated molar mass of the 6xHis-tagged subunit is 37.103 g/mol (assuming cleavage of the *N*-terminal fMet), and the observed molar mass of 71 kDa (Figure S9A, Supporting Information) indicated the presence of a dimer. For ZenA_Scfl_, the calculated molar mass of the 6xHis-tagged monomer is 34.667 g/mol (again assuming cleavage of the *N*-terminal fMet), and the observed molar mass of 138 kDa (Figure S9B, Supporting Information) indicated the presence of a tetramer.

### Structural Analysis

ZenA_Scfl_ was chosen as the first crystallization target due to its greater temperature stability. Selenomethionine-labeled ZenA_Scfl_ (PDB ID: 8CLN) was prepared, crystallized in space group *P*3_1_2 1 with four molecules in the asymmetric unit, and used for phase determination exploiting a selenium anomalous signal with a dataset at 2.5 Å resolution (Table S3 and Figure S10, Supporting Information). The high-resolution ZenA_Scfl_ structure (1.4 Å resolution, PDB ID: 8CLO) was refined in the space group C 1 2 1 with two molecules in the asymmetric unit, with final R-work/R-free of 0.187/0.216, respectively (Table S3, Supporting Information).

**Figure 7:**
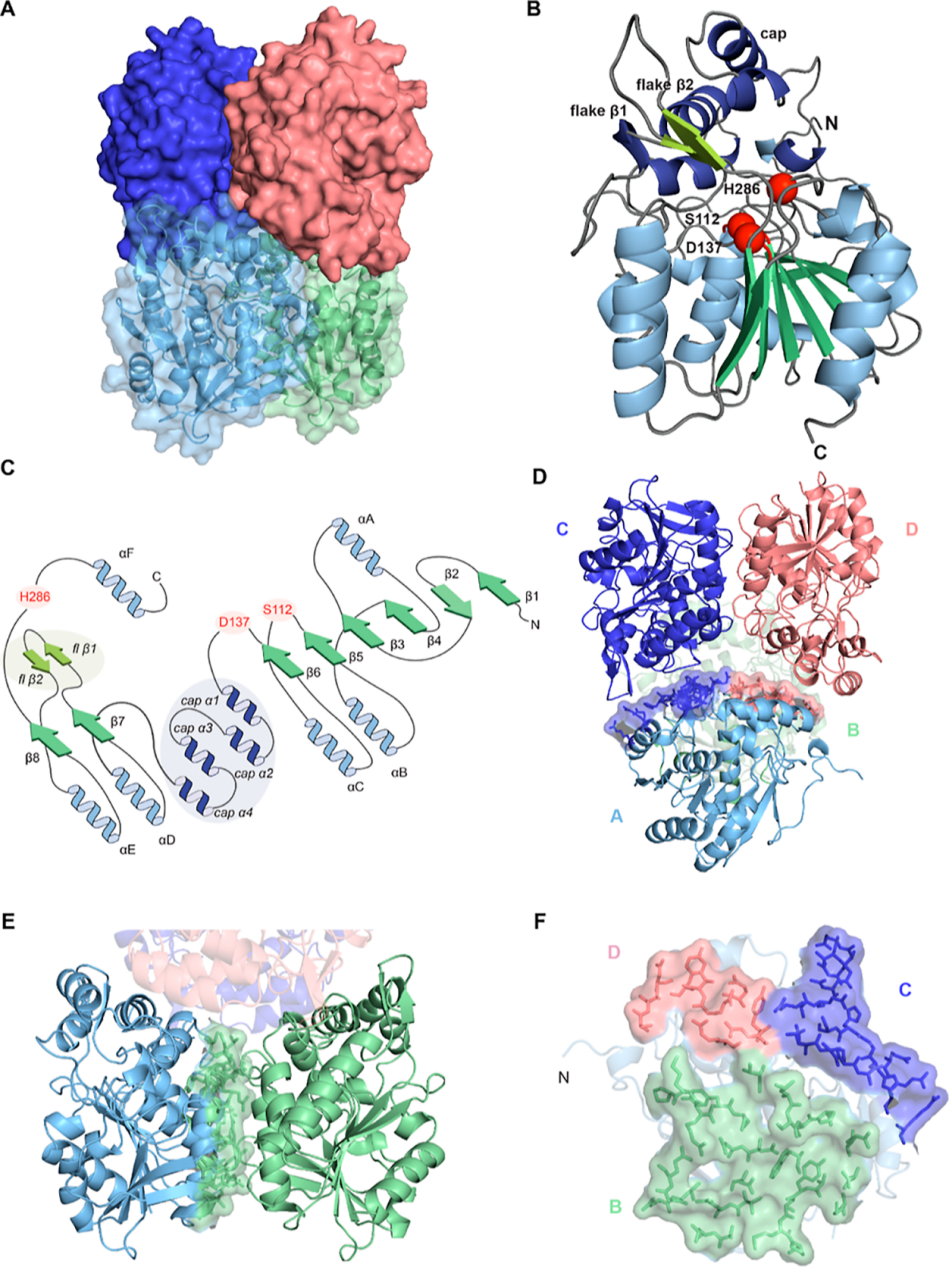
Structural overview of ZenA_Scfl_. (**A**) Structure of the ZenA_Scfl_ tetramer shown in surface representation. Chains A and B are displayed as a cartoon below the transparent surface. (**B**) ZenA_Scfl_ subunit (chain A) shown as cartoon, color coding according to topology as in **C**. Active site residues are highlighted as red spheres. (**C**) Topology diagram of ZenA, with secondary structure elements of the α/β hydrolase fold labeled according to Ollis et al.^90^ and shown in green (β-strands) and blue (α-helices). Secondary structures of the cap and the peripheral, antiparallel β-sheet (dubbed “flake”) are labeled separately and shown in pale green (β-strands) and marine (α-helices) and highlighted. (**D**) Tetramer interfaces between chains A and C (blue) and between chains A and D (light pink). (**E**) Dimeric interface between chains A (cyan) and B (green). (**F**) Detail of tetrameric and dimeric interfaces of chain A shown as sticks with surface representation, color coded according to the interacting chain. Chain A is shown as a transparent cartoon. PDB ID: 8CLO.

The ZenA_Scfl_ tetramer is a dimer of dimers (Figure 7A), and its subunits display canonical features of the α/β hydrolase fold (Figure 7B). The core domain features a central β-sheet made of eight strands, which are parallel except for strand β2, which is inverted. This twisted β-sheet is sandwiched between two α-helices (A and F) on the front side and four α-helices (B, C, D, and E) on the back side (Figure 7B,C). ZenA_Scfl_ features a cap domain inserted between sheet β6 and helix αD, composed of four α-helices. It is positioned on top of the catalytic triad and forms, together with the core domain, the active site (Figure 7B,C).

Notably, ZenA_Scfl_ features a β-sheet formed by two short, antiparallel strands linked by a tight turn between β-strand 7 and α-helix E, which protrudes from the α/β hydrolase core and touches the left side of the cap domain. None of the related structures returned by the DALI protein structure comparison server^96^ using the coordinates of ZenA_Scfl_ displayed such a structural feature (Figure 7B,C). We nicknamed this peculiar β-sheet, the “flake”.

The tetramer is composed of two stacked dimers (chains A and B; C and D), which are related by a 180° rotation through the crystallographic 2-fold axis. The dimeric interface is the same as reported for other α/β hydrolases,^97^ involving 30 residues interacting via 14 hydrogen bonds and 6 salt bridges over an interface surface of 807 Å^2^ (protein interfaces, surfaces, and assemblies’ service PISA at the European Bioinformatics Institute (http://www.ebi.ac.uk/pdbe/prot_int/pistart.html).^98^ The expected solvation free energy gain from interface formation amounts to Δ*G* = −6.1 kcal mol^–1^ with a *P*-value of 0.541, where a P value above 0.5 indicates that the interface is not as hydrophobic as it could be and could thus be an artifact (chains A/B, Δ*G* = −8.8 kcal mol^–1^, and calculated dissociation constant K_D_ = 0.34 μM at 25 °C, according to Prodigy,^99^Figure 7E,F, green).

Two inter subunit interactions are responsible for tetramer formation. One is between chains A and C (equivalent to B and D) and is mediated by 18 residues forming 8 hydrogen bonds over a surface area of 350 Å^2^ with Δ*G* = +1.5 kcal mol^–1^, with a *P*-value of 0.865 [chains A/C, Δ*G* = −8.9 kcal mol^–1^, K_D_ = 0.27 μM at 25 °C, according to Prodigy (Figure 7D,F, blue)]. The other interaction, between chains A and D (B and C), involves the peripheral antiparallel β-sheet (the “flake”) and is mediated by 12 residues over an interface area of 234 Å^2^ with Δ*G* = +1.9 kcal mol^–1^, with a *P*-value of 0.805 (chains A/D Δ*G* = −5.8 kcal mol^–1^, K_D_ = 54 μM at 25 °C, according to Prodigy, Figure 7D,F, light pink).

**Figure 8:**
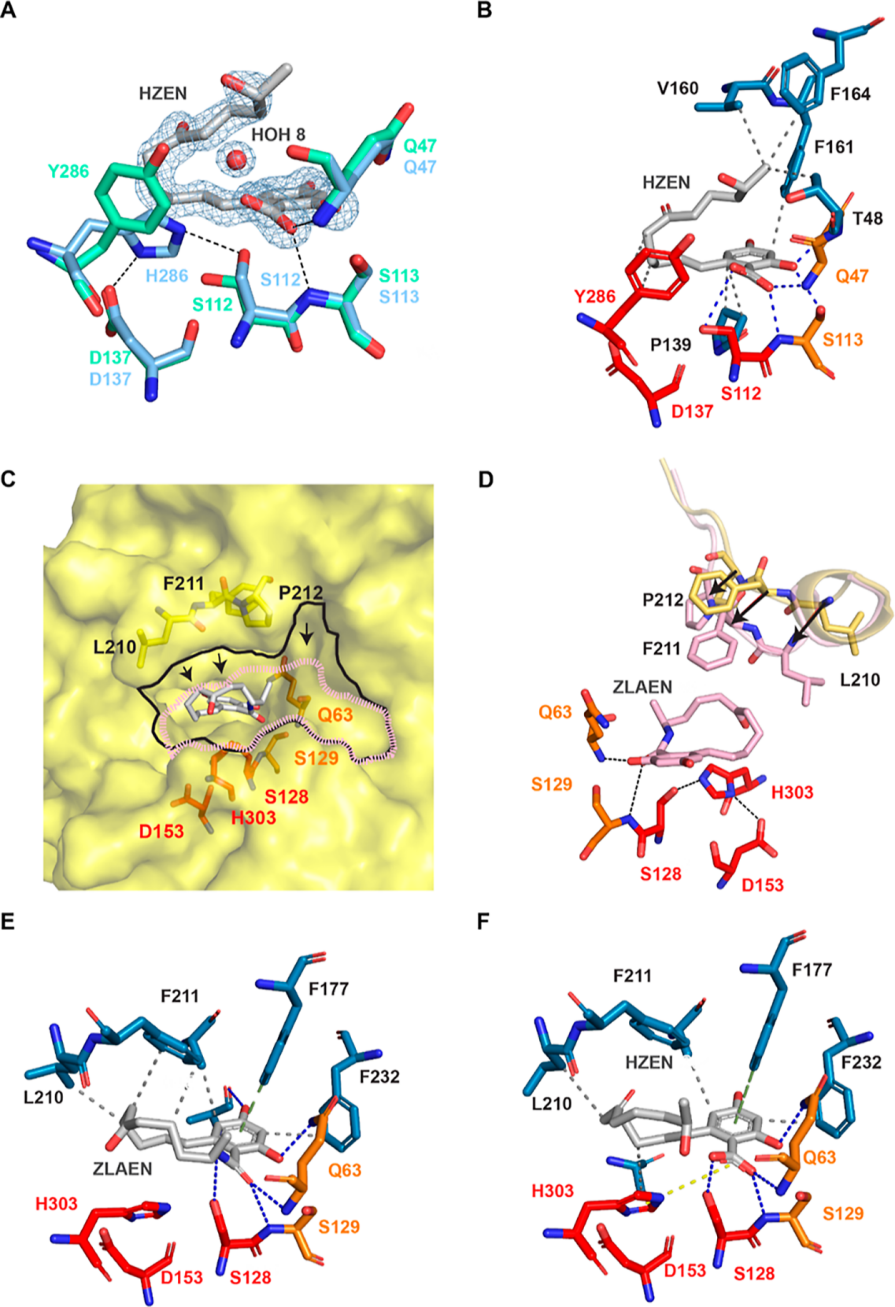
Ligand interactions of ZenA_Scfl_ and ZenA_Re_. (**A**) Alignment of catalytic triads of ZenA_Scfl_ (blue, PDB ID: 8CLO) and ZenA_Scfl_ H286Y in complex with HZEN (green, PDB ID: 8CLQ) shown as stick representation. Cleaved ZEN and water molecule HOH 8 from 8CLQ are shown as sticks and spheres, respectively. 2mFo-Fc contoured at 1.2 rmsd. (**B**) Stick representation of the ZenA_Scfl_ H286Y in complex with HZEN (PDB ID: 8CLQ): active site residues shown in red, oxyanion hole residues shown in orange, interacting residues shown in blue, and HZEN shown in gray. Hydrogen bonds are indicated via blue dashes and hydrophobic interactions are indicated via gray dashed lines. (**C**) Surface representation of the ZenA_Re_ apo structure (PDB ID: 8CLT). Active site opening of the apo structure is outlined in black. Superposed ligand ZLAEN (PDB ID: 8CLU) is shown as gray sticks. Active site opening with the bound ligand is highlighted as pink dashes. Closure of opening upon binding indicated by black arrows. (**D**) Active site (red) with oxyanion hole residues (orange) of ZenA_Re_ in complex with ZLAEN (pink, PDB ID: 8CLU) shown as sticks. Yellow sticks and cartoon representations show residues of the cap domain in apo conformation, pink sticks, and cartoons show cap domain residues in ligand-bound conformation. Black arrows indicate the displacement of Cα atoms. (**E**) Stick representation of ZenA_Re_ in complex with ZLAEN (gray, PDB ID: 8CLU): active site residues shown in red, oxyanion hole residues shown in orange, and interacting residues shown in blue. Hydrogen bonds are indicated via blue dashes, hydrophobic interactions are indicated via gray dashed lines, and the π-stacking interaction is shown as a green dashed line. (**F**) Stick representation of ZenA_Re_ in complex with HZEN (gray, PDB ID: 8CLV): active site residues shown in red, oxyanion hole residues shown in orange, and interacting residues shown in blue. Hydrogen bonds are indicated via blue dashes, hydrophobic interactions are indicated via gray dashed lines, the π-stacking interaction is shown as a green dashed line, and a salt bridge is indicated via yellow dashes.

The catalytic triad of ZenA_Scfl_ is located in negatively charged cavities opposite the dimer interface (Figure S11A,C, Supporting Information) and is composed of Ser-112, Asp-137, and His-286. The nucleophile Ser-112 is located at a sharp turn connecting β-strand 5 and α-helix C, termed “nucleophilic elbow” in α/β hydrolases^90^ (Figure 7B,C). The sequence of ZenA_Scfl_ contains the conserved glycine residues Gly-110 and Gly-114, which are required for the formation of the tight turn at the nucleophilic elbow (**Gly**-110/Asn-111/**Ser**-112/Ser-113/**Gly**-114). Ser-112 is found at a singular position in the Ramachandran plot, with dihedral angles φ = 63.3° and Ψ = −126.0° (chain A). The structure of ZenA_Scfl_ confirmed conserved His-286 in the loop between β-strand 8 and α-helix F as the catalytic histidine, at a distance of 2.9 Å between NE2 and OG of Ser-112 (Figure 8A). The crystal structure showed Asp-137 OD2 positioned at a distance of 2.7 Å from ND1 of the catalytic His-286.

In α/β hydrolases with a cap, the classical position of the catalytic triad’s acidic Asp residue is in a loop connecting β-strand 7 to α-helix E.^90,92^ However, in ZenA_Scfl_, the catalytic Asp-137 is located in a loop following β-strand 6 (Figure 7C), an alternate position for the acidic residue also found in other α/β hydrolases.^100−102^ In the enzyme chloroperoxidase L from *Streptomyces lividans* (CPO-L, PDB ID: 1A88),^103^ which was returned by the DALI server with the highest Z-score when searched with the coordinates of ZenA_Scfl_, the catalytic aspartic acid is at the canonical position after β-strand 7. After superpositioning chain A of ZenA_Scfl_ and CPO-L, the Cα atoms of the catalytic aspartic acids D137 and D226 are 7.9 Å apart, whereas the CG atoms and OD2 atoms are significantly closer (3.4 Å distance, 1.4 Å distance, respectively, Figure S12, Supporting Information). The hydrogen bonding distance of the aspartic acid OD2 to the ND1 of the corresponding catalytic histidine is identical for CPO-L and ZenA_Scfl_ with 2.66 and 2.65 Å, respectively. However, in CPO-L, the hydrogen bond of Asp-226 is in the *syn* conformation, on the same side as the other oxygen atom of the carboxylate group, whereas the hydrogen bond formed by Asp-137 in ZenA_Scfl_ is in the *anti* conformation, directed away from the second oxygen atom. Notably, Asp-137 adopts a left-handed α-helix conformation in the Ramachandran plot. The backbone amides NH of Ser-113 and Gln-47 are in positions that suggest their functional role as hydrogen bond donors of the oxyanion hole of ZenA_Scfl_.

Of the site-directed mutants with a disrupted catalytic triad, ZenA_Scfl_ H286Y was used for the structural investigation of enzyme–substrate interactions. ZenA_Scfl_ H286Y crystallized in the presence (PDB ID: 8CLQ) as well as in the absence (PDB ID: 8CLP) of 5 mM ZEN in P 2_1_ 2_1_ 2_1_ with 4 subunits in the asymmetric unit and a solvent content of 39.5%. The resolutions for ligand bound and unbound structures were 1.53 and 1.92 Å, respectively. The structure of ZenA_Scfl_ H286Y was highly conserved compared to the ZenA_Scfl_ wild-type, with a RMSD of 0.31 Å (over 270 equiv C_α_ atoms), except that the loop Ala-188 to Met-191 between cap α-helices 2 and 3 was not resolved. Tyr-286 remained approximately in the ring plane of His-286 but was slanted away from the catalytic Ser-112 by about 80°.

For ZenA_Scfl_ H286Y cocrystallized with ZEN, refinement was initially carried out with substrate ZEN modeled in the active site. However, the difference density map and a subsequently calculated Polder map revealed a better fit of product HZEN in the active site (Figure S13, Supporting Information). The β-resorcylic acid moiety of HZEN was found in a well-defined position at the active site, while the open-chain hydroxyketone moiety appeared flexible. The aromatic side chain of Tyr-286 was positioned as in ZenAScfl H286Y without ligand, but OG of Ser-112 was rotated to occupy the created vacancy (Figure 8A). The distance between the atoms that would be forming a covalent bond as a reaction intermediate in wild-type ZenA_Scfl_, carboxylate C12 of HZEN, and OG of Ser-112, is 3.8 Å. However, this distance is only 2.1 Å when measured from the OG of naturally positioned Ser-112 of aligned wild-type ZenA_Scfl_, which therefore appears reaction-competent. The side-chain amide group of Gln-47 as well as OG of Ser-113 form hydrogen bonds with the hydroxyl moiety bound to C2 of HZEN (2.8 and 3.2 Å, respectively) (Figure 8B). Nonpolar interactions with HZEN are formed by the residues Pro-139, Val-160, Phe-161, and Phe-164. Conserved Pro-139 is at the base of the loop connecting β-strand 6 to cap α-helix 1. Phe-161 from the cap α-helix 1 is also conserved (Figure 2).

Despite its lower thermostability, ZenA_Re_ could also be crystallized (PDB ID: 8CLT). Crystals of space group C 2 2 2_1_ with 2 subunits in the asymmetric unit and 46% solvent content, yielded a dataset of 1.46 Å resolution. The structure was solved by molecular replacement, with ZenA_Scfl_ as a search model. The structures of ZenA_Re_ and ZenA_Scfl_ were found to be very similar, with a RMSD of 0.43 Å (over 502 equiv Cα atoms) (Figure S14A,C, Supporting Information), with the catalytic triad and oxyanion hole residues superimposing almost perfectly (Figure S14B, Supporting Information). Most differences between the structures are related to the cap domains. Similar to ZenA_Scfl_, ZenA_Re_ shows mostly negative electrostatic surface potential, especially at the active site (Figure S11B,D, Supporting Information). While ZenA_Re_ has a similar dimeric interface (Figure S15A,B, and C, Supporting Information), composed of 30 residues over an interface area of 1195 Å^2^ (Δ*G* = −9.4 kcal mol^–1^, K_D_ = 0.13 μM at 25 °C, according to Prodigy^99^), six amino acids are different in the region corresponding to the tetramer forming interfaces between subunits A/C of ZenA_Scfl_ and five amino acids are different in the interface region between the subunits A/D (Figure S15D, Supporting Information). These differences lead to the abrogation of tetramer stabilizing interactions and corroborate the SEC-MALS result, which showed that ZenA_Re_ is a dimer in solution (Figure S9A, Supporting Information).

As inactivation of ZenA_Scfl_ by substituting the catalytic His-286 with tyrosine (H286Y) caused conformational changes at the active site, we attempted to get structural information with a bound, noncleavable substrate analogue. A derivative of ZEN, which is a cyclic amide rather than a cyclic carboxylic ester, was synthesized (Figure S16, Supporting Information). In line with proposed nomenclature guidelines,^64^ we called the molecule zearalactamenone (ZLAEN). ZLAEN was verified to be a poor substrate, and enzymatic hydrolysis was only detectable after prolonged incubation with a very high ZenA concentration. ZLAEN was soaked into crystals of ZenA_Re_, yielding a dataset of 1.8 Å resolution (PDB ID: 8CLU). The ligand was found at the active site of ZenA_Re_, and the resorcylic acid moiety was in the same position as the one of HZEN in ZenA_Scfl_ H286Y. Most of the structure of ZenA_Re_ remained unchanged upon binding of ZLAEN. The residues of the catalytic triad and oxyanion hole did not change position, and the overall RMSD was 0.08 Å (over 253 equiv C_α_ atoms). However, the positions of the amino acids Met-208 – Pro-214 at the lip of the cap were shifted upon binding. The biggest movements were observed for Leu-210, where C_α_ was shifted by 4.4 Å and CD by 6.7 Å, and Phe-211, where the C_α_ was shifted by 3.1 Å, and side-chain atoms were shifted by up to 5.8 Å (Figure 8C,D). The N of oxyanion hole residue I (Ser-113) and the amide N of oxyanion hole residue II were at a distance of 3.6 and 3.0 Å from O20 of ZLAEN. The nucleophilic Ser-112 appeared in the same orientation as in the unbound structure, forming a hydrogen bond with N21 of ZLAEN at a distance of 3.5 Å. The side-chain amide of Gln-63 formed a hydrogen bond of 2.8 Å with the hydroxyl group connected to C-16 of ZLAEN. Residues from the cap domain (Leu-210, Phe-211, Phe-177, and Phe-232) participated in hydrophobic interactions with the bound ligand, with Phe-177 engaging in a π–π stacking interaction with the β-resorcylic acid moiety of HZEN (Figure 8E).

As HZEN was found bound at the active site of ZenA_Scfl_ H286Y after cocrystallization with ZEN, cocrystallization of wild-type, active ZenA_Re_ with ZEN was also attempted. The obtained crystals diffracted up to 2.5 Å resolution (PDB ID: 8CLV). The RMSD compared with the structure without ligand was 0.147 Å (over 251 Cα), and compared with the structure with bound ZLAEN, it was 0.131 Å (over 276 Cα). The structure showed again that the position of the resorcylic acid moiety was well-defined. The lactone ester bond was cleaved, and the aliphatic hydroxy ketone moiety appeared flexible. Residues interacting with the bound ligand were positioned as in the closed conformation of ZenA_Re_ with bound ZLAEN (Figure S17, Supporting Information). The carboxylate atom O12 was at a distance of 3.1 and 3.1 Å from the N of oxyanion hole residue I and the amide N of oxyanion hole residue II, respectively. The nucleophilic serine Ser-112 appeared in the same orientation as in the structure with ZLAEN bound, with its OG at a distance of 2.7 Å from C12 of the carboxylic acid group. Nonpolar interactions with HZEN were again formed by the residues from the cap domain, with a π–π stacking interaction mediated by Phe-177. Additionally, the catalytic His-303 showed involvement in a weak electrostatic interaction with C12 of the carboxylic acid group of HZEN at 5.1 Å distance (Figure 8F).

## Discussion

*R. erythropolis* strains are known for their extraordinary ability to break down a variety of noxious molecules of natural or synthetic origin,^84^ and the hydrolysis of ZEN with ZenA is yet another example of their catabolic versatility. The reported protocol for genetic transformation of *R. erythropolis* with plasmid libraries with little bias toward plasmids with small or no inserts may therefore be useful in further work on biodegradation enzymes and pathways of *R. erythropolis*. ZEN is also not the only mycotoxin converted by *R. erythropolis*, and degradation of aflatoxin^104^ and ergopeptines^105^ has been reported. Aflatoxin degradation was also tested and confirmed for *R. erythropolis* PFA D8–1 (data not shown), and the genomic library might also lend itself to attempts at cloning genes for the catabolism of aflatoxin. The location of the *zenA* gene on the 660 kb linear megaplasmid pSFRL1 of *R. erythropolis* PFA D8–1 explains why other tested strains of *R. erythropolis* showed no hydrolysis of ZEN. Linear megaplasmids and the location of genes for xenobiotic catabolism on such plasmids are common in *R. erythropolis*.^82,84^ As all daughter colonies derived in the absence of selection pressure showed ZEN hydrolyzing activity, the plasmid pSFRL1 seems to be stably maintained. ZEN is one of several structurally related resorcylic acid lactones (RALs) with biological inhibition activity produced by various fungi,^106^ and may be a native substrate that drove the evolution of ZenA or a non-native, acceptable substrate for a promiscuous enzyme. *R. erythropolis* is a soil-borne bacterial species, and *Fusarium*-derived ZEN is often naturally present in soil.^107^ Our finding that the ZEN hydrolyzing activity of *R. erythropolis* PFA D8–1 was inducible with ZEN could indicate that ZEN is a native substrate. The strain was able to grow with ZEN as a substrate, although further catabolism of HZEN and DHZEN was slow, suggesting that ZEN may not be a prime source of carbon or energy. There is no evidence that ZEN could have a toxic or inhibiting effect on *R. erythropolis*. As our structural analysis showed that the resorcylic acid moiety but not the lactone ring moiety of ZEN was tightly bound, ZenA may have evolved for a RAL other than ZEN or for activity with more than one RAL. An interesting follow up on the present study could be to measure the hydrolytic activity of ZenA homologues for a spectrum of RALs.

Homologues of ZenA were mostly found in *Streptomyces* species and other bacteria belonging, as *Rhodococcus*, to the Gram-positive *Actinomycetia* with large genomes and high GC content. A typical habitat of *R. erythropolis* and *Streptomyces* species is nutrient-rich soil, where competition is not only with other bacteria but also with fungi. *Streptomyces* species are known for their production of antibiotics but also for antifungal molecules, and the ability to break down ZEN and possibly other fungal RALs may be an advantage in their biological niche. However, the biological significance of bacterial ZEN degradation remains speculative, while for fungal ZEN hydrolysis by the lactonase ZHD101 of *C. rosea*, it is clear that detoxification of ZEN plays a role in the mycoparasitic fungal lifestyle.^8^ Reported kinetic parameters for ZEN hydrolysis by ZHD101 are *K*_M_ = 5.1 ± 1.5 μM and *k*_cat_ = 0.173 ± 0.011 s^–1^ at pH 7.5,^53^ and interestingly, some of the bacterial ZEN lactonases tested here showed considerably higher activity at low ZEN concentrations. As we detected ZEN hydrolysis in several of our mixed microbial cultures, bacterial ZEN lactonase activity may not be uncommon.

The ZenA sequences and structures described here have uncommon or unique features, which distinguish them from other α/β hydrolases. These features include a peripheral, antiparallel β-sheet, which touches the cap on top of the α/β hydrolase. This feature, nicknamed “flake”, is involved in subunit interactions of the ZenA_Scfl_ tetramer. However, its significance for dimeric ZenA_Re_ is less clear. It may also serve the purpose of positioning the conserved glycine and alanine residues, located after the second β-strand, for interaction with the substrate. Interaction with the cap could be another function, although this interaction seems rather loose and caps of α/β-hydrolases are not normally stabilized.

The structural organization of the oxyanion hole of ZenA also diverges from the typical organization in α/β-hydrolases, in which the side chains of the motif play a role in anchoring the oxyanion hole. In ZenA, the conserved oxyanion II residue glutamine (Gln-63 of ZenA_Re_ and Gln-47 of ZenA_Scfl_, Figure 2) seems to contribute to substrate binding instead. An anchoring role may be contributed by the preceding glutamic acid residue (Glu-62 of ZenA_Re_ and Glu-46 of ZenA_Scfl_), which forms a hydrogen bond with a serine residue in helix A (Ser-69 of ZenA_Re_ and Ser-53 of ZenA_Scfl_), although these residues are not strictly conserved. Interestingly, another α/β hydrolase without glycine before the oxyanion II residue, PcaD (PDB ID: 2XUA), also catalyzes the hydrolysis of a lactone ester.^108^

Both the bacterial ZEN lactonase, ZenA, and the fungal ZEN lactonase, ZHD101, have the acidic residue of the catalytic triad positioned after β-strand 6 rather than β-strand 7, as in the majority of enzymes with the α/β hydrolase fold. This position of the acidic residue has been known for a long time,^109^ is functionally equivalent,^100,110^ and enzyme variants with swapped position of the acidic residue have been engineered successfully.^111,112^ For ZenA, this functional equivalence is evident from the alignment with CPO-L. The *anti* conformation of the hydrogen bond formed by the carboxylic acid in ZenA may have a weaker, compared with the *syn* conformation, proton pull from the catalytic histidine as a consequence,^113^ but the difference is probably small,^114^ and catalytically crucial hydrogen bonds in *anti* conformation are found in many other enzymes.

The tetrameric state of ZenA_Scfl_ may contribute to its higher thermostability compared with dimeric ZenA_Re_, as biomolecular interactions at oligomeric interfaces can greatly affect thermostability.^115,116^

The sequence identity between bacterial and fungal ZEN lactonases is in the range of only 13 to 18% (Figure S18, Supporting Information). Nevertheless, bacterial and fungal ZEN lactonases share some common features: both have the acidic residue of the catalytic triad positioned in the loop after β-strand 6 rather than 7. Both have an additional serine residue following the catalytic serine, and similar interactions form between this serine’s side chain and ZEN’s carboxyl and hydroxyl groups. Both diverge from the canonical G**X** or GG**G**X types of the oxyanion hole, although only bacterial ZEN lactonases exhibit direct interaction of the side chain of the oxyanion hole II residue with substrate ZEN. An interesting difference is that while ZenA provides substrate access to the active site under the front of the cap, perpendicular to the central β-sheet, ZHD101 offers substrate access under the side of the cap (Figure S19, Supporting Information). In ZHD101, this access path is at the back side of the central β-sheet and almost parallel to it,^54^ while in ZenA, such a path to the active site is blocked off by the “flake”. The relative orientations of the resorcylic acid moiety of ZEN and catalytic triad residues are similar. It would be interesting to deduce from sequence analysis if a common ancestor of bacterial ZenA and fungal ZHD had ZEN hydrolyzing activity or if this activity developed by convergent evolution.

Presteady-state kinetic analysis revealed a step in the reaction mechanism between substrate binding and chemical intermediate formation, assigned as “enzyme closure”. However, crystal structure analysis revealed that only ZenA_Re_ underwent a structural change resembling enzyme closure, with residues in the cap’s lip adopting a more closed conformation of the ZEN-binding pocket. Due to the apparent absence of this movement in ZenA_Scfl_, the ″enzyme closure″ phase may also involve a rearrangement of the lactone ester ring to the competent conformation in the ZEN-binding site. At 8 °C, where we have presteady-state data for both enzymes, the rate constant *k*_2_ (enzyme closing) was lower for ZenA_Re_ than for ZenA_Scfl_ (Table S1, Supporting Information). This velocity difference may be caused by the protein dynamics displayed by ZenA_Re_ but not ZenA_Scfl_. Similar to ZenA_Scfl_, ZHD101 demonstrates no motion upon binding of ZEN.^54^

A potential application other than gastrointestinal detoxification of ZEN with a recombinant feed enzyme^50^ could be to express *zenA* in genetically modified crop plants for *in planta* hydrolytic degradation of ZEN. However, ZEN degradation may not convey much resistance against *Fusarium* infection, and harvested cereal grain could still be contaminated with deoxynivalenol.

## Conclusions

We isolated a *R. erythropolis* strain with ZEN hydrolyzing activity and cloned its ZEN lactonase gene *zenA*. Homologous enzymes from other bacteria also catalyzed the hydrolysis of ZEN, and we performed comparative biochemical characterization. Crystal structures of dimeric ZenA_Re_ and more thermostable, tetrameric ZenA_Scfl_ with and without bound substrate or substrate analogue ZLAEN confirmed the predicted α/β hydrolase fold and suggested a canonical hydrolytic reaction mechanism based on a Ser-His-Asp catalytic triad and an oxyanion hole. Unusual structural features included a peripheral, antiparallel β-sheet (“flake”) connecting β-strand 7 with α-helix E, and a noncanonical oxyanion hole motif. Transient kinetics at 8 to 19 °C revealed that for ZenA_Re_, structural dynamics were rate-limiting at low temperatures, where reaction rates were strongly promoted by increasing temperature until reaction chemistry became rate-limiting. According to the measured kinetic parameters, some bacterial ZenAs hydrolyze ZEN faster than the previously reported fungal ZEN lactonases. ZenA may present a promising foundation for developing enzyme technology aimed at degrading the mycotoxin ZEN, a common contaminant in feed and food. The reported enzyme structures and transient kinetics offer a robust platform for advancing enzyme engineering.

## Supporting Information Available

Time curves of ZEN hydrolysis by *R. erythropolis* strains; time curves of ZEN hydrolysis by lysates of induced and noninduced *R. erythropolis* PFA D8–1; pulsed field agarose electrophoresis gel showing the linear megaplasmid pSFRL1 of *R. erythropolis* PFA D8–1; Michaelis–Menten plot for steady-state ZEN hydrolysis by ZenA_Re_; kinetic parameters for ZenA_Re_ and ZenA_Scfl_ obtained by analytical fitting; stopped-flow fluorescence traces for ZEN, ZenA_Scfl_, and ZenA_Scfl_ + ZEN; kinetic and thermodynamic parameters obtained by numerical integration and global data fitting; confidence contour analysis of ZenA_Re_ kinetic and thermodynamic parameters; conventional fitting of ZenA_Scfl_ kinetic data; confidence contour analysis of ZenA_Scfl_ kinetic parameters; SEC-MALS results for ZenA_Re_ and ZenA_Scfl_; data collection and refinement statistics from X-ray crystallography; structure of SeMet derivative of ZenA_Scfl_; electrostatic surface maps of ZenA_Scfl_ and ZenA_Re_; structural alignment of catalytic triads of ZenA_Scfl_ and chloroperoxidase L (CPO-L); electron density and electron density difference maps for ZenA_Scfl_ H286Y with bound ligand; structural alignment of ZenA_Scfl_ and ZenA_Re_; oligomerization interfaces of ZenA_Re_; structure of ZLAEN; comparison of ZenA_Re_ structures with and without bound ligands; sequence alignment of bacterial and fungal ZEN lactonases; and comparison of substrate access paths of ZenA and ZHD101 (PDF)

## Abbreviations

ZEN: zearalenone ((*S*,*E*)-14,16-dihydroxy-3-methyl-3,4,5,6,9,10-hexahydro-1*H*-benzo[c][1]oxacyclotetradecine-1,7(8*H*)-dione)
HZEN: hydrolyzed zearalenone ((*S*,*E*)-2,4-dihydroxy-6-(10-hydroxy-6-oxoundec-1-en-1-yl)benzoic acid)
DHZEN: decarboxylated hydrolyzed zearalenone ((*S*,*E*)-1-(3,5-dihydroxyphenyl)-10-hydroxyundec-1-en-6-one)
ZLAEN: zearalactamenone ((S,E)-14,16-dihydroxy-3-methyl-3,4,5,6,9,10-hexahydrobenzo[c][1]azacyclotetradecine-1,7(2H,8H)-dione)
ZEL: zearalenol
DAD: diode array detector
ER: estrogen receptor
BSA: bovine serum albumin
SEC-MALS: size-exclusion chromatography–multiangle light scattering
TS: Teorell−Stenhagen
RAL: resorcylic acid lactone
SeMet: selenomethionine
