# Supplementary material for: Bacterial Lactonases ZenA with Noncanonical Structural Features Hydrolyze the Mycotoxin Zearalenone
Source: ACS Catal. 2024 Feb 16;14(5):3392–410. doi: 10.1021/acscatal.4c00271 (PMC10913051; doi:10.1021/acscatal.4c00271)
Supplement: Supplementary file 1 — cs4c00271_si_001.pdf [file cs4c00271_si_001.pdf]

Supporting Information

for

Bacterial lactonases ZenA with noncanonical structural features hydrolyze the mycotoxin zearalenone

Sebastian Fruhauf[1], Dominic Pühringer[2], Michaela Thamhesl[1], Patricia Fajtl[1], Elisavet Kunz-Vekiru[3α], Andreas Höbartner-Gussl[1], Gerd Schatzmayr[1], Gerhard Adam[4], Jiri Damborsky[5,6], Kristina Djinovic-Carugo[2,7,8], Zbynek Prokop[5,6] and Wulf-Dieter Moll[1]**

[1]dsm-firmenich Animal Nutrition and Health R&D Center Tulln, Technopark 1, 3430 Tulln, Austria; [2]Department for Structural and Computational Biology, Max Perutz Laboratories, University of Vienna, Campus Vienna Biocenter 5, 1030 Vienna, Austria; [3]Institute of Bioanalytics and Agro-Metabolomics, Department of Agrobiotechnology IFA-Tulln, University of Natural Resources and Life Sciences Vienna (BOKU), Konrad-Lorenz-Straße 20, 3430 Tulln, Austria; [4]Institute of Microbial Genetics, Department of Applied Genetics and Cell Biology, University of Natural Resources and Life Sciences Vienna (BOKU), Konrad-Lorenz-Straße 24, 3430 Tulln, Austria; [5]Loschmidt Laboratories, Department of Experimental Biology and RECETOX, Faculty of Science, Masaryk University, Kamenice 5, Bld. A13, 625 00 Brno, Czech Republic; [6]International Clinical Research Center, St. Anne's University Hospital Brno, Pekarska 53, 656 91 Brno, Czech Republic; [7]Department of Biochemistry, Faculty of Chemistry and Chemical Technology, University of Ljubljana, Ljubljana, Slovenia; [8]European Molecular Biology Laboratory (EMBL) Grenoble, Grenoble, France

Contents

Materials and Methods 4

Isolation of *R. erythropolis* PFA D8-1 4

Characterization of *R. erythropolis* PFA D8-1 5

Cloning of the *zenA* gene 7

Characterization of $ZenA_{Re}$ 9

Characterization of ZenA homologues 12

HPLC-DAD and LC-MS/MS quantification of ZEN and derivates 13

Pre-steady-state kinetic measurements 14

Kinetic Data Analysis and Statistics 15

SEC-MALS 20

Site-directed mutagenesis 21

Production of selenomethionine-substituted $ZenA_{Scfl}$ 21

Crystallization 23

Structure determination 23

References 24

Figure S1. Hydrolysis of ZEN by *R. erythropolis* strains. 27

Figure S2. Hydrolysis of ZEN by clear lysate of *R. erythropolis* PFA D8-1 28

Figure S3. Pulsed field agarose gel with DNA of *R. erythropolis* PFA D8-1. 29

Figure S4. Steady-state ZEN hydrolysis velocity of $ZenA_{Re}$ at various ZEN concentrations. 30

Table S1. The initial values of the kinetic parameters. 31

Figure S5. Stopped-flow fluorescence intensity readings for ZEN, $ZenA_{Scfl}$, and $ZenA_{Scfl}$ + ZEN. 32

Table S2. The kinetic and thermodynamic parameters obtained by global fit. 33

Figure S6. Confidence contour analysis of $ZenA_{Re}$ kinetic and thermodynamic parameters. 34

Figure S7. Conventional fitting of $ZenA_{Scfl}$ kinetic data. 35

Figure S8. Confidence contour analysis of $ZenA_{Scfl}$ kinetic parameters. 37

Figure S9. SEC-MALS analysis of oligomeric state of $ZenA_{Re}$ and $ZenA_{Scfl}$. 38
Table S3. Data collection and refinement statistics for ZenA structures. 39
Figure S10. SeMet derivative structure of $ZenA_{Scfl}$ (PDB ID 8CLN). 41
Figure S11. Electrostatic surfaces of $ZenA_{Scfl}$ and $ZenA_{Re}$. 42
Figure S12. Structural alignment of catalytic triads of $ZenA_{Scfl}$ and CPO-L. 43
Figure S13. Ligand identification with $ZenA_{Scfl}$ H286Y (PDB ID: 8CLQ) dataset. 44
Figure S14. Structural alignment of $ZenA_{Scfl}$ and $ZenA_{Re}$. 45
Figure S15. Oligomerization interfaces of $ZenA_{Re}$. 46
Figure S16. Structure of zearalactamenone. 47
Figure S17. Comparison of apo and ligand bound structures of $ZenA_{Re}$. 48
Figure S18. Alignment of bacterial ZenA with fungal ZHD ZEN lactonases. 50
Figure S19. Comparison of substrate access paths to active sites of $ZenA_{Re}$ and ZHD101. 51

Materials and Methods

Isolation of R. erythropolis PFA D8-1

Soil samples were collected from maize fields in Lower Austria. Portions of 2 g of soil were resuspended in 20 ml of Brunner mineral medium (DSMZ medium 457, https://www.dsmz.de/microorganisms/medium/pdf/DSMZ_Medium457.pdf) with added vitamins: 20 µg/l biotin, 20 µg/l folic acid, 100 µg/l pyridoxine hydrochloride, 50 µg/l thiamine hydrochloride, 50 µg/l riboflavin, 50 µg/l nicotinamide, 50 µg/l calcium pantothenate, 1 µg/l cyanocobalamin, 1 mg/l menadione, 220 µg/l vitamin K_1, 50 µg/l p-aminobenzoic acid, 50 µg/l lipoic acid. Particles were removed by paper filtration, and 100 µl of filtered resuspensions were used as inoculum for enrichment cultures of 5 ml of Brunner mineral medium with vitamins and 50 µg/ml ZEN (Romer Labs), a concentration that exceeds the limit of solubility in aqueous buffer [1]. The cultures were incubated at 22°C with shaking (250 rpm) for three weeks, a resuspension of ZEN in water was used to supply ZEN to a nominal additional concentration of 50 µg/ml ZEN twice a week, and samples were taken and analyzed for ZEN concentration after every week. Evaporation was compensated for with water. To isolate ZEN-degrading strains that can be cultivated and cryo-conserved, samples which showed consumption of ZEN were cryo-conserved with 10% glycerol, and aliquots of such frozen samples were plated on Brunner mineral medium with vitamins, 1.5% bacteriological agar (Oxoid), and 50 µg/ml ZEN. Biomass from such plates was used to inoculate nutrient broth (CM0001, Oxoid) in deep-well microtiter plates (25°C, 800 rpm). Parts of the cultures were cryo-conserved at -80°C with 10% glycerol, and the remaining biomass was sedimented, resuspended in Brunner mineral medium with vitamins and 2 µg/ml ZEN, incubated (120 h, 25°C, 800 rpm), and ZEN concentrations were measured. Cryo-cultures from ZEN-conversion positive wells were streaked for single colonies on nutrient agar (CM0003,

Oxoid), and cultivation and ZEN conversion testing in deep-well microtiter plates were repeated as above with single daughter colonies as inoculum. Positive cultures were cryo-conserved again, and the cycle of streaking for single colonies on nutrient agar, cultivation in nutrient broth, cryo-conservation and testing for ZEN-conversion activity with 2 µg/ml ZEN in Brunner mineral medium with vitamins was repeated two more times, until strains were considered stable, active and pure.

Genomic DNA was isolated with QIAGEN genomic DNA buffers and tips, and 16S rDNA was amplified by PCR with primers 27f (5'-AGAGTTTGATCMTGGCTCAG-3') and 1492r (5'-TACGGYTACCTTGTTACGACTT-3') [2] and sequenced with primers 609f (5'-GGATTAGATACCCBDGTA-3') and 907r (5'-CCGTCAATTCMTTTGAGTTT-3'). Whole-genome sequencing of a combination of a shotgun and a 3 kb paired-end library was performed by LGC Genomics GmbH using Roche 454 GS FLX Titanium sequencing technology. The RAST server (https://rast.nmpdr.org/rast.cgi) [3] was used for sequence annotation and analysis.

Characterization of R. erythropolis PFA D8-1

R. erythropolis PFA D8-1 was deposited at the German Collection of Microorganisms and Cell Cultures DSMZ with accession number DSM 27240.

Hydrolysis of ZEN with biomass of *R. erythropolis*: Strains DSM 43066^T and PFA D8-1 were cultivated to stationary phase in nutrient broth at 25°C and 200 rpm for 20 h. Biomass was harvested by centrifugation and resuspended in the same volume of Brunner mineral medium with added vitamins. ZEN was added from a stock solution with 40% acetonitrile to a final concentration of 10 µg/ml (final acetonitrile concentration: 0.76%), and the cultures were

incubated at 25°C and 200 rpm. Samples were taken at time points and inactivated by heating to 95°C for 5 min, centrifuged, and subjected to LC-MS/MS analysis.

Hydrolysis of ZEN with lysate of *R. erythropolis* PFA D8-1: Two cultures were grown to stationary phase in nutrient broth at 25°C and 200 rpm, one with 50 µg/ml ZEN added as crystals, and one without. Biomass was harvested by centrifugation, resuspended in the same volume of Brunner mineral medium with vitamins, and lysed by passing 3x through a French pressure cell at 30 000 psi. The lysate was cleared by centrifugation and passed through a 0.45 µm filter and then a 0.20 µm filter. ZEN was added (final concentrations: 2 µg/ml ZEN, 0.14% acetonitrile), the cultures were incubated at 25°C and 200 rpm, and time point samples were taken, inactivated (5 min 95°C), centrifuged, diluted to the double volume with 20% acetonitrile in water, and subjected to LC-MS/MS analysis.

For pulsed-field gel electrophoresis, *R. erythropolis* PFA D8-1 was grown in nutrient broth at 25°C and 200 rpm for 48 h. Biomass from 3.5 ml culture was sedimented, washed twice with 1 ml PIV solution (10 mM Tris-HCl, 1 M NaCl, pH 7.6) at 4°C, and resuspended in 0.5 ml PIV solution. Agarose plugs were made by adding 150 µl cell resuspension with lysozyme (10 mg/ml) and mutanolysin (40 u/ml) to 850 µl melted 1% agarose (Bio-Rad) and transferring to plug moulds. Solidified plugs were immersed in 10 ml lysis solution (6 mM Tris-HCl pH 7.6, 1 M NaCl, 100 mM EDTA, 0.5% Brij 58, 0.2% sodium deoxycholate, 0.5% N-lauroylsarcosine sodium salt, 0.06 mg/ml RNAse A, 10 mg/ml lysozyme, 40 U/ml mutanolysin) and incubated at 37°C for 16 h. Plugs were washed with ES buffer (0.5 M EDTA, 1 % N-lauroylsarcosine sodium salt), incubated with 1 mg/ml proteinase K in the same buffer at 50°C for 16 h, washed three times for 45 min with TE buffer (10 mM Tris-HCl, 1 mM EDTA, pH 7.5), and inserted in a 1.1% agarose gel prepared with 0.5 x TBE-buffer (5.4 g/l Tris base, 2.75 g /l boric acid, 2 mM EDTA, pH 8.0). Yeast chromosomal

DNA (Bio-Rad) was used as a molecular size marker. The gel was run using CHEF DR III equipment (BIO-Rad) at 5.2 V/cm and 120° angle for 24 h at 14°C with 25 s initial switch time and 125 s final switch time. The gel was stained with GelRed 3X in water (Biotium) and recorded on a UV table.

Cloning of the zenA gene

The strategy for cloning of a ZEN detoxification gene was to generate a gene library with DNA of ZEN degrading *R. erythropolis* PFA D8-1 in a shuttle vector, to transform it into a strain without ZEN degradation ability, and to screen for transformants with gained ZEN degradation function. Genomic DNA of *R. erythropolis* PFA D8-1 was isolated with a QIAGEN Genomic-tip 500/G kit. DNA was partially digested with Hin1II and separated by electrophoresis on a 0.8% agarose gel. A gel slice with DNA fragments > 8 kbp was cut out, and the Promega Wizard SV gel and PCR clean-up kit was used to isolate the DNA. The *E. coli – Rhodococcus* shuttle vector plasmid pMVS301 [4] was propagated in *E. coli* HB101, isolated with a QIAGEN plasmid midi-kit, digested with restriction enzyme SphI and dephosphorylated with antarctic phosphatase, applied to 0.8% agarose gel electrophoresis, and purified with the Promega gel and PCR clean-up kit. Digested genomic DNA and pMVS301 were ligated with T4 DNA ligase, and the ligation reaction was transformed into *E. coli* DH10B by electroporation. Colonies grown on LB-agar with 100 µg/ml ampicillin were selected for plasmid isolation (QIAGEN plasmid mini kit) and plasmid insert examination by HindIII digestion and agarose gel electrophoresis. Transformation plates were washed off with phosphate-buffered saline, and the collected biomass was used for preparation of the plasmid library with the QIAGEN plasmid midi kit.

Transformation of *R. erythropolis* protoplasts was based on previously published work [5,6]: *R. erythropolis* PR4 (NBRC 100887) was grown in LBSG-medium (1% tryptone, 0.5% yeast extract, 0.5% NaCl, 10.3% sucrose, 3% glycine) at 25°C and 200 rpm to OD_{600} 2.7. Biomass from 1 ml of culture was sedimented (5 min, 4500 rcf, 15°C), washed twice with 1 ml buffer B (0.3 M sucrose, 0.01 M $MgCl_2$, 0.025 M Tris-Cl pH 7.2), and resuspended in 1 ml buffer B with 10 mg/ml lysozyme. After 2 h at 37°C with slight agitation, biomass was sedimented (5 min, 1600 rcf, 15°C), washed once with 1 ml buffer P (B with freshly added 2% $CaCl_2$ and 1% KH_2PO_4) and resuspended in 1 ml buffer P. For transformation, 1 µg plasmid library was added per 50 µl aliquot of protoplasts. Reactions were kept at 21°C for 5 min, 50 µl buffer P with 50% polyethylene glycol 6000 was added, and aliquots were gently spread on plates with LBS-agar (1% tryptone, 0.5% yeast extract, 0.5% NaCl, 10.3% sucrose, 0.4% $MgCl_2$, 1,5% agar). After 24 h incubation at 30°C, plates were overlaid with LBS-top-agar (0.6% agar) containing 50 µg/ml thiostreptone, and incubation at 30°C was continued for three days.

To determine the average insert size of the plasmid library in *R. erythropolis*, colonies were picked to inoculate 1.5 ml nutrient broth with 10 µg/ml thiostreptone in deep-well microtiter plates. Biomass was grown at 30°C, 1000 rpm, over three days and used for preparation of plasmid DNA (QIAGEN QIAprep spin miniprep kit; cell lysis supported with 15 mg/ml lysozyme and 200 u/ml mutanolysin) and analysis by agarose gel electrophoresis of HindIII digests.

For investigation of ZEN hydrolyzing activity, individual clones were picked and cultivated as for plasmid insert size determination. Per clone, 200 µl culture was added to 50 µl 50% glycerol, and cryopreserved. The remaining culture was sedimented, and the biomass pellets in deep-well microtiter plates were resuspended in 1 ml Brunner mineral medium with vitamins and 2 mg/l

ZEN. Plates were incubated at 21°C, 1000 rpm, and time point samples (200 µl) were taken and analyzed by LC-MS/MS in pools, and subsequently individually.

ZEN-hydrolysis positive clone P1G2 was used for plasmid preparation and sequencing of the insert flanks with primers F513-532 (5'-GTATGGTGGCAGGCCCCGTG-3') and R586-610 (5'-TAGTAGGTTGAGGCCGTTGAGCACC-3'). The insert was located in the whole genome sequence, and genes were predicted with Glimmer 3.02 [7]. A gene for a predicted alpha/beta hydrolase was amplified with forward primer 5'-TATA*CCATGG*CCGAAGAAGGAACTAGGTCC-3' and reverse primer 5'-TATA*CTCGAG*CTAGTCGTTCGCAGGCAGTGTTGC-3', digested with NcoI and XhoI, and inserted in NcoI – XhoI-cleaved pET-28a(+). The resulting expression vector encoding ZenA without terminal tags was transformed into *E. coli* BL21(DE3) and HMS174(DE3). Cultures of transformed and non-transformed (negative control) clones were grown to OD_{600} in the range of 0.6 to 1.0, induced with 1 mM IPTG, incubated 4 h (33°C, 230 rpm), harvested by centrifugation, resuspended in 1/5 volume Brunner mineral medium with vitamins, and lysed by double passage through a French pressure cell at 20 000 psi. Lysates were spiked with 2 µg/ml ZEN, incubated at 25°C, and time course samples were taken, inactivated (10 min 99°C), and processed for LC-MS/MS analysis.

Characterization of ZenA$_{Re}$

The *zenA$_{Re}$* gene was amplified with the same PCR primers as above, except that the stop codon was omitted from the reverse primer, and inserted in NcoI – XhoI-cleaved pET-28a(+) for expression with a C-terminal 6xHis-tag linked to ZenA$_{Re}$ by LE, encoded by the XhoI site. The plasmid was cloned by transformation of *E. coli* DH10B, and transformed into *E. coli*

ArcticExpress(DE3). For the production of His-tagged $ZenA_{Re}$, ArcticExpress(DE3) was cultivated in LB medium with 50 µg/ml kanamycin at 37°C and then 30°C until OD_{600} 0.65 was reached, chilled to 10°C on ice, induced with IPTG (1 mM final concentration) and incubated at 12°C and 230 rpm for 24 h. Biomass was sedimented by centrifugation, resuspended in 20 mM phosphate buffer pH 7.4 with 0.5 M NaCl and 60 mM imidazole, and lysed by passage through a French press at 20 000 psi. The lysate was cleared by centrifugation and 0.20 µM filtration and applied to a HisTrap HP 1ml column (GE Healthcare) with an ÄKTAprime system. His-tagged $ZenA_{Re}$ was eluted with an imidazole gradient in the same buffer, analyzed by SDS-PAGE, transferred to the same buffer without imidazole by Amicon 10 kDa Ultra-15 (Merck) centrifugal ultrafiltration, quantified by BCA assay (Thermo Fisher), and stored with 50% glycerol at -20°C.

For correlation of $ZenA_{Re}$ activity with temperature, 50 µl aliquots of 35 ng/ml $ZenA_{Re}$ were incubated in Teorell-Stenhagen buffer [8] pH 8.2 with 0.1 mg/ml bovine serum albumin (BSA) and 5 µg/ml ZEN in PCR strips placed in the thermoblock of an Eppendorf Mastercycler set to a temperature gradient. Strips were removed at time intervals, and reactions were stopped by 5 min incubation at 97°C. Concentrations of ZEN, HZEN and DHZEN were determined by LC-MS/MS, HZEN formation rates were calculated from all or, for higher temperatures, just initial time-course points in the linear range, and plotted against the nominal temperature of the thermoblock well.

For correlation of $ZenA_{Re}$ stability with temperature, $ZenA_{Re}$ at a concentration of 17.5 µg/ml was incubated as above but without ZEN. At time intervals, 3 µl samples were taken from each temperature point, added to 1 497 µl 100 mM Tris-Cl pH 8.2 with 0.1 mg/ml BSA and 5 µg/ml ZEN, and incubated at 30°C. From these reactions, 250 µl samples were taken after 0, 30, 60, 120 and 180 min, inactivated (5 min 99°C), and analyzed for ZEN and HZEN concentrations by LC-

MS/MS. ZEN consumption and HZEN formation rates were calculated from time point 0 min and the last time point before completion of the reaction, 120 min or 180 min.

For measuring the stability of $ZenA_{Re}$ with a thermofluor assay [9], 30 µl samples of 100 µg/ml $ZenA_{Re}$ with 5 x SYPRO® Orange stain (Sigma-Aldrich) in 100 mM Tris-Cl pH 8.2, Teorell-Stenhagen buffer pH 8.2, 20 mM phosphate buffer pH 7.4 with 0.5 M NaCl, or water, were exposed to a temperature ramp from 20°C to 95°C with 1°C/min in a Mastercycler ep realplex^2 S (Eppendorf). Fluorescence (excitation; 470 nm, emission: 550 nm) was measured every minute. The change of fluorescence intensity over temperature change was calculated, and the maximum was used as a descriptor of temperature stability.

For measuring the effects of pH on $ZenA_{Re}$, Teorell-Stenhagen buffer was set to pH 3.0, 4.0, 5.0. 5.5, 6.0, 6.5, 7.0, 7.5, 8.0, 8.2, 8.5, 9.0, 9.5, 10.0, 11.0, and 12.0 with HCl [8], and pH was checked and corrected if necessary. For correlation of $ZenA_{Re}$ activity with pH, 35 ng/ml $ZenA_{Re}$ was incubated in this set of buffers with 0.1 mg/ml BSA and 5 µg/ml ZEN. Time course samples were taken after 0, 0.5, 1.0, 1.5, 2.0, 2.5, 3.0, 3.5, 4.0, 4.5, 5.0, 5.5, 6.0, 6.5, and 7.0 h, inactivated (10 min 99°C), and diluted to the fivefold volume with 20% acetonitrile for LC-MS/MS analysis. For correlation of $ZenA_{Re}$ stability with pH, 17.5 µg/ml $ZenA_{Re}$ was incubated in the same set of Teorell-Stenhagen buffers with 0.1 mg/ml BSA but without ZEN at 25°C for one hour, diluted to the five hundredfold volume of Teorell-Stenhagen buffer pH 8.2 with 0.1 mg/ml BSA and 5 µg/ml ZEN, and incubated at 30°C. Time course samples were taken and processed for LC-MS/MS analysis as above.

For determination of kinetic parameters, 25 ng/ml $ZenA_{Re}$ was incubated with ZEN concentrations ranging from 0.25 to 10 µg/ml in 100 mM Tris-Cl pH 8.2 with 0.1 mg/ml BSA at 33°C. Time course samples were taken after 10, 20, 30, 60, 90, 120 and 180 min, inactivated

immediately by exposure to 95°C for 5 min, and processed for HPLC-DAD (diode array detector) analysis. Reaction rates were calculated from initial, linear ZEN concentration decreases.

Characterization of ZenA homologues

Codon-optimized versions of *zenA* genes listed in **Table 1** were synthesized for expression with C-terminal 6xHis-tag and inserted in the NdeI and XhoI sites of pET-3a(+) by GeneArt (Thermo Fisher Scientific). *E. coli* BL21(DE3) was transformed as expression host. Gene expression was performed by cultivation in Overnight Express^{TM} Instant TB Medium (Merck) with 50 µg/ml ampicillin at 30°C with shaking in Erlenmeyer flasks for 24 hours. Procedures for harvesting and lysis of biomass, and for purification by His-tag mediated affinity chromatography and protein quantification were as for $ZenA_{Re}$.

For determination of pH optima, enzymes were incubated in Teorell-Stenhagen buffer with 0.1 mg/ml BSA set to pH 3.0, 4.0, 4.5, 5.0, 5.5, 6.0, 6.5, 7.0, 7.5, 8.0, 8.5, 9.0, 9.5, 10.0, 11.0 or 12.0 and 5 µg/ml ZEN at 30°C. Time point samples were taken over two or three hours, exposed to 95°C for 5 min for inactivation, and processed for quantification of ZEN and HZEN concentrations by HPLC-DAD. Activities were calculated and plotted against pH.

For determination of temperature optima, enzymes in the concentration range 1.5 µg/ml – 40 µg/ml were incubated with 5 µg/ml ZEN in Teorell-Stenhagen buffer pH 7.5 or pH 8.2 with 0.1 mg/ml BSA in wells of PCR strips in an Eppendorf Mastercycler gradient set to a 20°C temperature gradient across the heating block. Temperatures from 10°C to 70°C were covered in separate incubations. Strips were removed after 20 min, 60 min, 120 min and 180 min incubation, exposed to 95°C for 5 min for enzyme inactivation, and reactions were processed for analysis of ZEN,

HZEN and DHZEN concentration by HPLC-DAD. Reaction rates were calculated and plotted against temperature.

For determination of kinetic parameters, enzymes, at a fixed concentration in the range of 25 ng/ml to 40 µg/ml, were incubated under the buffer and temperature conditions listed in **Table 1** with initial ZEN concentrations of 0.25, 0.5, 0.75, 1, 2, 3, 4, 5, 7.5 or 10 µg/ml. Time course samples were taken after 0, 10, 20, 30, 45, 60, 120 and 180 min, exposed to 95°C for 5 min, and processed for HPLC-DAD quantification. Initial reaction rates of ZEN consumption and HZEN generation were calculated, and kinetic parameters were calculated with the Enzyme Kinetics Module of SigmaPlot (https://systatsoftware.com/sigmaplot/).

Thermal unfolding of ZenA homologues was investigated with a thermofluor assay in different buffers. The temperatures shown in **Table 1** are for 20 mM phosphate, 0.5 M NaCl buffer pH 7.4, to which 50% glycerol was added, and 50 µg/ml enzyme concentration. Fluorescence intensities were measured and inflection points were determined as described for $ZenA_{Re}$.

HPLC-DAD and LC-MS/MS quantification of ZEN and derivates

HPLC-DAD analysis was performed as previously described [10]. For LC-MS/MS analysis, samples were diluted to a nominal ZEN concentration of 1 µg/ml with 20% acetonitrile, centrifuged, and analyzed based on a previously established method [11]. Volumes of 10 µl were injected on an Eclipse XDB-C8 column (4.6 x 150 mm, 5 µm, Agilent Technologies) operated by an Agilent 1100 Series HPLC equipped with an API 2000 triple quadrupole MS/MS system (Sciex). A gradient from 20% to 90% methanol in 5 mM ammonium acetate buffer was applied with a flow rate of 0.8 ml/min at 35°C. Analytical standards for HZEN and DHZEN were available from previous work [10].

Pre-steady-state kinetic measurements

Quenched-flow experiments were carried out with a QFM-400 device (BioLogic). Reactions of 2.5 µM $ZenA_{Re}$ and 12.5 µM ZEN in Teorell-Stenhagen buffer pH 8.2 at 11°C were quenched after up to two seconds reaction time. Reactions of 7.5 µM $ZenA_{Scfl}$ and 50 µM ZEN in Teorell-Stenhagen buffer pH 7.5 at 37°C or 2.5 µM $ZenA_{Scfl}$ and 12.5 µM ZEN at 10°C were quenched after up to 9 seconds. Reactions were quenched by addition of 1 M HCl to pH 1.5, collected from the device, and analyzed by HPLC-DAD.

Stopped-flow experiments were carried out on a system composed of an SFM-3000 device coupled to a MOS500 spectrometer (BioLogic), and fluorescence intensity over time was recorded. The excitation wavelength was 275 nm (5 nm slit), and emission was recorded using a longpass filter with 320 nm cutoff so that conformation changes of the protein as well as concentration changes of ZEN and HZEN were covered with the signal. Traces for 1 µM $ZenA_{Re}$ in Teorell-Stenhagen buffer pH 8.2 were recorded with initial ZEN concentrations from 0.125 µM to 12 µM at 11°C, 15°C and 19°C. Product binding was measured in the same buffer at 8°C and HZEN-concentrations from 1.25 µM to 75 µM. For $ZenA_{Scfl}$, stopped-flow fluorescence intensity traces were recorded in Teorell-Stenhagen buffer pH 7.5 at 37°C. For single turnover experiments, the concentration of $ZenA_{Scfl}$ was 12.5 µM, and the initial ZEN concentration ranged from 0.3125 µM to 10 µM. Multiple turnover experiments were done with 2 µM $ZenA_{Scfl}$ and ZEN concentrations from 0.3125 µM to 75 µM. Product binding was measured with 2 µM $ZenA_{Scfl}$ and HZEN-concentrations from 0.3125 µM to 75 µM.

Kinetic Data Analysis and Statistics

The kinetic model was built using the numerical integration method. During this analysis, the kinetic data were fit globally with the KinTek Explorer program (KinTek, USA), a dynamic kinetic simulation program that allows multiple data sets to be fit simultaneously to a single model. During the numerical analysis, the reaction time course was computed based on a unique set of rate constants and output factors that define the relationship between the concentrations of reactants and/or products and the observable signal. Information about the absolute concentration of species, from steady-state product formation and a quench-flow burst experiment monitoring the time dependence of substrate consumption and product formation, was synergically combined with spectroscopic data defined as a weighted sum of species with variable scaling factors. Data fitting was then achieved by finding rate constants and scaling factors to generate a signal that reproduces the data. Thus, surpassing conventional analytical data fitting, the process yields intrinsic rate constants directly and can precisely deconvolute even complex contributions of reaction species to the observed signal [12].

The most important aspect of this mechanism-based numerical data fitting is the ability to fit multiple experiments simultaneously. With a single unifying model, experiments differ only in the starting conditions and the definition of the output observable function. Unlike equation-based data fitting where each different experimental design may require a different equation, with mechanism-based data fitting, numerical integration of the rate equations from a unique starting state for each experiment allows all experiments to be fit to derive a single set of rate constants to account for all of the data. Remarkably, this analysis has demonstrated that the whole really is greater than the sum of the parts. In contrast, the simplifying approximations and errors in equation-based data fitting preclude the complete resolution of the full reaction pathway [13].

During the data fitting, the KinTek program applied numerical integration of rate equations from an input model searching a set of parameters using the Bulirsch–Stoer algorithm with an adaptive step size that produces a minimum $\chi 2$ value calculated by using nonlinear regression based on the Levenberg-Marquardt method [12]. Residuals were normalized by sigma value for each data point. The observable fluorescence signal was defined as the sum of the contributions of each species to the total fluorescence (**Equation 1**) with scaling factors for each species, where *f* scales the signal to concentration and sensitivity of the measurement, factors *es, esc, ei, epc* and *ep* define the relative change in fluorescence in forming ES, E´S, E´I, E´P and EP complexes, respectively. The factor *p* scales the signal contribution of the fluorogenic product HZEN.

$$Signal = f * (E + es * ES + esc * E'S + ei * E'I + epc * E'P + ep * EP + p * (P + EP + E'P)) \qquad \text{(Eq. 1)}$$

Different fluorescence scaling factors relating to the different sensitivity of spectrophotometric setup (*f*) were used for data at each temperature, but the relative change in fluorescence in forming individual complexes was constant for all datasets.

The contributions of individual reactive species to the fluorescence signal were first identified and quantified within the analytical data fit, and then consistently used to construct an appropriate scaling function for numerical fitting. The omission of any of the observed effects from the scaling function would have led to undesired simplification and incorrectness in the final model. Thanks to the presence of multiple tryptophan residues in the structure of ZenA, fluorescence data provided a wealth of information on all individual catalytic steps without the need for labeling. For accuracy, it is essential to state that the fluorescence of the substrate was not considered for the explanation of the fluorescence data because the substrate fluorescence was negligible and did

not have any observable contribution to the total fluorescence signal recorded during the reaction under the given experimental conditions and concentrations. On the contrary, the contribution of the fluorescence of the product could be well recognized and the effects clearly quantified from the experimental data, which will be described in detail later in the text. Any contributions from solvation effects and non-specific protein-protein/protein-ligand interactions were not detected and therefore not included in scaling the fluorescence signal.

Individual effects on the fluorescence signal and their magnitudes were systematically estimated from the experimental data. The parameters defining the change in enzyme fluorescence between its free form (E), complex with substrate (ES) and complex after a conformational change (E´S) were well defined from the initial phase of the reaction (approx. first 50 ms for $ZenA_{Re}$ reaction, Figure 5 F-I). During these rapid initial interactions of the substrate with the enzyme, the concentration of the formed product is close to zero and thus has a negligible contribution to the total fluorescence signal including possible contributions from complexes with the enzyme. The signal is a combination of the contribution of the free enzyme and the initial enzyme complexes with the substrate. The time development of the fluorescence signal then made it possible to clearly divide the individual phases and the factors corresponding to them that change the fluorescence of the given state. After mixing the enzyme with the substrate, the first change in fluorescence occurred, which was clearly visible as the dependence of the initial fluorescence level on the concentration of the substrate (**Figure 3A**). The dependence of the amplitude of this phase on the concentration of the substrate (**Figure 3C**, upper graph) provided the first estimate of the equilibrium dissociation constant of the enzyme-substrate complex and estimation of the value of the scaling factor of the fluorescence change between the free enzyme (E) and the enzyme-substrate complex (ES). The binding of the substrate was fast, reaching rapid equilibrium. A

kinetic phase was not observed since it was lost in the dead time of the measurement (0.3 ms). The initial rapid equilibrium binding phase was followed by two kinetic phases, a fast and a slow one (**Figure 3A**). The hyperbolic shape of the dependence of the rate on the substrate concentration (**Figure 3B**) obtained for the fast phase clearly confirmed that this kinetic phase was not related to initial substrate binding, but reflected a subsequent isomerization step. The dependence of the amplitude of the fast phase (A_1) on the concentration of the substrate further confirmed that it is a step following the binding step. $K_{D,app}$ obtained from the amplitude dependence was significantly lower than K_D obtained from the concentration dependence of the observed rates (k_{obs}), which was practically equal to the dissociation constant of the enzyme-substrate complex. The complex trend in the A_1 concentration profile, which indicated two equilibria, further confirmed that the fluorescence change occured during both substrate binding and the following fast isomerization step (**Figure 3C**, lower graph). These data also provided an initial estimate of the scaling factors, which was subsequently used as a starting value for global numerical analysis. The third slow step was also clearly distinguished in the fluorescence data (**Figure 3A**, **3B**), and the analytical fit also provided the initial estimate of the change in the fluorescence signal related to the formation of an intermediate (E´I). A detailed analysis of the initial phases of the reaction thus provided information about the three initial steps and made it possible to obtain an initial estimate of the related rates and equilibrium constants, but also the initial quantitative estimates for all scaling factors, thanks to which these phases could be numerically simulated and used in global kinetic analysis. In a similar way, the fluorescence contributions of the product and the enzyme-product complex were defined precisely in the product binding data (**Figure 3G**). The product binding experiment and the recorded fluorescence data were not affected by the contributions of any enzyme-substrate complexes (ES, E´S) that do not occur in such an experiment. The exact value

of the product fluorescence contribution was numerically derived from the initial fluorescence level of the kinetic traces obtained by mixing different concentrations of the product with a constant low concentration of the enzyme (initial fluorescence at the time point t = 0 s in **Figure 5L**). The steady-state product formation was numerically simulated as a total product formed during the reaction and occurring in all forms (signal = E´P + EP + P). Similarly, the signal from the quench-flow burst experiment monitoring the time dependence of substrate consumption and product formation was defined as the total substrate (signal = ES + E´S + S) and product (signal = E´P + EP + P) in all forms accessible for the analytical detection (HPLC). With the help of initial calculations using a conventional analytical method, but also during global fitting, we gradually tested and confirmed the presence of every scaling parameter. All along, we followed the rule of thumb that every parameter must be justified in terms of a better explanation of the experimental observation, increased goodness of the fit and improvement in the statistical parameters of the resulting model. Step by step, we gradually defined the final scaling function and kinetic model which was systematically evaluated during the global analysis. Fitting the fluorescence data in parallel with the concentration profiles, both collected at the same experimental conditions, made it possible to precisely define the concentrations of individual reaction species at individual time points and numerically deconvolute the contributions of different enzyme forms and/or product to the observed fluorescence signal. The data describing the concentration of the reaction species (e.g., quench-flow data or steady-state data) anchor the interpretation of fluorescence signals [13].

To account for slight variations in the data, enzyme or substrate concentrations were allowed to vary to make the best fits possible. The standard error (S.E.) was calculated from the covariance matrix during nonlinear regression. In addition to S.E. values, a more rigorous analysis of the variation of the kinetic parameters was accomplished by confidence contour analysis with FitSpace

Explorer (KinTek, USA) [14]. In this analysis, the lower and upper limits for each parameter were derived from the confidence contours for χ^2 threshold at boundary 0.98. Generally, confidence contours provide a striking indication of any overparameterization, reveal complex relationships between parameters when they are not constrained, and provide realistic limits of error on fitted parameters [14]. Our present, rigorous confidentiality analysis with confidence contours verified that all fluorescence contributions in the scaling function, which translates information about the concentration of individual species into information about the current fluorescence level, the scaling factors, as well as the rate constants defining the model, are well constrained by an extensive data set (**Table S2**, Supporting Information).

The dependence of kinetic parameters on temperature was analyzed using the Arrhenius (Equation 2) and Eyring (Equation 3) models to estimate the activation energy (E_a), enthalpy ($\Delta H^{\ddagger}$), and entropy of activation ($\Delta S^{\ddagger}$) for both kinetic phases, where R is the universal gas constant, k_B is the Boltzmann constant, h is Planck's constant, and A is the frequency factor. Linear fits of ln(k) or ln(k/T) versus 1/T were weighted for the standard errors of the rate estimates.

$$\ln(k) = \ln(A) - \frac{E_a}{R.T} \qquad \text{(Eq. 2)}$$

$$\ln(k/T) = -\frac{\Delta H^{\ddagger}}{R.T} + \ln\left(\frac{k_B}{h}\right) + \frac{\Delta S^{\ddagger}}{R} \qquad \text{(Eq. 3)}$$

SEC-MALS

SEC-MALS was performed using a Superose 6 Increase (Cytiva) column for ZenA$_{Re}$ or a Superdex Increase 200 10/300 GL (Cytiva) column for ZenA$_{Scfl}$ operated at 20°C with a 1260 Infinity HPLC system (Agilent Technologies) coupled to a miniDawn Treos MALS detector

(Wyatt Technology). Proteins were concentrated using Amicon Ultra centrifugal filter units with 10 kDa nominal molecular weight limit. 120 µl of $ZenA_{Re}$ at 3.3 mg/mL were injected into the column equilibrated with 25 mM HEPES pH 7.0, 150 mM NaCl. For $ZenA_{Scfl}$, 50 µL of sample at a concentration of 6.3 mg/mL were injected into the column equilibrated with phosphate-buffered saline. Protein concentration was measured with a RI-101 refractive index detector (Shodex) and average molecular weight was calculated with the program Astra (Wyatt Technology). The first-order fit Zimm formalism was used for analysis of light scattering data as data process procedure in Astra, and a generic protein dn/dc value of 0.185 ml/g was used for guanidinase and BSA.

Site-directed mutagenesis

The QuikChange® Site-Directed Mutagenesis Kit (Stratagene/Agilent) was used to exchange codons of *zenA* genes in pET-vectors according to the manufacturer's instructions. Enzyme variants were produced in *E. coli* and assayed in clarified cell lysate or purified by His-tag mediated affinity chromatography like wild-type enzymes.

Production of selenomethionine-substituted $ZenA_{Scfl}$

The plasmid with *zenA*$_{Scfl}$ inserted in pET-3a(+) was transformed into methionine auxotroph *E. coli* B834(DE3). A pre-culture of this expression strain was grown overnight at 37°C and 140 rpm in SelenoMet medium (Molecular Dimensions) with L-methionine. Biomass was harvested by centrifugation, washed 3 x with sterile water, resuspended in sterile water, and used to inoculate SelenoMet medium with L-selenomethionine. After 1.5 h incubation at 37°C and 170 rpm, OD_{600} 0.6 was reached, the culture was induced with 1 mM IPTG, and incubation was continued for 6 h

at 30°C and 170 rpm. Biomass was harvested by centrifugation, stored frozen, and lysed by passage through a French press at 20 000 psi. Selenomethionine-substituted $ZenA_{Scfl}$ was purified from the clear lysate by IMAC, SEC and centrifugal ultrafiltration, and subjected to crystallization screening.

Crystallization

ZenA$_{Scfl}$ was concentrated to 10 mg/mL with an Amicon Ultra centrifugal filter (10 kDa) in 50 mM phosphate buffer pH 7.0. It was crystallized in the condition 0.1 M imidazole/MES pH 6.5, 0.06 M $MgCl_2$, $CaCl_2$, 30% w/v {40% ethylene glycol, 20% PEG 8000} using the vapor diffusion sitting drop setup in MRC 2-well crystallization plates prepared by a Phoenix crystallization robot.

ZenA$_{Scfl}$ H286Y was concentrated to 10 mg/mL in 50 mM phosphate buffer pH 7.0 with an Amicon Ultra centrifugal filter (10 kDa). Initial screening was performed with a Mosquito crystallization robot (SPT Labtech) using MRC 3-well plates and protein to crystallant solution ratios of 150 nL to 200 nL, 200 nL to 200 nL, and 250 nL to 200 nL. The enzyme crystallized initially in the condition H8 (0.1 M Mg formate,15% w/v PEG 3350) of the Index Screen (Hampton Research) at room temperature. The ligand derivative structure with zearalenone was obtained by co-crystallization in presence of 5 mM zearalenone which was pre-dissolved at 100 mM in EtOH using the optimized condition 0.1 M Mg formate and 11.09% w/v PEG 3350 at room temperature.

ZenA$_{Re}$ was concentrated to 7 mg/mL with a 10 kDa Amicon Ultra centrifugal filter. Protein crystals formed in the condition H8 (0.2 M NaCl, 0.1 M Bis-Tris pH 5.5, 25% w/v PEG3350) of the JCSG-plus^{TM} screen (Molecular Dimensions) at 4°C.

Structure determination

Crystals were fished using cryo-loops, cryo-protected using the crystallization condition supplemented with 20% glycerol, and flash-frozen in liquid nitrogen. For ZenA$_{Re}$ with bound zearalactamenone (ZLAEN, a hydrolysis-resistant ZEN-analog synthesized by ChiroBlock, Germany, **Figure S15**, Supporting Information), crystals were soaked for 40 min at 4°C in the crystallization condition supplemented with 20% glycerol and 5 mM ZLAEN, and flash-frozen in

liquid nitrogen. For ZenA$_{Re}$ with bound HZEN, ZenA$_{Re}$ was co-crystallized with 5 mM ZEN, added as 100 mM ZEN in EtOH stock solution, in JCSG H8 at 4°C, cryo-protected with 20% glycerol, and flash-frozen in liquid nitrogen.

Datasets were collected at 100 K at Beamlines ID23-1, ID23-2, ID-30B, ID29 and ID30A3 at the European Synchrotron Radiation Facility (ESRF, France) and at Beamlines I03 and I04 of the Diamond Light Source (Great Britain). The Datasets were processed with XDS and converted using XDSCONV [15]. In the case of ZenA$_{Re}$ in complex with HZEN, two datasets collected on one crystal were merged using XSCALE. The initial structure was solved employing the SAD method using the CRANK-2 automatic pipeline from the CCP4 program suite [16] on a dataset collected on a SeMet derivative structure. The phase problem of subsequent structures was solved with Phaser-MR [17] using the SAD structure as a search template. The structures were further refined in iterative cycles of a manual model building using COOT [18] and maximum-likelihood refinement using the PHENIX software suite [19]. The final stages of refinement used automated addition of hydrogens and water molecules, optimization of X-ray/ADP weight and optimization of X-ray/stereochemistry weight. The model was validated with MolProbity [20] and PDBREDO [21]. Data collection and refinement statistics are listed in **Table S3** (Supporting Information). Figures were created with PyMOL (The PyMOL Molecular Graphics System, Version 2.0 Schrödinger, LLC.).

References

(1) Lemke, S. L.; Grant, P. G.; Phillips, T. D. Adsorption of Zearalenone by Organophilic Montmorillonite Clay. *J. Agric. Food Chem.* **1998**, *46* (9), 3789–3796. https://doi.org/10.1021/jf9709461.

(2) Weisburg, W. G.; Barns, S. M.; Pelletier, D. A.; Lane, D. J. 16S Ribosomal DNA Amplification for Phylogenetic Study. *J. Bacteriol.* **1991**, *173* (2), 697–703. https://doi.org/10.1128/jb.173.2.697-703.1991.

(3) Aziz, R. K.; Bartels, D.; Best, A. A.; DeJongh, M.; Disz, T.; Edwards, R. A.; Formsma, K.; Gerdes, S.; Glass, E. M.; Kubal, M.; Meyer, F.; Olsen, G. J.; Olson, R.; Osterman, A. L.; Overbeek, R. A.; McNeil, L. K.; Paarmann, D.; Paczian, T.; Parrello, B.; Pusch, G. D.; Reich, C.; Stevens, R.; Vassieva, O.; Vonstein, V.; Wilke, A.; Zagnitko, O. The RAST Server: Rapid Annotations Using Subsystems Technology. *BMC Genomics* **2008**, *9* (1), 75. https://doi.org/10.1186/1471-2164-9-75.

(4) Singer, M. E.; Finnerty, W. R. Construction of an *Escherichia coli-Rhodococcus* Shuttle Vector and Plasmid Transformation in *Rhodococcus* Spp. *J. Bacteriol.* **1988**, *170* (2), 638–645.

(5) Dabbs, E. R.; Sole, G. J. Plasmid-Borne Resistance to Arsenate, Arsenite, Cadmium, and Chloramphenicol in a *Rhodococcus* Species. *MGG Mol. Gen. Genet.* **1988**, *211* (1), 148–154. https://doi.org/10.1007/BF00338406.

(6) Seth-Smith, H. M. B.; Rosser, S. J.; Basran, A.; Travis, E. R.; Dabbs, E. R.; Nicklin, S.; Bruce, N. C. Cloning, Sequencing, and Characterization of the Hexahydro-1,3,5-Trinitro-1,3,5-Triazine Degradation Gene Cluster from *Rhodococcus rhodochrous*. *Appl. Environ. Microbiol.* **2002**, *68* (10), 4764–4771. https://doi.org/10.1128/AEM.68.10.4764-4771.2002.

(7) Delcher, A. L.; Bratke, K. A.; Powers, E. C.; Salzberg, S. L. Identifying Bacterial Genes and Endosymbiont DNA with Glimmer. *Bioinformatics* **2007**, *23* (6), 673–679. https://doi.org/10.1093/bioinformatics/btm009.

(8) Teorell, T.; Stenhagen, E. Ein Universalpuffer für den pH-Bereich 2,0 bis 12,0. *Biochem. Z.* **1938**, *299*, 416–419.

(9) Huynh, K.; Partch, C. L. Analysis of Protein Stability and Ligand Interactions by Thermal Shift Assay. *Curr. Protoc. Protein Sci.* **2015**, *79* (1), 28.9.1–28.9.14. https://doi.org/10.1002/0471140864.ps2809s79.

(10) Vekiru, E.; Fruhauf, S.; Hametner, C.; Schatzmayr, G.; Krska, R.; Moll, W. D.; Schuhmacher, R. Isolation and Characterisation of Enzymatic Zearalenone Hydrolysis Reaction Products. *World Mycotoxin J.* **2016**, *9* (3), 353–363. https://doi.org/10.3920/WMJ2015.2005.

(11) Hahn, I.; Kunz-Vekiru, E.; Twarużek, M.; Grajewski, J.; Krska, R.; Berthiller, F. Aerobic and Anaerobic *in vitro* Testing of Feed Additives Claiming to Detoxify Deoxynivalenol and Zearalenone. *Food Addit. Contam. - Part A Chem. Anal. Control. Expo. Risk Assess.* **2015**, *32* (6), 922–933. https://doi.org/10.1080/19440049.2015.1023741.

(12) Johnson, K. a.; Simpson, Z. B.; Blom, T. Global Kinetic Explorer: A New Computer Program for Dynamic Simulation and Fitting of Kinetic Data. *Anal. Biochem.* **2009**, *387* (1), 20–29. https://doi.org/10.1016/j.ab.2008.12.024.

(13) Johnson, K. A. History of Advances in Enzyme Kinetic Methods: From Minutes to Milliseconds. In *The Enzymes*; Kaguni, L. S., Tamanoi, F., Eds.; Academic Press, 2023; pp 107–134. https://doi.org/10.1016/bs.enz.2023.07.005.

(14) Johnson, K. a.; Simpson, Z. B.; Blom, T. FitSpace Explorer: An Algorithm to Evaluate Multidimensional Parameter Space in Fitting Kinetic Data. *Anal. Biochem.* **2009**, *387* (1), 30–41. https://doi.org/10.1016/j.ab.2008.12.025.

(15) Kabsch, W. XDS. *Acta Crystallogr. Sect. D Biol. Crystallogr.* **2010**, *66* (2), 125–132. https://doi.org/10.1107/S0907444909047337.

(16) Winn, M. D.; Ballard, C. C.; Cowtan, K. D.; Dodson, E. J.; Emsley, P.; Evans, P. R.; Keegan, R. M.; Krissinel, E. B.; Leslie, A. G. W.; McCoy, A.; McNicholas, S. J.; Murshudov, G. N.; Pannu, N. S.; Potterton, E. a.; Powell, H. R.; Read, R. J.; Vagin, A.; Wilson, K. S. Overview of the CCP4 Suite and Current Developments. *Acta Crystallogr. Sect. D Biol. Crystallogr.* **2011**, *67* (4), 235–242. https://doi.org/10.1107/S0907444910045749.

(17) McCoy, A. J.; Grosse-Kunstleve, R. W.; Adams, P. D.; Winn, M. D.; Storoni, L. C.; Read, R. J. Phaser Crystallographic Software. *J. Appl. Crystallogr.* **2007**, *40* (4), 658–674. https://doi.org/10.1107/S0021889807021206.

(18) Emsley, P.; Lohkamp, B.; Scott, W. G.; Cowtan, K. Features and Development of Coot. *Acta Crystallogr. Sect. D Biol. Crystallogr.* **2010**, *66* (4), 486–501. https://doi.org/10.1107/S0907444910007493.

(19) Liebschner, D.; Afonine, P. V.; Baker, M. L.; Bunkoczi, G.; Chen, V. B.; Croll, T. I.; Hintze, B.; Hung, L. W.; Jain, S.; McCoy, A. J.; Moriarty, N. W.; Oeffner, R. D.; Poon, B. K.; Prisant, M. G.; Read, R. J.; Richardson, J. S.; Richardson, D. C.; Sammito, M. D.; Sobolev, O. V.; Stockwell, D. H.; Terwilliger, T. C.; Urzhumtsev, A. G.; Videau, L. L.; Williams, C. J.; Adams, P. D. Macromolecular Structure Determination Using X-Rays, Neutrons and Electrons: Recent Developments in Phenix. *Acta Crystallogr. Sect. D Struct. Biol.* **2019**, *75*, 861–877. https://doi.org/10.1107/S2059798319011471.

(20) Chen, V. B.; Arendall, W. B.; Headd, J. J.; Keedy, D. a.; Immormino, R. M.; Kapral, G. J.; Murray, L. W.; Richardson, J. S.; Richardson, D. C. MolProbity: All-Atom Structure Validation for Macromolecular Crystallography. *Acta Crystallogr. Sect. D Biol. Crystallogr.* **2010**, *66* (1), 12–21. https://doi.org/10.1107/S0907444909042073.

(21) Joosten, R. P.; Long, F.; Murshudov, G. N.; Perrakis, A. The PDB-REDO Server for Macromolecular Structure Model Optimization. *IUCrJ* **2014**, *1*, 213–220. https://doi.org/10.1107/S2052252514009324.

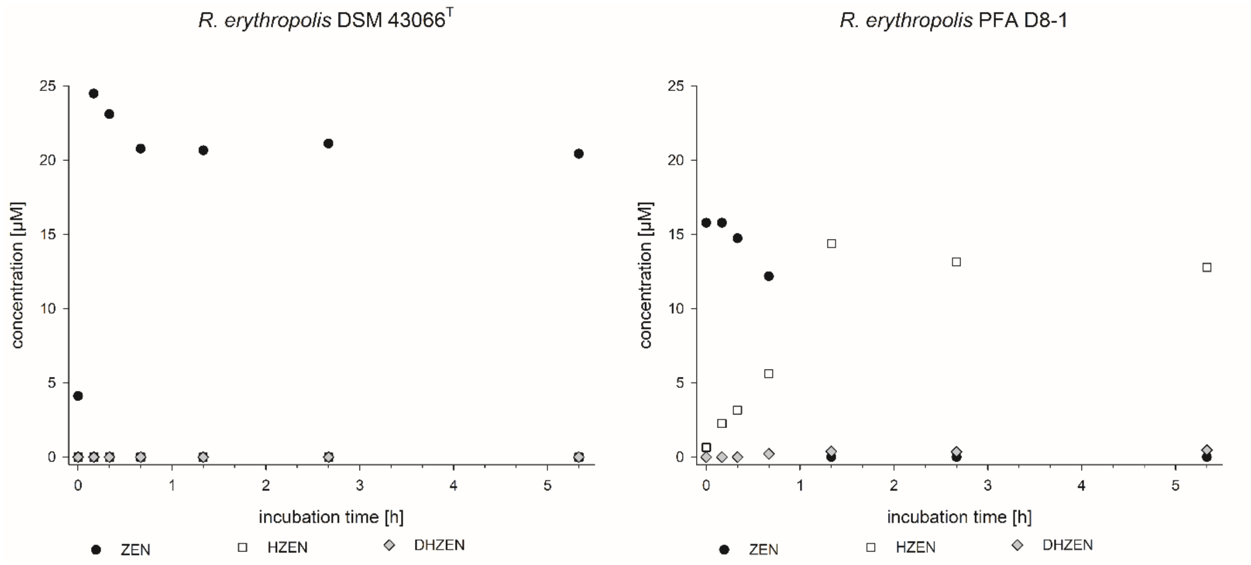

Figure S1. Hydrolysis of ZEN by *R. erythropolis* strains.

Biomass was grown to stationary phase in nutrient broth and transferred to the same volume of Brunner mineral medium with added vitamins and ZEN for time-point sampling of ZEN hydrolysis.

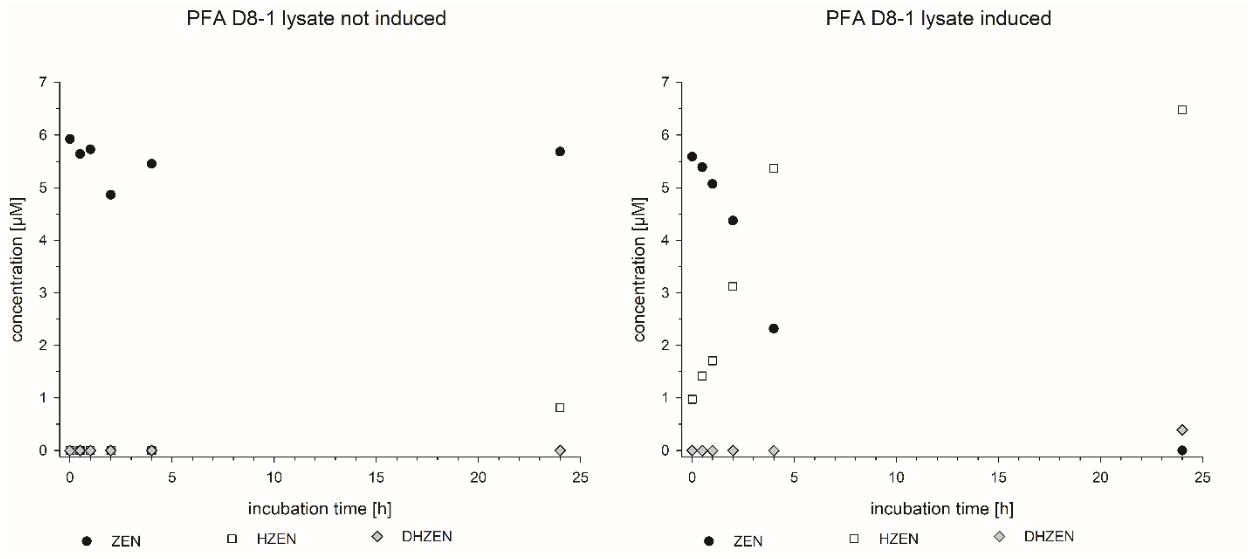

Figure S2. Hydrolysis of ZEN by clear lysate of *R. erythropolis* PFA D8-1.

Biomass was grown in nutrient broth with (“induced”) or without (“not induced”) ZEN, sedimented and resuspended in the same volume of Brunner mineral medium with added vitamins, lysed (French press), and cleared by centrifugation and filtration. ZEN was added for time-point sampling of ZEN hydrolysis.

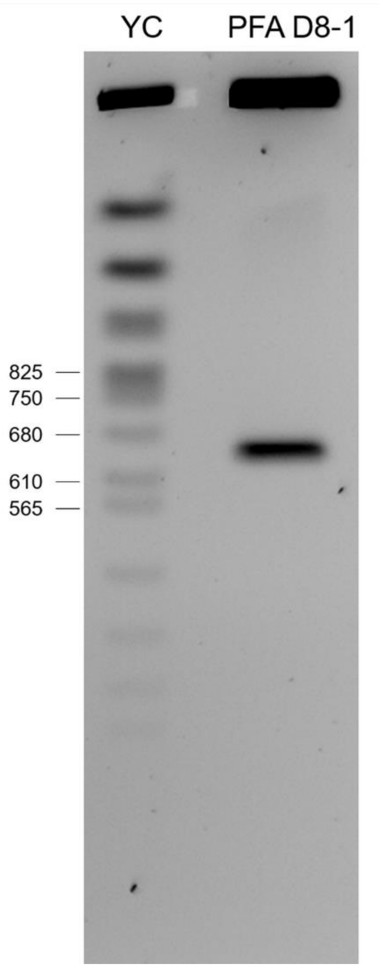

Figure S3. Pulsed field agarose gel with DNA of *R. erythropolis* PFA D8-1.

Yeast chromosomes (YC) served as molecular size marker. The 660 kbp linear megaplasmid was named pSFRL1.

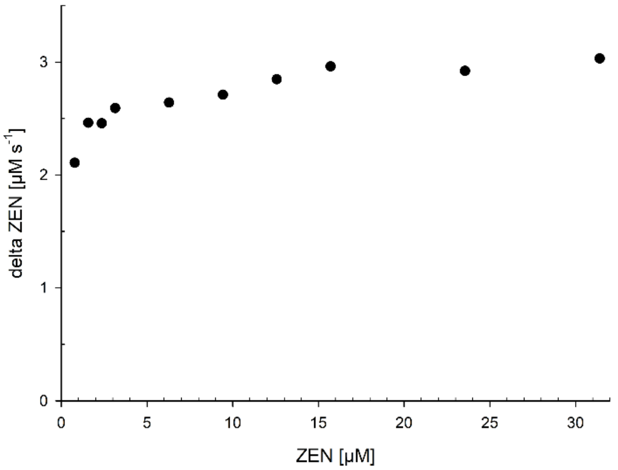

Figure S4. Steady-state ZEN hydrolysis velocity of $ZenA_{Re}$ at various ZEN concentrations.
Velocities were measured with 25 ng/ml $ZenA_{Re}$ in 100 mM Tris-Cl pH 8.2 with 0.1 mg/ml BSA at 33°C and are displayed as µM s^{-1} ZEN concentration reduction for a concentration of 1 µM $ZenA_{Re}$ monomers.

Table S1. The initial values of the kinetic parameters.

The initial estimates of the rate and equilibrium constants were obtained from analytical fitting. The parameters were used as starting values for global numerical analysis.

		ZenA$_{Re}$				***ZenA$_{Scfl}$***	
Temperature		292 K (19°C)	288 K (15°C)	284 K (11°C)	281 K (8°C)	310 K (37°C)	281 K (8°C)
K_S	µM	10	10	6	6	9.0	38
k_2	s^{-1}	1 000	560	390	90	630	400
k_{-2}	s^{-1}	9	19	4.0	4.6	160	8.6
k_3	s^{-1}	12	9.5	10	6	0.3	0.3
k_4	s^{-1}	5.2	3.9	1.5	2	n.d.	n.d.
k_5	s^{-1}	n.d.	n.d.	n.d.	9	48	n.d.
k_{-5}	s^{-1}	n.d.	n.d.	n.d.	270	2 300	n.d.
K_P	µM	n.d.	n.d.	n.d.	480	77	n.d.

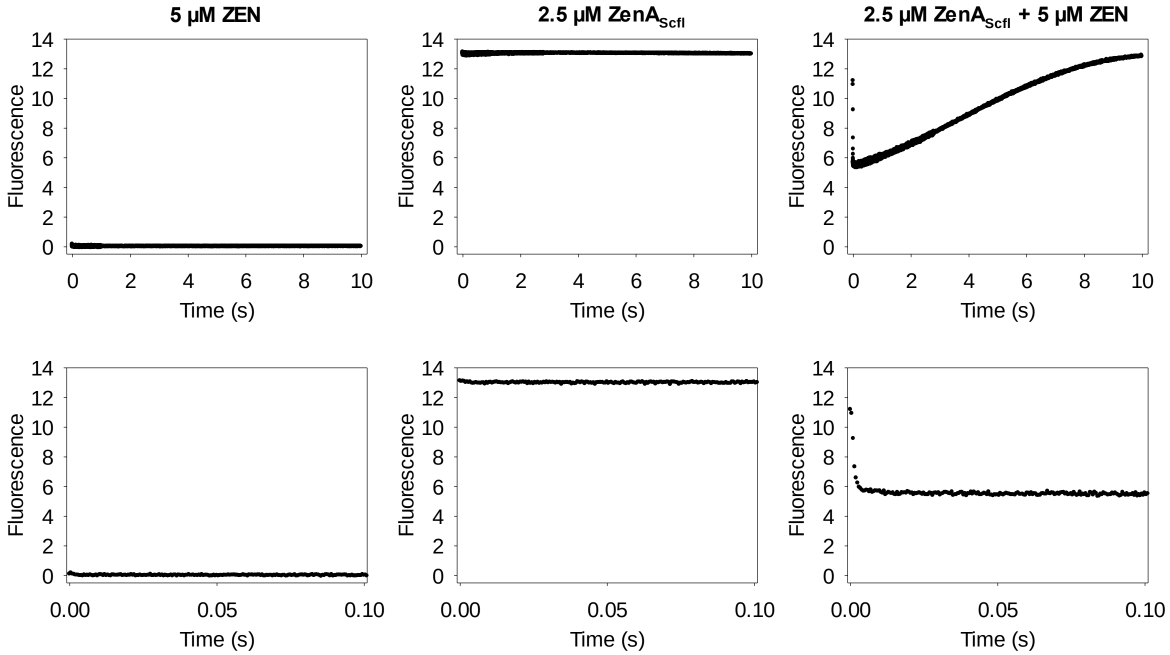

Figure S5. Stopped-flow fluorescence intensity readings for ZEN, ZenA$_{Scfl}$, and ZenA$_{Scfl}$ + ZEN. Five µM ZEN (left), 2.5 µM ZenA$_{Scfl}$ (middle) or 2.5 µM ZenA$_{Scfl}$ + 5 µM ZEN (right) in Teorell-Stenhagen buffer pH 7.5 were injected into the stopped-flow system set up as described in Materials and Methods (Supporting Information) at 37°C. Fluorescence intensity readings are shown for the first 10 s (top row) and for the first 0.1 s (bottom row).

Table S2. The kinetic and thermodynamic parameters obtained by global fit.

The best-fit estimates of the kinetic parameters of the catalytic cycle of $ZenA_{Re}$ and $ZenA_{Scfl}$ during ZEN-hydrolysis were obtained by nonlinear regression based on numerical integration of the rate equations derived from the input kinetic model (**Figure 4A**). The standard error (± SE) was calculated from the covariance matrix during nonlinear regression. Confidence intervals (lower and upper limits) of the parameters were obtained by confidence contour analysis for χ^2 threshold of 0.98 (**Figure S6** and **Figure S8**, Supporting Information). The reference temperature is 284 K and 310 K for $ZenA_{Re}$ and $ZenA_{Scfl}$, respectively.

		**$ZenA_{Re}$ at 284 K (11°C)**			**$ZenA_{Scfl}$ at 310 K (37°C)**		
		best-fit ± s.e.	lower limit	upper limit	best-fit ± s.e.	lower limit	upper limit
K_S	µM	8.3 ± 0.4	4.2	44.8	10 ± 6	6.6	25.2
k_2	s^{-1}	460 ± 20	275	2 100	2 200 ± 100	1 770	4 900
k_{-2}	s^{-1}	2.8 ± 0.1	0.969	5.8	45 ± 1	38.1	63.1
k_3	s^{-1}	8.6 ± 0.1	6.67	11.5	0.40 ± 0.01	0.381	0.436
k_4	s^{-1}	1.64 ± 0.01	1.52	1.8	11 ± 1	7.69	15.9
k_5	s^{-1}	1.35 ± 0.01	1.15	1.55	47 ± 3	24.7	94
k_{-5}	s^{-1}	530 ± 10	383	945	> 1 100	1 100	n.d.
K_P	µM	610 ± 20	388	1 182	55 ± 2	35	68
es		0.75 ± 0.02	0.722	0.759	0.40 ± 0.01	0.273	0.515
esc		0.182 ± 0.003	0.161	0.196	0.287 ± 0.001	0.28	0.295
ei		< 0.01	n.d.	n.d.	< 0.01	n.d.	n.d.
epc		0.891 ± 0.001	0.881	0.895	0.805 ± 0.002	0.789	0.815
ep		1.5 ± 0.1	1.32	n.d.	0.99 ± 0.01	0.923	1.06
p		0.0069 ± 0.0001	0.0069	0.0070	0.0061 ± 0.0001	0.00581	0.00629
$E_{a,k2}$	kJ mol^{-1}	73 ± 1	51.8	95.4	n.d.	n.d.	n.d.
$E_{a,k3}$	kJ mol^{-1}	148 ± 2	114	185	n.d.	n.d.	n.d.
$E_{a,k4}$	kJ mol^{-1}	8.6 ± 3	4.97	16.2	n.d.	n.d.	n.d.
$E_{a,k5}$	kJ mol^{-1}	80 ± 1	54.7	95	n.d.	n.d.	n.d.

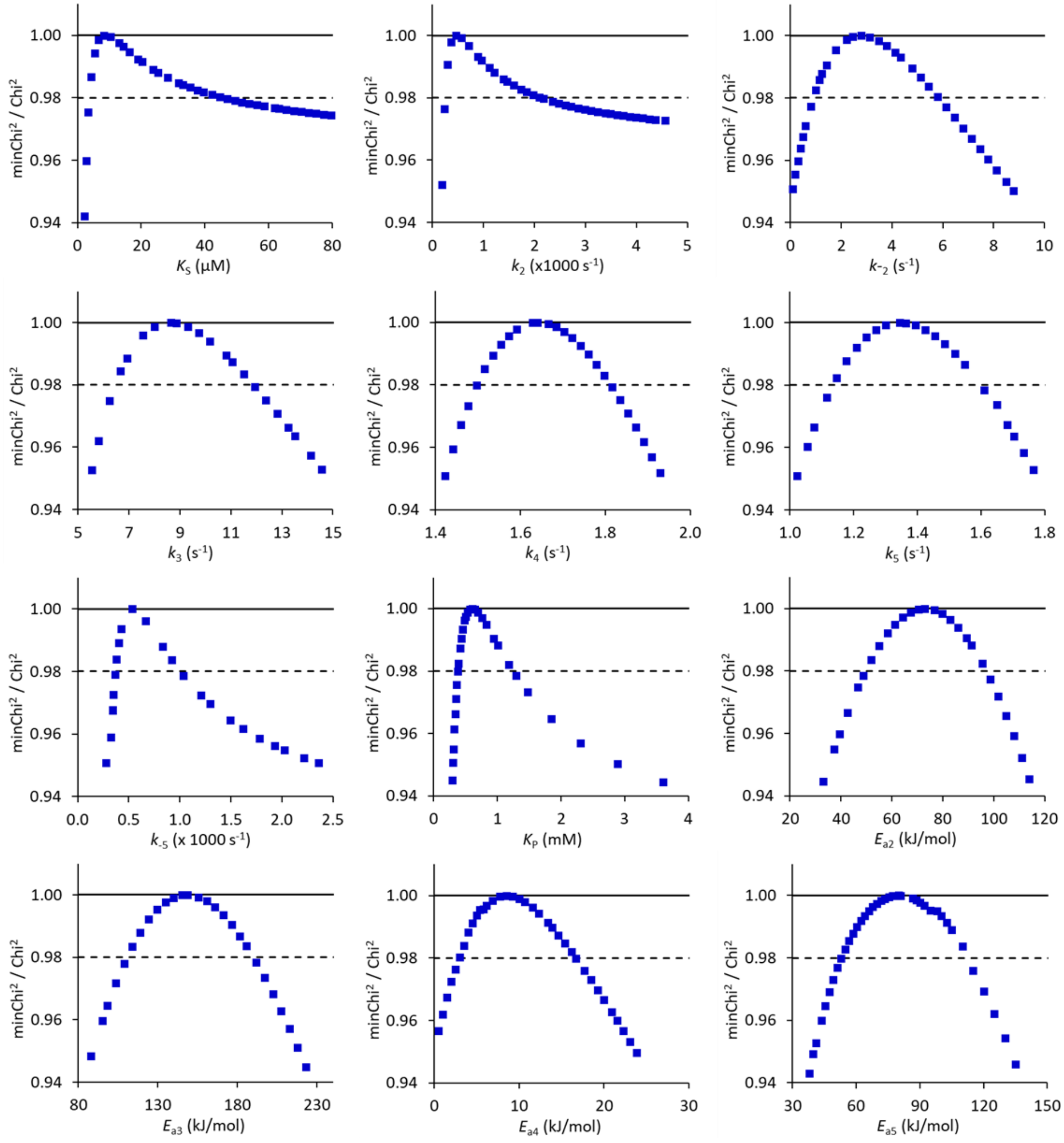

Figure S6. Confidence contour analysis of $ZenA_{Re}$ kinetic and thermodynamic parameters.

Error analysis of the fitted parameters on the kinetic constants and energy barriers obtained from the global fit of $ZenA_{Re}$ kinetic data. Individual figures represent the dependence of the error on the respective fitted parameter while varying all other parameters to achieve the best fit. In every case, the dashed line shows the $\chi 2$ threshold (0.98) used to establish confidence intervals as reported in **Table S2** (Supporting Information).

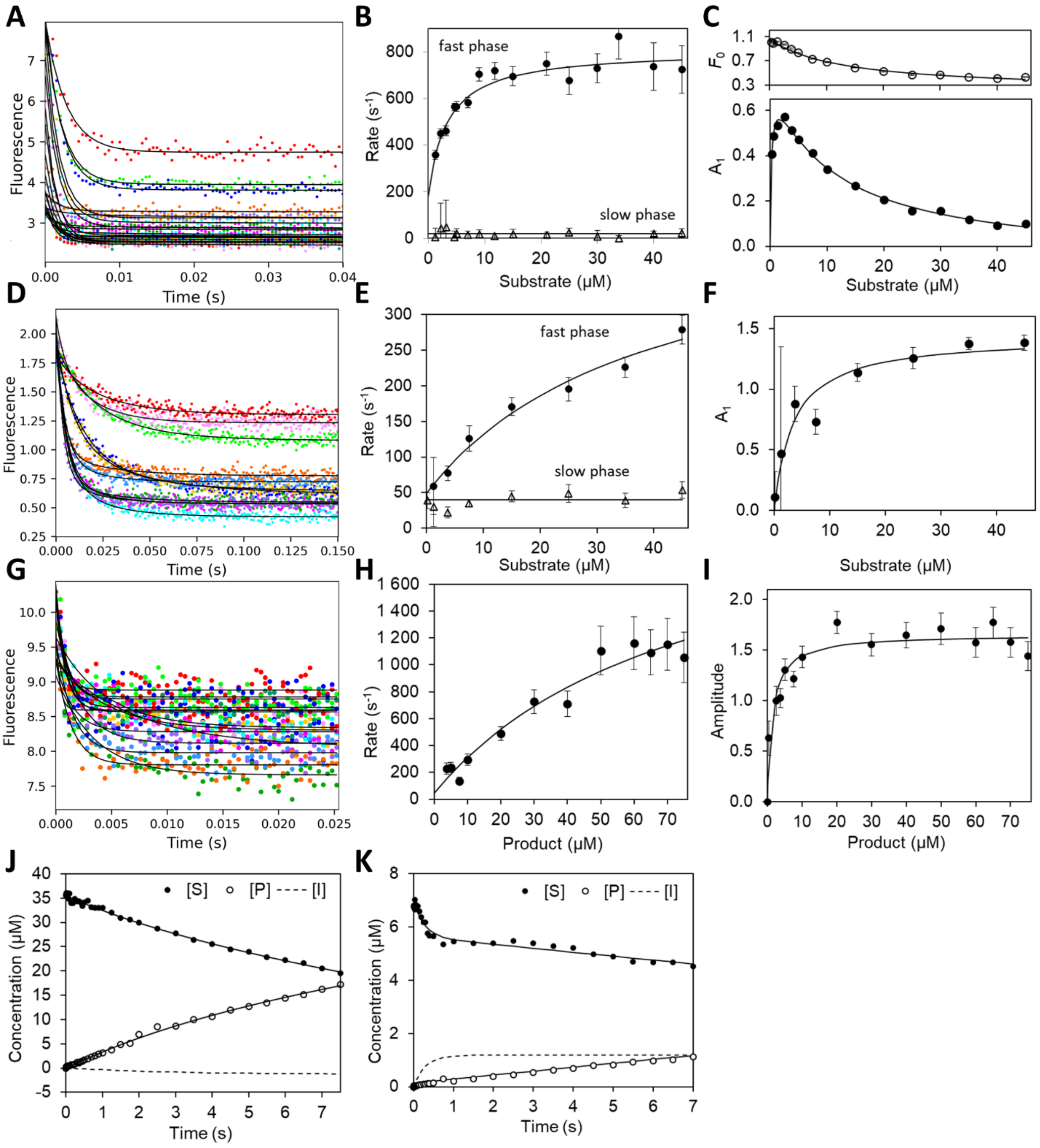

Figure S7. Conventional fitting of *ZenA$_{Scfl}$* kinetic data.

The initial phase of the reaction was examined by stopped-flow fluorescence (excitation 275 nm, emission > 320 nm) upon mixing of 0.5 – 45 μM ZEN with 2 μM *ZenA$_{Scfl}$* at 37°C (**A**) and 10°C (**D**). The solid lines represent fit to a double exponential function (Equation 1). The concentration dependence of the rates of the fast and slow phases derived from the double-exponential fitting of the data recorded at 37°C (**B**) and

10°C (**E**), the solid lines represent the best fit to hyperbola, and error bars show the standard errors. The concentration dependence of the initial fluorescence (F_0) and the amplitude of the fast phase (A_1) for the reaction at 37°C (**C**) and 10°C (**F**). (**G**) The stopped-flow fluorescence traces were recorded upon binding of 0.5 to 75 µM HZEN with 2 µM $ZenA_{Scfl}$ at 37°C. The solid lines represent fit to a single exponential function. The concentration dependence of the rate (**H**) and amplitude (**I**) derived from the single exponential fitting data in **G**. The solid lines represent the fit to hyperbola, and error bars show the standard errors. The reaction burst was analyzed using the rapid-quench-flow method upon mixing substrate 35 µM ZEN with 7.5 µM $ZenA_{Scfl}$ at 37°C (**J**) and 7 µM ZEN with 12.5 µM $ZenA_{Scfl}$ at 10°C (**K**). The solid lines represent the fit of the time course of ZEN and HZEN concentration to **Equation 4** and **5**, respectively. The dashed line represents the simulated concentration of the reaction intermediate [I], calculated as the difference between the amount of substrate consumed and product formed, since it could not be measured directly by any analytical method. At a given time t, $[I]_t = ([S]_0 - [S]_t) - [P]_t$, where $[S]_0$ is the initial concentration of the substrate, and $[S]_t$ and $[P]_t$ are the concentrations of substrate and product at time t, respectively.

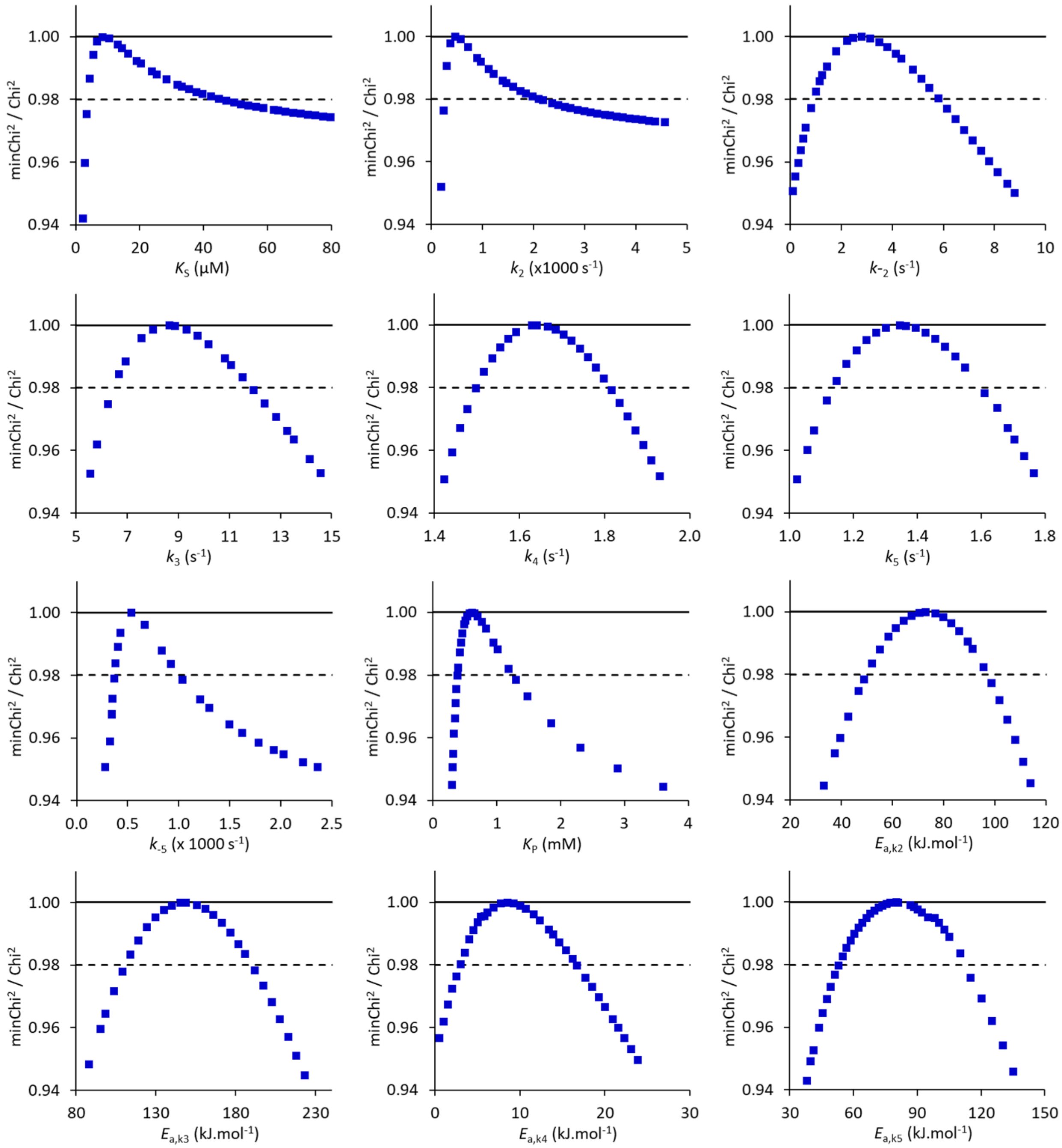

Figure S8. Confidence contour analysis of *ZenA*$_{Scfl}$ kinetic parameters.

Error analysis of the fitted parameters on the kinetic constants and energy barriers obtained from the global fit of *ZenA*$_{Scfl}$ kinetic data. Individual figures represent the dependence of the error on the respective fitted parameter while varying all other parameters to achieve the best fit. In every case, the dashed line shows the $\chi 2$ threshold (0.98) used to establish confidence intervals as reported in **Table S2** (Supporting Information).

A

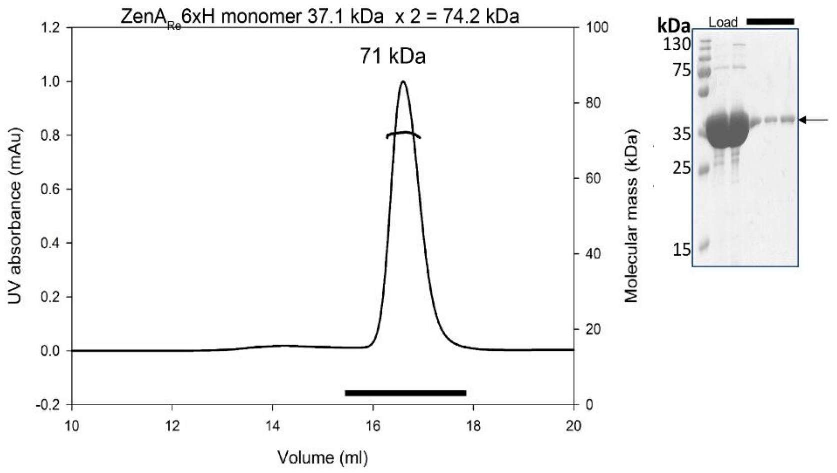

B

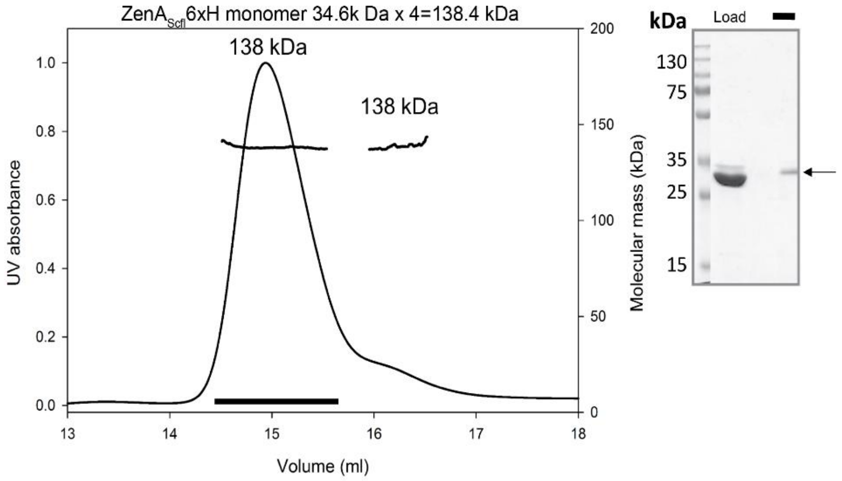

Figure S9. SEC-MALS analysis of oligomeric state of ZenA$_{Re}$ and ZenA$_{Scfl}$.

(**A**) ZenA$_{Re}$ eluted as a dimer. (**B**) ZenA$_{Scfl}$ eluted as a tetramer.

Table S3. Data collection and refinement statistics for ZenA structures.

Values for highest-resolution shell in parenthesis. Low high resolution shell completeness and I/σ values due to using CC1/2 cut-off criteria as proposed by Karplus and Diederichs [1], during data processing.

	$ZenA_{Re}$	$ZenA_{Re}$	$ZenA_{Re}$	$ZenA_{Scfl}$	$ZenA_{Scfl}$	$ZenA_{Scfl}$ H286Y	$ZenA_{Scfl}$ H286Y
PDB ID	**8CLT**	**8CLU**	**8CLV**	**8CLN**	**8CLO**	**8CLP**	**8CLQ**
Ligand	-	ZLAEN	HZEN	-	-	-	HZEN
Method	Native	Native	Native	SAD	Native	Native	Native
Beamline	ID29 (ESRF)	I03 (Diamond)	I04 (Diamond)	ID23-1 (ESRF)	ID23-2 (ESRF)	ID30B (ESRF)	ID29 (ESRF)
Temperature (K)	100	100	100	100	100	100	100
Wavelength (Å)	0.976250	0.976250	0.9795	0.977	0.8726	1.000000	0.970000
Resolution range (Å)	47.53 - 1.46 (1.512 - 1.46)	47.59 - 1.8 (1.864 - 1.8)	47.05 - 2.54 (2.631 - 2.54)	46.91 - 2.5 (2.582 - 2.5)	46.31 - 1.4 (1.45 - 1.4)	42.57 - 1.92 (1.989 - 1.92)	46.77 - 1.53 (1.585 - 1.53)
Space group	$C\ 2\ 2\ 2_1$	$C\ 2\ 2\ 2_1$	$C\ 2\ 2\ 2_1$	$P\ 3_1\ 2\ 1$	C 1 2 1	$P\ 2_1\ 2_1\ 2_1$	$P\ 2_1\ 2_1\ 2_1$
Unit cell (Å, °)	a=64.47<br>b=121.80<br>c=172.36<br>α=90.0<br>β= 90.0<br>γ=90.0	a=64.56<br>b=121.84<br>c=172.63<br>α=90.0<br>β= 90.0<br>γ=90.0	a=123.38<br>b=172.09<br>c=136.13<br>α=90.0<br>β= 90.0<br>γ=90.0	a=75.39<br>b=75.39<br>c=404.420<br>α=90.0<br>β= 90.0<br>γ=120.0	a=173.548<br>b=69.297<br>c=64.423<br>α=90.0<br>β= 96.06<br>γ=90.0	a=63.569<br>b=126.128<br>c=128.707<br>α=90.0<br>β= 90.0<br>γ=90.0	a=63.809<br>b=129.867<br>c=134.822<br>α=90.0<br>β= 90.0<br>γ=90.0
No. of molecules/AU	2	2	4	4	2	4	4
Solvent content (%)	46.4	47.1	49.6	47.5	54.8	32.5	39.5
No. of reflections (total / unique)	689372 (32032) / 114638 (9488)	426375 (43358) / 63294 (6223)	465130 (43353) / 44439 (4434)	915886 (88411) / 47746 (4683)	617138 (51653) / 148595 (14556)	251400 (24090) / 76545 (7561)	1146939 (98340) / 174008 (17042)
R merge (%)	0.1014 (4.677)	0.158 (3.112)	0.239 (1.626)	0.1202 (0.723)	0.1197 (1.534)	0.1096 (0.766)	0.2045 (4.244)
R meas (%)	0.1108 (5.527)	0.1713 (3.362)	0.2643 (1.811)	0.1235 (0.743)	0.137 (1.801)	0.1282 (0.9079)	0.222 (4.664)
Rpim (%)	0.04401 (2.872)	0.06529 (1.26)	0.1101 (0.7767)	0.0282 (0.170)	0.06537 (0.9222)	0.06419 (0.4712)	0.08555 (1.902)
CC1/2	0.999 (0.109)	0.998 (0.231)	0.99 (0.385)	0.999 (0.96)	0.993 (0.298)	0.996 (0.551)	0.997 (0.0712)
CC*	1 (0.443)	1 (0.612)	0.998 (0.745)	1 (0.99)	0.998 (0.678)	0.999 (0.843)	0.999 (0.365)
Multiplicity	6.0 (3.4)	6.7 (6.9)	10.5 (9.8)	19.2 (18.9)	4.2 (3.5)	3.3 (3.2)	6.6 (5.8)

Completeness (%)	92.98 (39.33)	99.85 (99.42)	92.26 (92.95)	99.94 (99.98)	99.52 (97.86)	95.93 (95.56)	98.31 (85.93)
I/σ(I)	10.20 (0.18)	8.87 (0.50)	9.63 (1.37)	20.75 (4.86)	6.18 (0.86)	8.22 (1.34)	7.01 (0.37)
Wilson B-factor	22.58	22.25	47.85	44.17	16.26	25.38	21.44
**Refinement**							
Reflections used (refinement / R-free)	109245 (4567) / 1895 (83)	63272 (6219) / 3139 (269)	44325 (4405) / 1999 (199)	47730 (4682) / 2001 (196)	148542 (14520) / 2000 (196)	76512 (7558) / 1992 (199)	171289 (14786) / 1969 (167)
Rwork/Rfree	0.1643 / 0.1993	0.1649 / 0.1976	0.2142 / 0.2461	0.1831 / 0.2322	0.1869 / 0.2157	0.1973 / 0.2501	0.1975 / 0.2165
RMSD, bonds (Å)	0.009	0.010	0.003	0.004	0.009	0.002	0.004
RMSD, angles (°)	1.01	1.12	0.58	0.72	1.01	0.51	0.74
Ramachandran favoured (%)	98.02	98.04	98.08	96.83	97.71	97.21	97.51
Ramachandran allowed (%)	1.98	1.96	1.92	3.17	2.29	2.71	2.49
Ramachandran outliers (%)	0.00	0	0.00	0	0.0	0.09	0.00
Clashscore	1.99	1.56	2.79	3.54	1.25	1.69	1.66
Average B-factor (Å2)	28.92	27.62	47.66	53.95	22.33	31.89	27.51
Average B-factor macromolecules (Å2)	27.57	26.26	47.67	53.98	21.39	31.69	27.25
Average B-factor ligands (Å2)	54.90	30.98	50.93	-	32.23	-	29.37
Average B-factor solvent (Å2)	37.61	31.72	45.03	46.19	32.97	34.85	31.08

(1) Karplus, P. A.; Diederichs, K. Assessing and Maximizing Data Quality in Macromolecular Crystallography. Curr. Opin. Struct. Biol. 2015, 34, 60–68. https://doi.org/10.1016/j.sbi.2015.07.003.

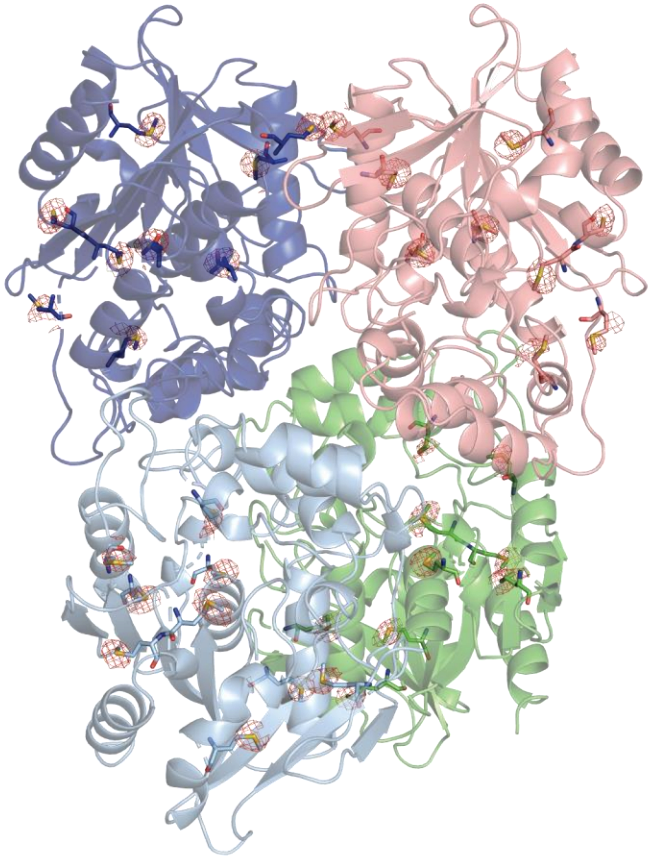

Figure S10. SeMet derivative structure of ZenA$_{Scfl}$ (PDB ID 8CLN).

ZenA$_{Scfl}$ SeMet derivative structure used for SAD phasing shown as cartoon, colored according to chains. Anomalous difference map contoured at 3 rmsd and shown as red mesh. Methionine residues shown as sticks colored according to chain.

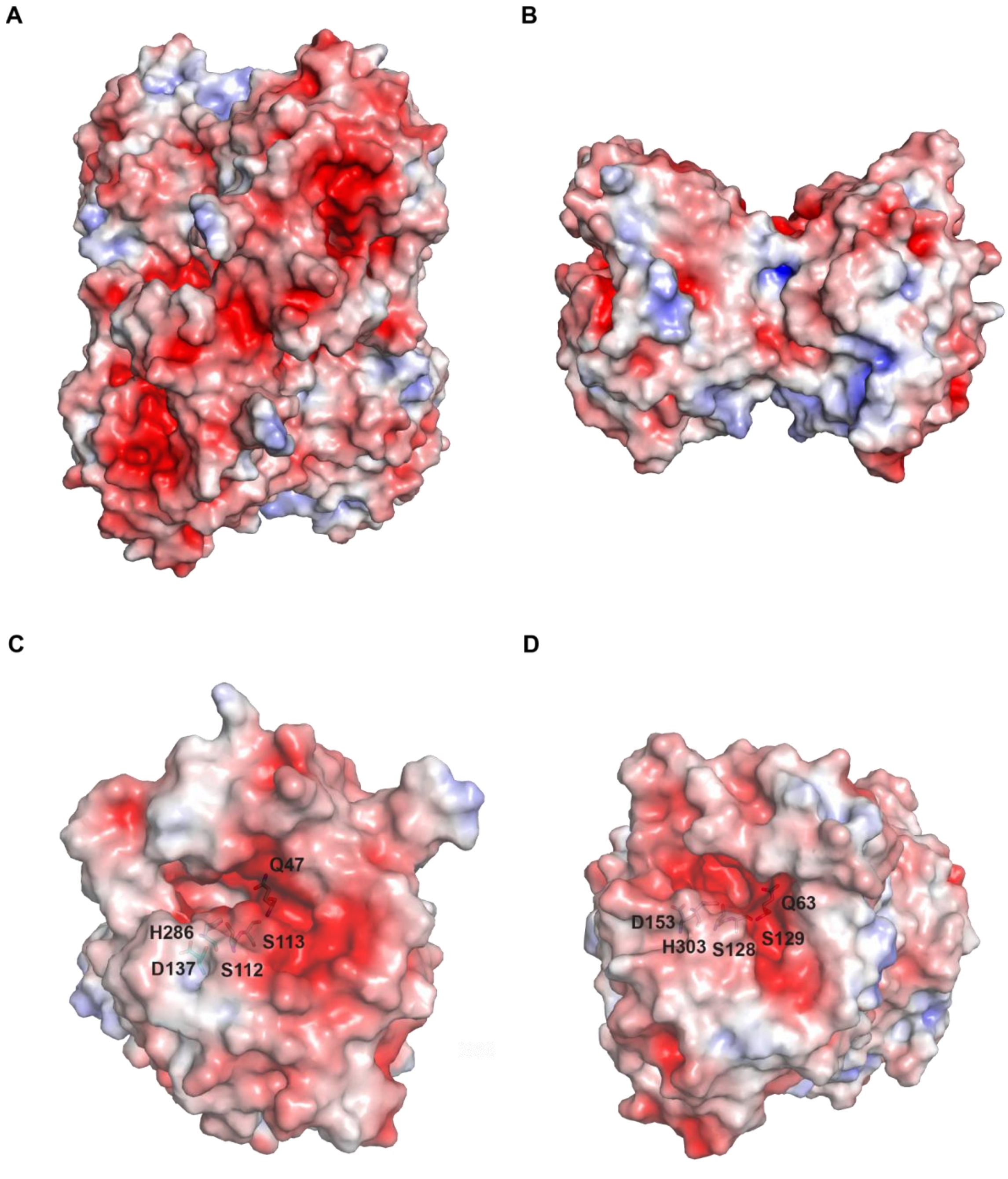

Figure S11. Electrostatic surfaces of $ZenA_{Scfl}$ and $ZenA_{Re}$.

(**A**) Electrostatic surface of $ZenA_{Scfl}$ reconstructed tetramer. (**B**) Electrostatic surface of $ZenA_{Re}$ dimer. (**C**) $ZenA_{Scfl}$ subunit showing the negatively charged active site cavity, active site and oxyanion hole residues shown as sticks. (**D**) $ZenA_{Re}$ subunit showing the negatively charged active site cavity, active site and oxyanion hole residues shown as sticks. Figures were generated with the APBS plugin for PyMol using a color ramp of -5 kB T/e (red), +5 kB T/e (blue).

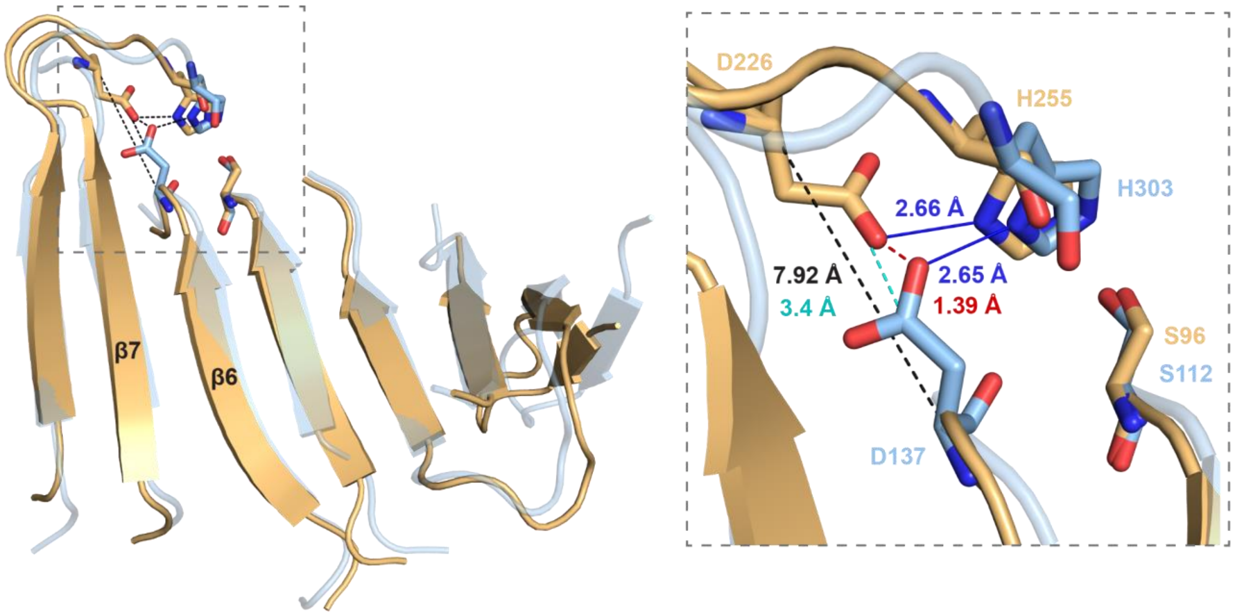

Figure S12. Structural alignment of catalytic triads of ZenA$_{Scfl}$ and CPO-L.

Left: Superposition of central beta sheets of CPO-L (orange) and ZenA$_{Scfl}$ (cyan) shown with cartoon representation. Catalytic triad residues are shown as sticks for ZenA$_{Scfl}$ (PDB ID: 8CLO, Ser-112/His-303/Asp-137) and CPO-L (PDB ID: 1A88, Ser-96/His-255/Asp-226). Inset: close up view of boxed region. Distances between atoms are shown as lines, labels are color coded according to distance. Although Asp-137 is located after β-strand 6 of ZenA$_{Scfl}$ and Asp-226 is located after β-strand 7 of CPO-L, the relative positions of hydrogen-bond receptor oxygen atoms in the catalytic triad are similar.

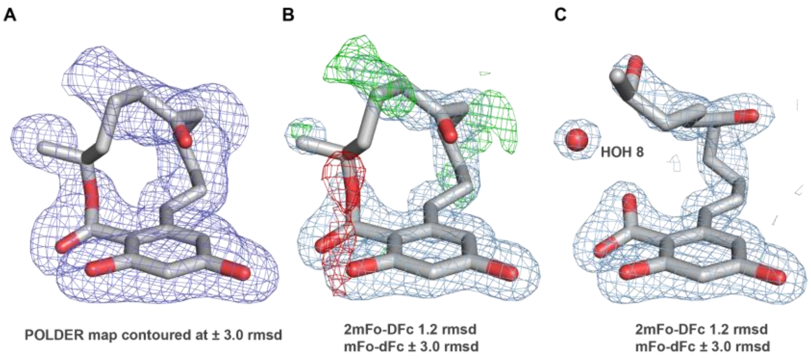

Figure S13. Ligand identification with $\text{ZenA}_{\text{Scfl}}$ H286Y (PDB ID: 8CLQ) dataset.

(**A**) POLDER map of ZEN modeled in the structure of $\text{ZenA}_{\text{Scfl}}$ H286Y. (**B**) 2Fo-Fc and Fo-Fc maps of ZEN modeled in the structure of ZenAScfl H286Y. (**C**) 2Fo-Fc map of HZEN modeled in the structure of $\text{ZenA}_{\text{Scfl}}$ H286Y.

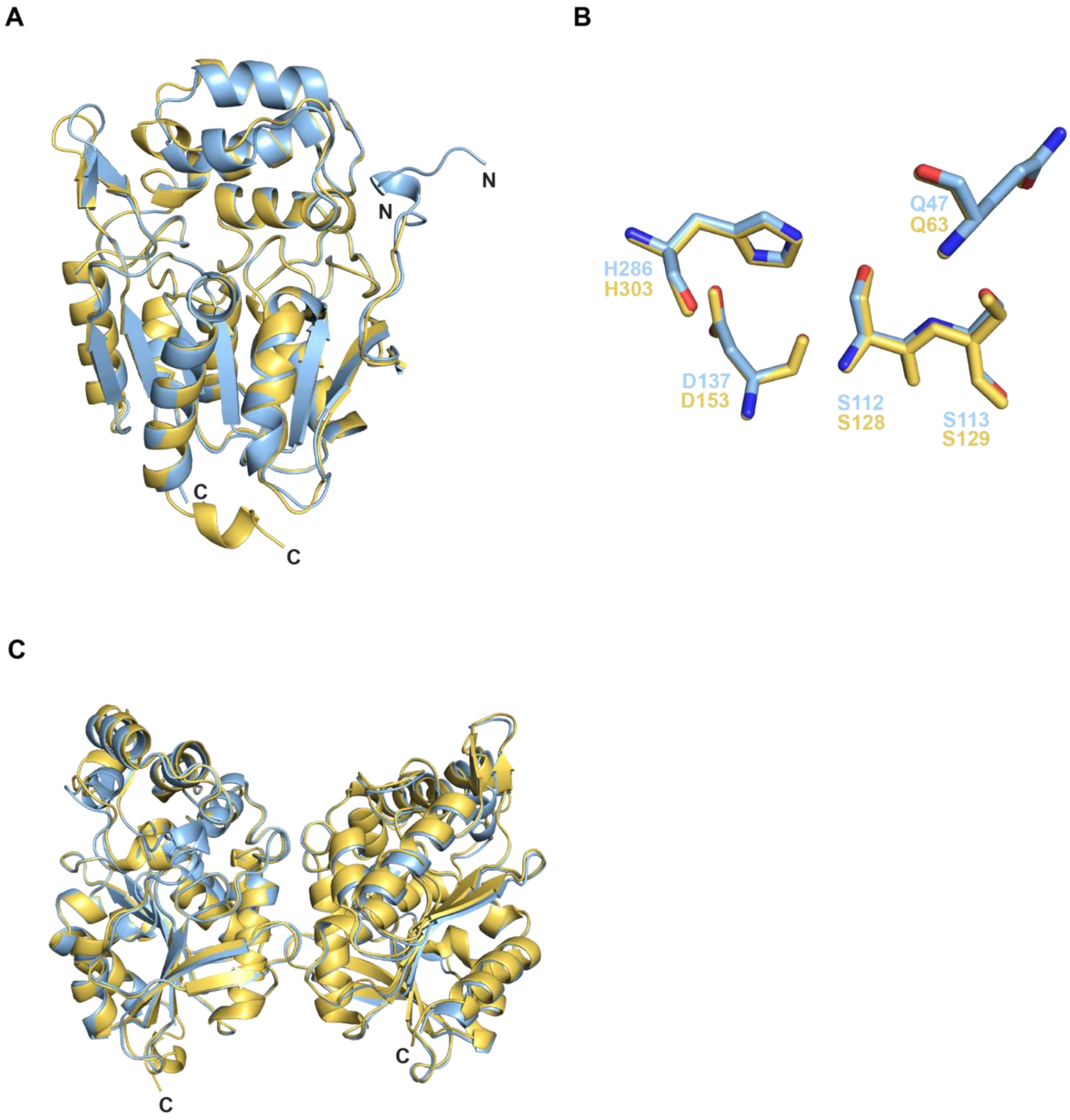

Figure S14. Structural alignment of ZenA$_{Scfl}$ and ZenA$_{Re}$.

ZenA$_{Scfl}$ is colored light blue, ZenA$_{Re}$ is colored yellow. Catalytic triad residues are shown as sticks. (**A**) alignment of monomers (chains A); (**B**) alignment of catalytic triad residues; (**C**) alignment of dimers (chains A and B).

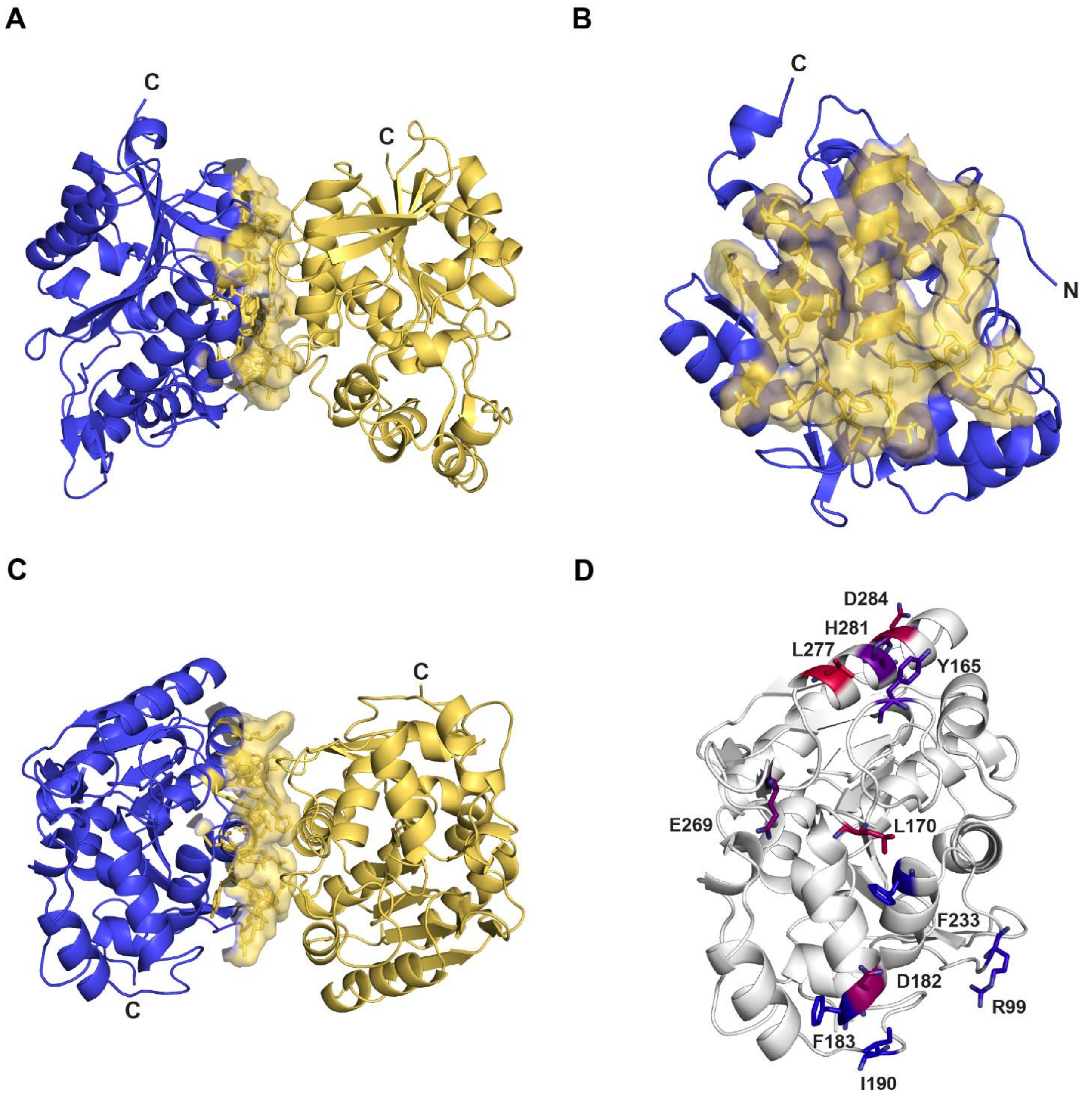

Figure S15. Oligomerization interfaces of ZenA$_{Re}$.

(**A**) Cartoon representation of ZenA$_{Re}$ dimer, interface residues are shown as sticks with surface representation. (**B**) Front view of the dimer interface after 90° rotation along y axis. (**C**) ZenA$_{Re}$ dimer as in **A**, shown from the bottom (90° rotation along x axis). (**D**) Bottom view of ZenA$_{Re}$ cartoon representation. Interface residues that are different from the matching residue of ZenA$_{Scfl}$ are shown as sticks, color coded according to their BLOSUM distance (blue: similar, red: dissimilar), and labeled.

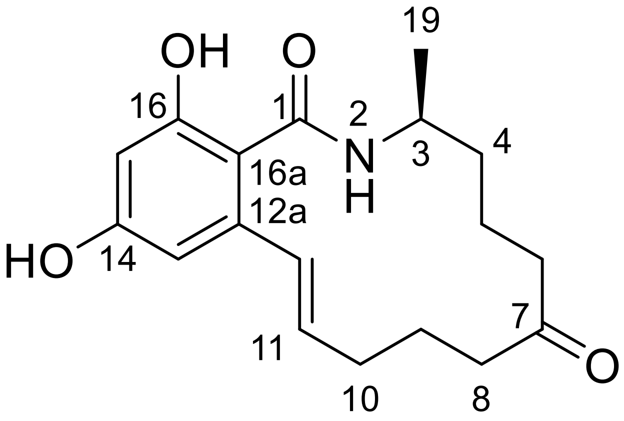

Figure S16. Structure of zearalactamenone.

(S,E)-14,16-dihydroxy-3-methyl-3,4,5,6,9,10-hexahydrobenzo[c][1]azacyclotetradecine-1,7(2H,8H)-dione, abbreviated ZLAEN, was synthesized for use as a ligand in X-ray crystallography.

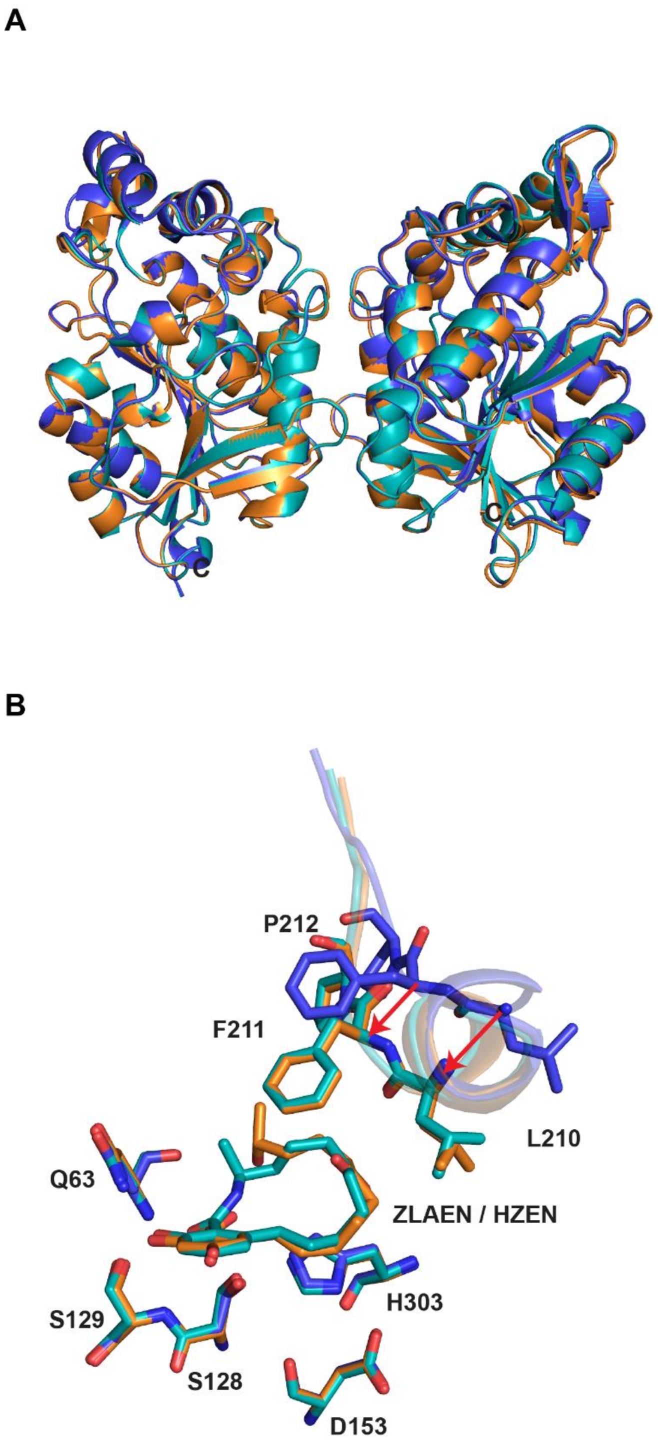

Figure S17. Comparison of apo and ligand bound structures of ZenA$_{Re}$.

A: Superposition of ZenA$_{Re}$ dimers shown in cartoon representation. Apo structure is colored blue (PDB ID: 8CLT), ZLAEN bound structure is colored teal (PDB ID: 8CLU), HZEN bound structure is colored orange (PDB ID: 8CLV). **B**: Stick representation of active site and cap residues. Color coding as in A. Cα shifts are highlighted with red arrows.

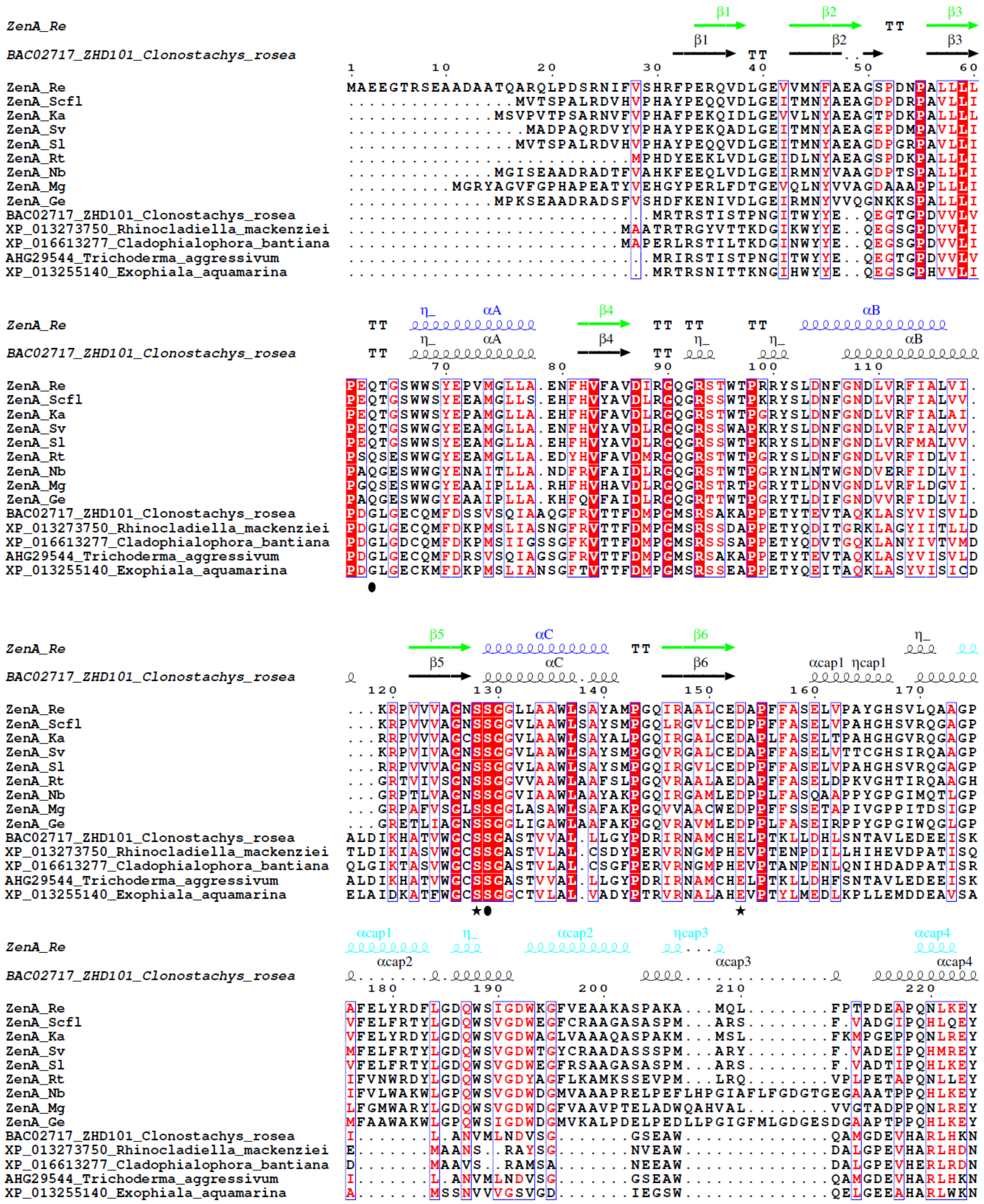
ZenA_Re
BAC02717_ZHD101_Clonostachys_rosea
ZenA_Scfl
ZenA_Ka
ZenA_Sv
ZenA_Sl
ZenA_Rt
ZenA_Nb
ZenA_Mg
ZenA_Ge
XP_013273750_Rhinocladiella_mackenziei
XP_016613277_Cladophialophora_bantiana
AHG29544_Trichoderma_aggressivum
XP_013255140_Exophiala_aquamarina

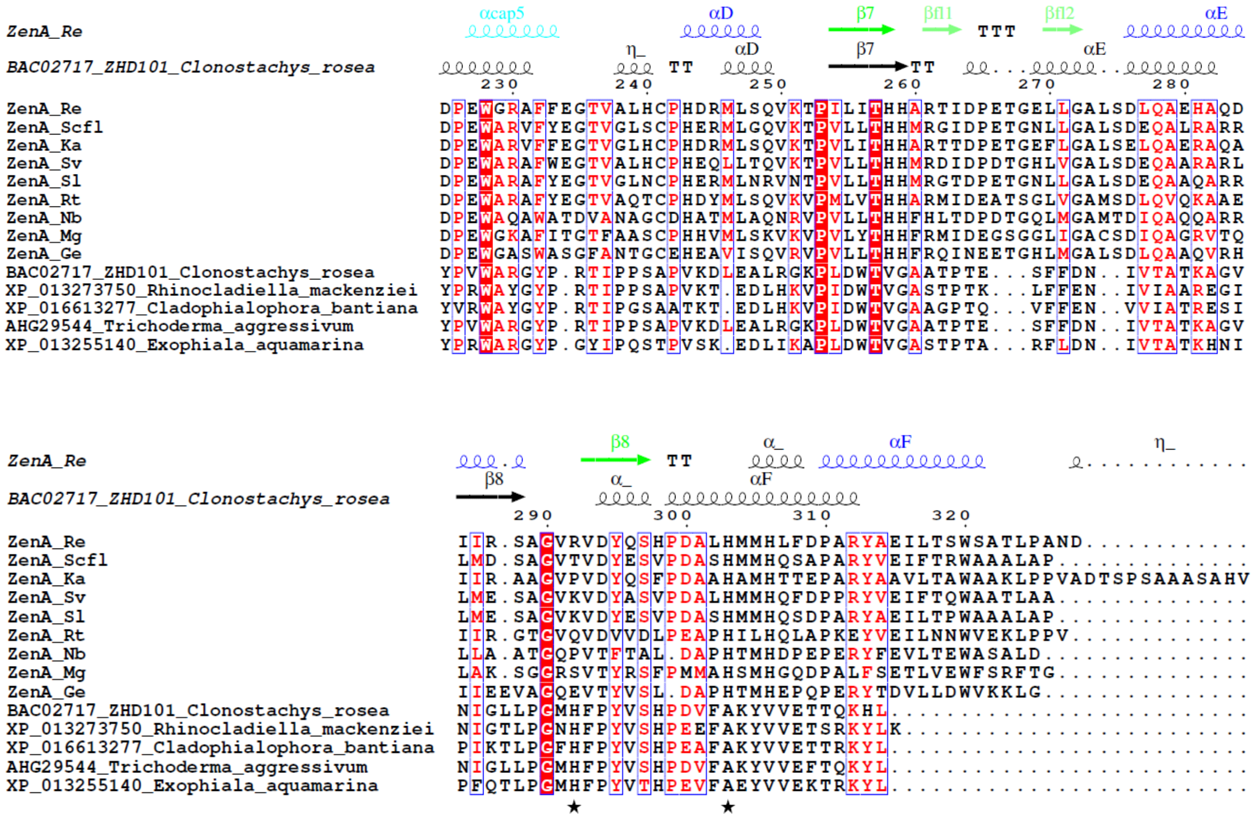

Figure S18. Alignment of bacterial ZenA with fungal ZHD ZEN lactonases.

Secondary structure elements shown for $ZenA_{Re}$ (PDB ID: 8CLT) and ZHD101 (PDB ID: 3WZL) are from chains A of the PDB files and labeled according to Ollis *et al.* [1]. The positions of residues of the catalytic triads are marked with stars, and the positions of residues forming the oxyanion hole are marked with circles (note that the catalytic histidines are not aligned). Identical amino acids are shown with red background. Red letters in blue frames indicate amino acids with more than 70% physicochemical similarity across the set of sequences. TT indicates a β-turn, TTT indicates an α-turn, and η indicates a 3_{10}-helix. Figure prepared with ESPript 3.0 [2].

(1) Ollis, D. L.; Cheah, E.; Cygler, M.; Dijkstra, B.; Frolow, F.; Franken, S. M.; Harel, M.; Remington, S. J.; Silman, I.; Schrag, J.; Sussman, J. L.; Verschueren, K. H. G.; Goldman, A. The a/β Hydrolase Fold. 1992, 5 (3), 197–211.

(2) Robert, X.; Gouet, P. Deciphering Key Features in Protein Structures with the New ENDscript Server. Nucleic Acids Res. 2014, 42 (W1), 320–324. https://doi.org/10.1093/nar/gku316.

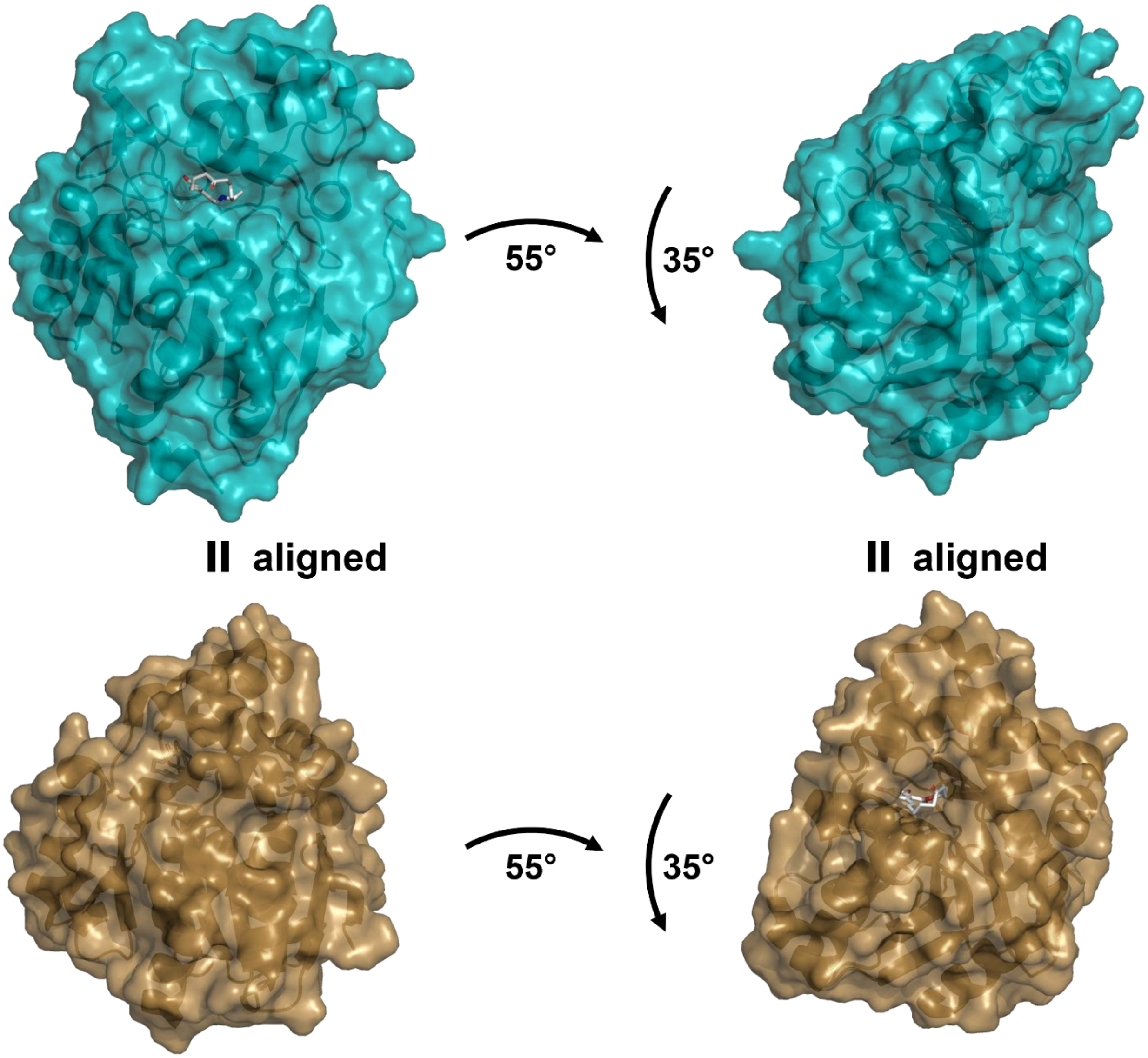

Figure S19. Comparison of substrate access paths to active sites of ZenA$_{Re}$ and ZHD101.

Monomers of ZenA$_{Re}$ with bound ZLAEN (8CLU, color teal, top) and ZHD101 S103A with bound ZEN (3WZM [1], color sand, bottom) were aligned and positioned to show ligand bound at the active site of ZenA$_{Re}$ (left), and both monomers were rotated by 55° about the y-axis and by 35° about the x-axis to show ligand bound at the active site of ZHD101 (right).

(1) Peng, W.; Ko, T.-P.; Yang, Y.; Zheng, Y.; Chen, C.-C.; Zhu, Z.; Huang, C.-H.; Zeng, Y.-F.; Huang, J.-W.; Wang, A. H.-J.; Liu, J.-R.; Guo, R.-T. Crystal Structure and Substrate-Binding Mode of the Mycoestrogen-Detoxifying Lactonase ZHD from Clonostachys Rosea. *RSC Adv.* **2014**, *4* (107), 62321–62325. https://doi.org/10.1039/C4RA12111B.
